# Diplumbane-catalysed solvent- and additive-free hydroboration of ketones and aldehydes†

**DOI:** 10.1039/d2ra03731a

**Published:** 2022-06-29

**Authors:** Guoqi Zhang, Sihan Li, Haisu Zeng, Shengping Zheng, Michelle C. Neary

**Affiliations:** Department of Sciences, John Jay College, PhD Program in Chemistry, The Graduate Center, The City University of New York New York NY 10019 USA guzhang@jjay.cuny.edu; Department of Chemistry, Hunter College, The City University of New York New York 10065 NY USA

## Abstract

A new diplumbane, namely [Pb(CH_2_SiMe_3_)_3_]_2_, was synthesized and structurally characterized. This group 14 element compound was found to catalyse the hydroboration of ketones and aldehydes under mild conditions without the use of additives and solvents, leading to the synthesis of a range of alcohols in high yields after hydrolysis.

## Abstract 2

A group 14 compound, diplumbane [Pb(CH_2_SiMe_3_)_3_]_2_, was synthesized and characterized, and it catalytic application for the efficient hydroboration of ketones and aldehydes was demonstrated for the first time.

## Graphical abstract

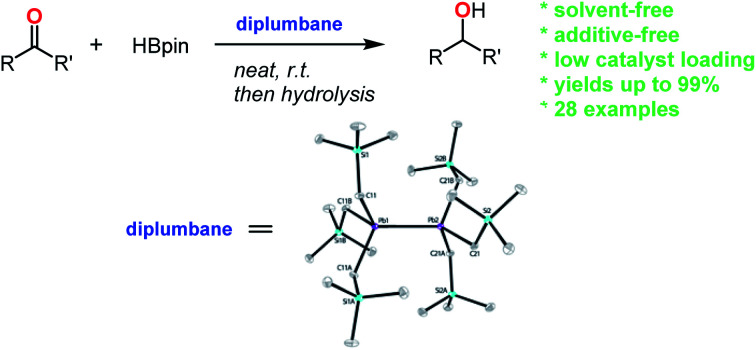

The reduction of carbonyl compounds is an important transformation for the synthesis of alcohols, which are ubiquitous in organic chemistry.^1,2^ The past decade has witnessed the development of numerous methods for this reaction including catalytic and non-catalytic ones, with particular interest in those involving pinacolborane (HBpin) as a reducing agent.^3,4^ Catalytic hydroboration of ketones and aldehydes using HBpin provides a facile approach to various alcohols following easy hydrolysis of the corresponding boronate esters. In addition, this method has advantages such as superior chemoselectivity, low cost, and broad substrate scope over the traditional stoichiometric reduction.^4^ As a result, significant progress has been made with regards to the design and synthesis of effective catalysts for the hydroborative reduction of carbonyl compounds, which has been documented in several recent review articles.^5–8^ While early transition metal complexes (containing, for example, Ti,^9^ V,^10^ Mn,^11^ Fe,^12^ Co,^13^ Ni,^14^ Cu,^15^ and Zn^16^) have been popular choices of catalysts, attention was expanded to main group element catalysts in recent years. In addition to the highly efficient metal hydridotriphenylborate hydroboration catalysts containing alkali (Li, Na, K) and alkali earth (Mg) metals that were first introduced by Okuda's group,^17,18^ other groups led by Hill,^19^ Mulvey,^20^ and Sen^21^ have developed other useful catalysts with main group metals, including calcium. Moreover, the group 13 element aluminum has been used in organoaluminum hydrides or alkyl complexes for the hydroboration catalysis of ketones and aldehydes by the Roesky,^22^ Nembenna^23^ and our groups,^24^ respectively. Two examples of gallium-based catalysts were also reported by the Goicoechea^25^ and Hevia^26^ groups in 2021. Several catalysts utilizing group 14 elements (mainly Ge and Sn) were reported by the Jones,^27^ Zhao,^28^ and Nagendran^29^ groups, as well as a silane catalyst stabilized by an amidinate ligand, namely PhC(N^*t*^Bu)_2_SiHCl_2_, reported by the Sen group.^30^ These highlight the potential of main group elements for the development of active hydroboration catalysts.

In contrast, the heavier group 14 element, lead, was almost unknown as a hydroboration catalyst. In 2017, Wesemann and co-workers reported a class of Lewis pair complexes, PhCH(PPh_2_)M(Ar*) [M = Ge, Sn, or Pb; Ar* = 2,6-(2,4,6-^i^Pr_3_C_6_H_2_)_2_C_6_H_3_], which catalysed the hydroboration of a single aldehyde, hexanal (Scheme 1).^31^ However, no further demonstration of their catalytic activity towards other aldehydes and ketones was reported. Our recent interest in observing active hydroboration catalysts with a broad range of metals across the periodic table has led to the synthesis of an unprecedented diplumbane compound, namely [Pb(CH_2_SiMe_3_)_3_]_2_. Herein, we report the X-ray structure of this diplumbane and its application as an effective catalyst for the hydroboration of ketones and aldehydes under additive- and solvent-free conditions.

**Scheme 1:**
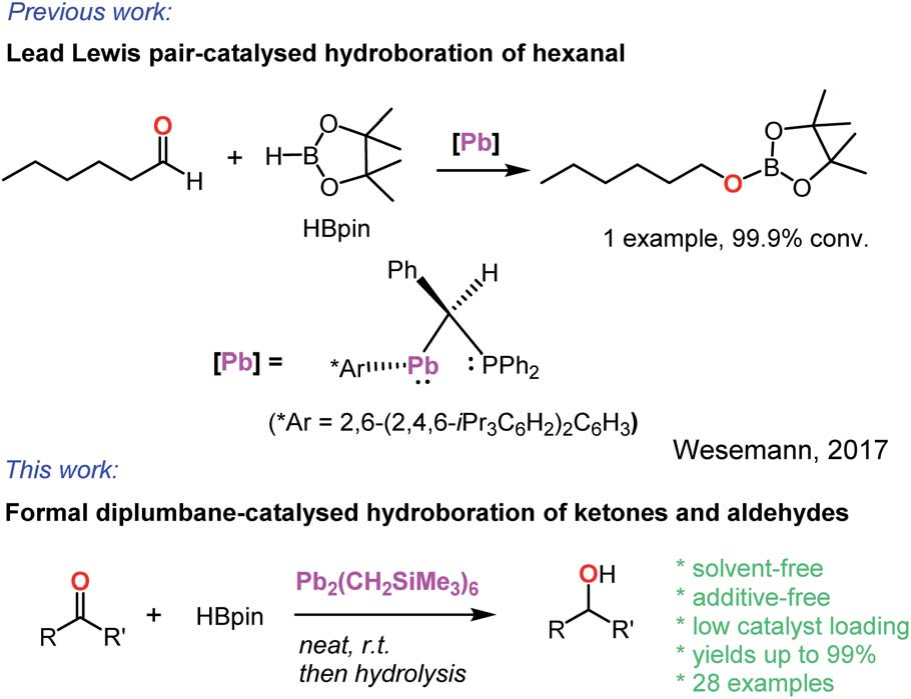
Lead-catalysed hydroboration of carbonyl compounds.

Our recent work has focused on the utilization of 2,2′;6′,2′′-terpyridine (tpy) for the synthesis of novel Co, Mn, V and Al complexes for reduction catalysis.^10,11*a*,13*a*,24^ When the reaction of tpy with equimolar PbCl_2_ was carried out in a THF solution, an insoluble white solid was obtained, which was isolated and characterized as (tpy)PbCl_2_ (see ESI†). (tpy)PbCl_2_ is insoluble in common organic solvents, but well soluble in dimethyl sulfoxide (DMSO). Attempt to crystallizing this complex from a mixture of DMSO/toluene solution was unsuccessful; instead, an inorganic polymer [Pb_2_Cl_4_(DMSO)_3_]_*n*_ was isolated without the incorporation of tpy ligand, indicating a M-tpy dissociation has occurred during crystallization, according to X-ray structural analysis (see ESI†). Solid sample of (tpy)PbCl_2_ was further used to react with LiCH_2_SiMe_3_ (2.2 equiv.) in diethyl ether for 4 h to give a pale-yellow solution after filtration. The concentrated reaction mixture was then cooled to −28 °C, and bright yellow block-like crystals of 1 were obtained in 46% yield (based on Si) after one week. Replacing the tpy ligand with 4′-Cl-tpy for the synthesis led to the isolation of 1 in a similar yield (48%). In addition, two independent attempts to prepare 1 by reacting PbCl_2_ with LiCH_2_SiMe_3_ without the presence of tpy were unsuccessful, leading to unidentified oil. This indicates the significant role played by tpy ligands. It is believed that during the formation of 1, a process involving the oxidation of Pb from Pb^II^ to Pb^III^ occurred, along with the possible reduction of tpy ligand. This is consistent with the well-known redox-active nature of tpy.^10^ However, attempts to isolating the reduced product of tpy were unsuccessful, probably owing to its poor stability. 1 was characterized by IR, elemental analysis and solution NMR spectroscopy (see ESI†). The solid-state structure was further revealed by X-ray crystallography. X-ray diffraction analysis confirmed that 1 crystallizes in the trigonal space group *P*3. The molecular structure of 1 is shown in Scheme 2. The structure features a dinuclear lead hexaalkyl compound, a diplumbane reminiscent of the known compound, [PbMe_2_(CH_2_SiMe_3_)]_2_, reported by Pannell in 1994, synthesized from (CH_2_SiMe_3_)Me_2_PbBr and Mg or (Ph_3_Sn)Li with yields of 7% and 18%, respectively.^32^ Our use of tpy as a ligand template using PbCl_2_ as a starting material both simplifies the synthesis of this type of diplumbane and improves the yield, introducing a new synthetic methodology for this type of compounds. In 1, both Pb centers adopt a slightly distorted tetrahedral geometry with *τ*_4_ parameters of 0.92 and 0.93, respectively, where a value of 1.00 represents a perfect tetrahedron.^33^ The Pb–Pb bond distance is 2.89922(19) Å, slightly shorter than that in [PbMe_2_(CH_2_SiMe_3_)]_2_ (2.968(2) Å), yet comparable to those observed in several other R_3_Pb–PbR_3_ compounds (Pb–Pb bond lengths range from 2.839(2) Å to 2.908(2) Å).^34^ The Pb–C bond lengths are also close to those reported.^34^

**Scheme 2:**
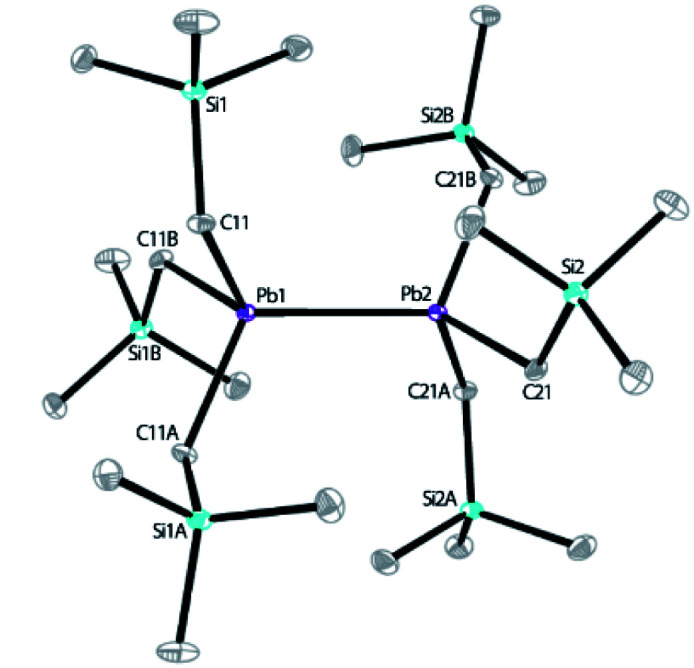
The X-ray structure of diplumbane 1 with ORTEP diagram drawn at 30% thermal ellipsoids probability level. Selected bond lengths (Å): Pb1–Pb2 = 2.89922(19), Pb1–C11 = 2.260(8), Pb2–C21 = 2.243(8); and angles (°): C11–Pb1–Pb2 = 115.0(2), C11–Pb1–C11A = 103.5(3), C21–Pb2–Pb1 = 114.2(2), C21–Pb2–C21A = 104.4(3).

1 was then examined as a catalyst for the hydroboration of ketones. Delightfully, it was found that the reaction of acetophenone with HBpin proceeded well in the presence of 0.25 mol% of 1, and quantitative conversion to the corresponding boronate ester was observed after 16 h under neat conditions at room temperature without the presence of additives. 1-Phenylethanol could be readily isolated in 94% yield after hydrolysis and purification *via* column chromatography with silica gel (2a, Scheme 3). A gram-scale synthesis of 2a was also carried out to confirm the practical usability of this catalyst. The efficacy of 1 is therefore well comparable to those well-performed transition metal catalysts reported for this reaction.^9–16^ In addition, a control experiment using PbCl_2_ instead of 1 led to only 5% gas chromatography (GC) yield of the corresponding boronate ester under the same conditions. Next, we applied this method to a broader range of ketones, featuring aromatic, aliphatic and cyclic substrates. The results are illustrated in Scheme 3. Acetophenones bearing halo groups were hydroborated successfully, affording the secondary alcohols 2b and 2c in 90% and 88% isolated yields, respectively. Both electron-donating and -withdrawing groups did not affect the catalytic activity (2d–f). In addition, functionalized ketones such as cyclopropyl phenyl ketone and α,β-unsaturated ketone were reduced selectively on the ketone site to give 2g and 2h with good yields. More challenging diaryl ketones worked as well for the hydroboration catalysed by 1 (2j and 2k). Finally, aliphatic and cyclic ketones were also used as substrates, and similar reactivity was observed (2l–o). However, either 3-acetylpyridine (for 2p) or 2-acetylpyridine was found to be almost inactive for this reaction, likely due to its coordination with 1 leading to deactivation of the reactive intermediate.

**Scheme 3:**
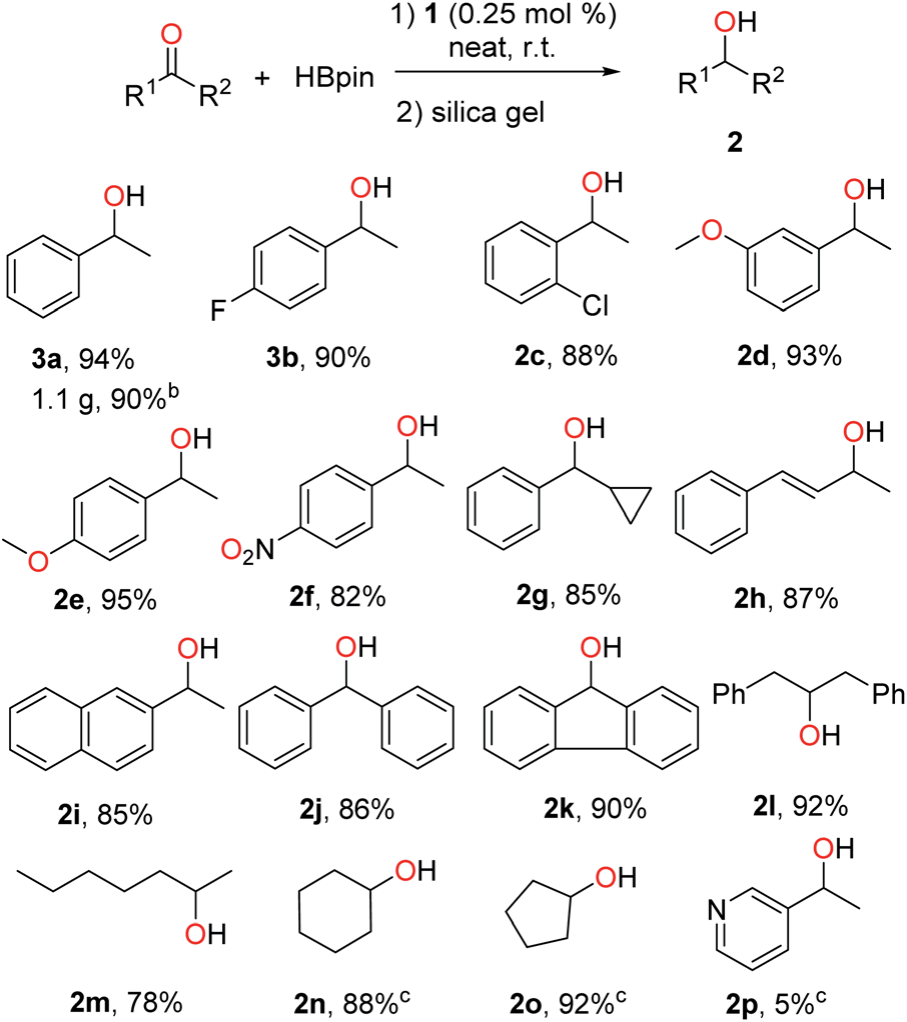
Lead-catalysed hydroboration of ketones.^a a^ Conditions: 1 (0.25 mol%), ketone (1.0 mmol) and HBpin (1.1 mmol), neat, 25 °C, 16 h, N_2_. Yields of isolated alcohol products after column chromatography. ^b^ Reaction run at a 10 mmol scale. ^c^ GC yields of borate esters without hydrolysis using hexamethylbenzene as internal standard.

1-Catalysed hydroboration was further extended to various aldehydes, and the results are summarized in Scheme 4. In general, benzaldehydes with halo, electron-donating or -withdrawing groups were hydroborated smoothly by 1 under neat conditions, affording primary alcohols (3a–g) in 84–96% yields. Cinnamaldehyde and aliphatic aldehydes were also active substrates for the hydroboration.

**Scheme 4:**
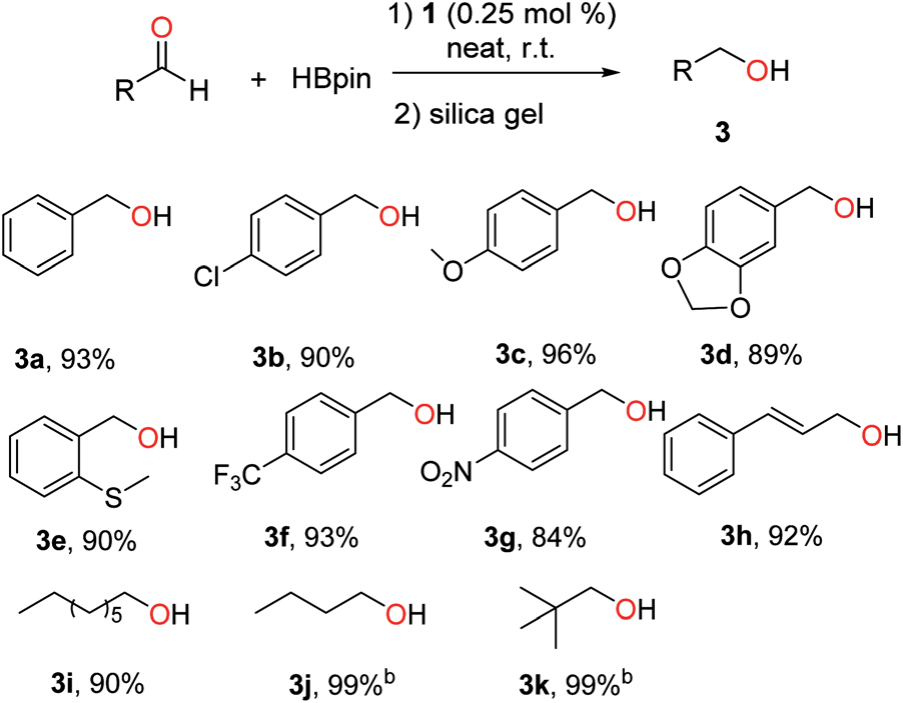
Lead-catalysed hydroboration of aldehydes.^a a^ Conditions: 1 (0.25 mol%), aldehyde (1.0 mmol) and HBpin (1.1 mmol), neat, 25 °C, 16 h, N_2_. Yields of isolated alcohol products after column chromatography. ^b^ GC yields of borate esters without hydrolysis using hexamethylbenzene as internal standard.

Next, chemoselective hydroboration was investigated with two reducible groups present. First, intermolecular competition experiments were carried out using benzaldehyde as a substrate in the presence of equimolar acetophenone or methyl benzoate. The results revealed that the aldehyde was selectively converted to the boronate ester, while the ketone and ester remained intact (Scheme 5). Then intramolecular competition reactions were conducted using methyl 4-formylbenzoate or diphenyl chalcone oxide with two reducible functionalities in each molecule. It was observed that the aldehyde or ketone was selectively reduced through hydroboration over the ester or epoxide, and boronate esters 4 and 5 were isolated without hydrolysis in 94% and 84% yields, respectively.

**Scheme 5:**
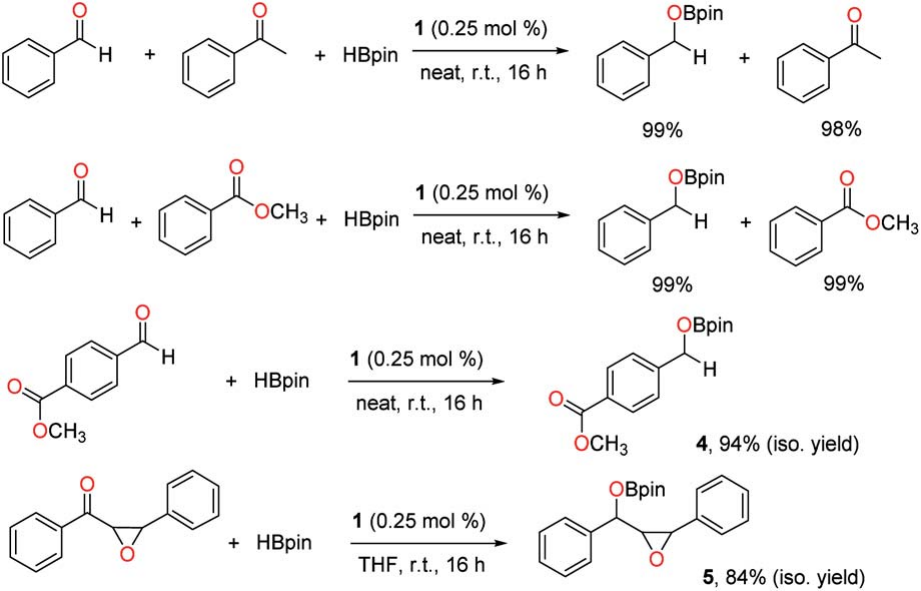
Chemoselective hydroboration catalysed by 1.

Since metal hydrides often behave as the active catalyst for transition and main-group metal catalysed hydroboration,^6,35^ we propose this as a possible mechanism for 1-catalysed hydroboration. Although attempts at isolating any reactive intermediate and/or a ketone substrate from the reactions of 1 with HBpin were unsuccessful, *in situ* NMR spectroscopy of the reaction of 1 with HBpin does show the formation of Me_3_SiCH_2_Bpin, which supports the formation of a possible hydride species I as shown in Scheme 6.^36^ Thus, we hypothesize that an insertion/σ-bond metathesis type mechanism might have been under operation (Scheme 6). While we propose a mechanism involving a simple lead hydride (I, Scheme 6) generated from the reaction of precatalyst (1) with HBpin, other possible polyhydride species cannot be excluded. Next, C=O insertion of carbonyl compound (ketone or aldehyde) into the Pb–H bond would lead to the formation of lead alkoxide III*via* intermediate II after σ-bond metathesis. The lead alkoxide would then react with HBpin to afford the boronate ester through intermediate IV, releasing the lead hydride I and completing the catalytic cycle.

**Scheme 6:**
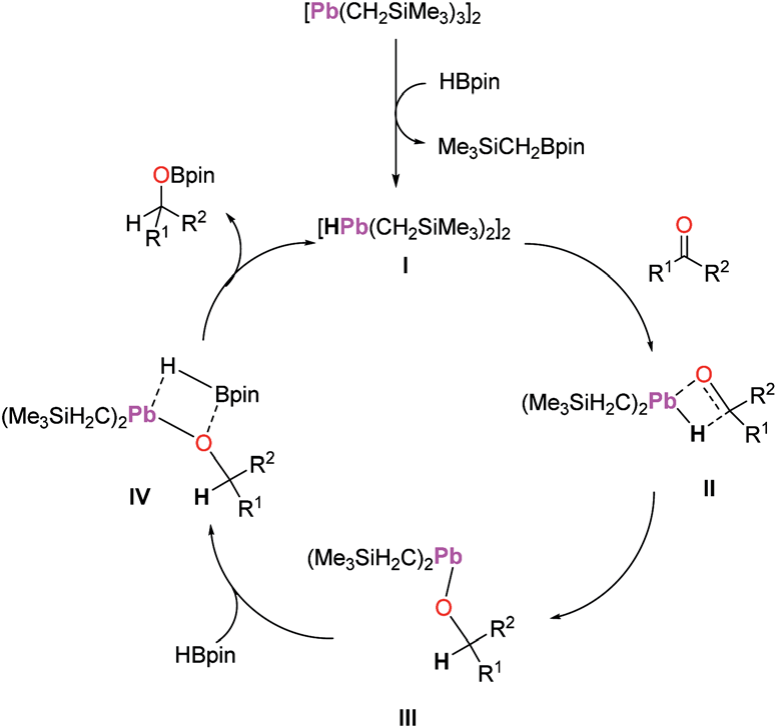
A plausible mechanism for the hydroboration of carbonyl compounds by 1 as a precatalyst. Only half of the Pb_2_ species are shown for intermediates II–IV for clarity.

## Conclusions

In summary, a new diplumbane compound [Pb(CH_2_SiMe_3_)_3_]_2_ was synthesized and structurally characterized. The group 14 compound was found to be a precatalyst for the efficient hydroboration of a range of ketones and aldehydes under additive- and solvent-free conditions at room temperature. The method shows excellent chemoselectivity for hydroboration of aldehyde over ketone or ester, and for ketone over epoxide. A plausible catalytic cycle was proposed in which a lead hydride undergoes C=O bond insertion/σ-bond metathesis. This represents the first example of formal hydroboration catalysis promoted by a diplumbane precatalyst.

## Conflicts of interest

There are no conflicts of interest to declare.

## Supplementary Material

RA-012-D2RA03731A-s001

RA-012-D2RA03731A-s002
