# Supplementary material for: Diplumbane-catalysed solvent- and additive-free hydroboration of ketones and aldehydes
Source: RSC Adv. 2022 Jun 29;12(30):19086–90. doi: 10.1039/d2ra03731a (PMC9241624; doi:10.1039/d2ra03731a)
Supplement: RA-012-D2RA03731A-s001 [file RA-012-D2RA03731A-s001.pdf]

Electronic Supplementary Material (ESI) for RSC Advances.
This journal is © The Royal Society of Chemistry 2022

Electronic Supplementary Information

Diplumbane-catalysed solvent- and additive-free hydroboration of ketones and aldehydes

Guoqi Zhang,*[,a] Sihan Li,[a,b] Haisu Zeng,[a,b] Shengping Zheng[b] and Michelle C. Neary[b]

[a] Department of Sciences, John Jay College and Ph.D. Program in Chemistry, The Graduate Center of the City University of New York, New York, 10019 NY, USA.
[b] Department of Chemistry, Hunter College, the City University of New York, New York, 10065 NY, USA.

Experimental Details

General Considerations. The synthesis of catalyst and catalytic reactions were carried out under a nitrogen atmosphere using standard glove-box techniques. Anhydrous grade solvents and liquid reagents used were obtained from Aldrich or Fisher Scientific and stored over 4 Å molecular sieves. FT-IR spectra were recorded on a Shimadzu 8400S instrument with solid samples under N_2 using a Golden Gate ATR accessory. ^{1}H NMR and ^{13}C NMR spectra were obtained at room temperature on a Bruker AV 500 or 600 MHz NMR spectrometer, with chemical shifts (δ) referenced to the residual solvent signal. For ^{207}Pb NMR experiments, $Pb(NO_3)_2$ (in D_2O) was used as a reference. GC-MS analysis was obtained using a Shimadzu GCMS-QP2010S gas chromatograph mass spectrometer. 2,2′;6′,2″-Terpyridine (tpy) was purchased from Sigma-Aldrich.

Synthesis of (tpy)PbCl₂. In a glovebox under nitrogen atmosphere, tpy (233.0 mg, 1.00 mmol) was dissolved in dry THF (8 mL) in a 20 mL scintillation vial. $PbCl_2$ (278 mg, 1.0 eq.) was then added as a powder. The suspension was stirred at room temperature overnight. White solid was collected and washed with THF (2 × 2 mL) and then dried under vacuum. The white solid is insoluble in common organic solvents such as CH_2Cl_2, $CHCl_3$, CH_3CN, THF and MeOH, but well soluble in DMSO. Yield: 488 mg (95%). FT-IR (solid, cm^{-1}): 2947m, 2893m, 1401m, 1353m, 1295w, 1242s, 981s, 821s, 709s, 582s. ^{1}H NMR (600 MHz, DMSO-d_6) δ 8.80 (ddd, J = 4.7, 1.8, 0.9 Hz, 2H), 8.66 (dt, J = 7.9, 1.1 Hz, 2H), 8.49 (d, J = 7.8 Hz, 2H), 8.16 (t, J = 7.8 Hz, 1H), 8.06 (ddd, J = 8.0, 7.5, 1.8 Hz, 2H), 7.56 (ddd, J = 7.4, 4.8, 1.2 Hz, 2H) ppm; ^{13}C NMR (101 MHz, $CDCl_3$) δ 154.9, 154.8, 149.3, 137.6, 124.5, 121.0 ppm. No ^{207}Pb NMR signal was observed. Anal. Calcd. for $C_{15}H_{11}Cl_2N_3Pb$: C 35.23, H 2.17, N 8.22%. Found C 34.88, H 2.07, N 8.05%. Note: slow diffusion of diethyl ether to a solution of (tpy)$PbCl_2$ in DMSO/toluene (2:1, v/v) over 3 days gave colorless block-like crystals which were identified as $[Pb_2Cl_4(DMSO)_3]_n$ by X-ray diffraction analysis (see below).

Synthesis of 1. In a glovebox under nitrogen atmosphere, (tpy)$PbCl_2$ (256 mg, 0.5 mmol) was suspended in diethyl ether (10 mL) in a 20 mL scintillation vial and $LiCH_2SiMe_3$ (94 mg, 1.00 mmol) was added in small portions. A pale yellow suspension developed and was allowed to stir for 4 hours at room temperature. The solution was filtered through a celite pad and the yellowish filtrate was evaporated to ca. 2 mL, and then was kept at -28 °C for one week. Yellow block-like crystals suitable for X-ray diffraction analysis were collected by decanting the solvent. The product was washed with pentane and dried in vacuo. Yield: 72 mg (46%). FT-IR (solid, cm^{-1}): 2947m, 2893m, 1401m, 1353m, 1295w, 1242s, 981s, 821s, 709s, 582s. ^{1}H NMR (600 MHz, toluene-d_8) δ 1.05 (s, 12H), 0.20 (s, 54H) ppm; ^{13}C NMR (126 MHz, toluene-d_8) δ 9.2, 2.9 ppm. ^{207}Pb NMR (84 MHz, toluene-d_8) δ -128.9 ppm. Anal. Calcd. for $C_{24}H_{66}Pb_2Si_4$: C 30.74, H 7.09%. Found C 31.02, H 7.31%. Note: occasionally, white crystals (with very small amount) could be observed on the wall during the crystallization, which has been identified to be the free tpy ligand as determined by its cell parameters via X-ray diffraction analysis.

General Procedure for 1-Catalysed Hydroboration of Ketones and Aldehydes. In a glovebox under nitrogen atmosphere, complex **1** (2.35 mg, 0.0025 mmol, 0.25 mol%) was placed in a 3.8 mL glass vial equipped with a stir bar. Ketone or aldehyde (1.0 mmol) and pinacolborane (140.8 mg, 1.1 mmol) were then added. The reaction mixture was allowed to stir at room temperature for 16 h. At completion of the reaction, the reaction was exposed to the air and the solvent was

evaporated. The product was purified through flash column chromatography with SiO_2 using ethyl acetate/hexane as an eluent. The alcohol products were characterized by ^1H and ^{13}C NMR spectroscopies.

Gram-scale synthesis of 2a. In a glovebox under nitrogen atmosphere, complex **1** (23.5 mg, 0.025 mmol, 0.25 mol%) was placed in a 3.8 mL glass vial equipped with a stir bar. Acetophenone (1.2 g, 10 mmol) and pinacolborane (1.41 g, 1.1 mmol) were then added. The reaction mixture was allowed to stir at room temperature for 16 h. At completion of the reaction, the reaction was exposed to the air and the solvent was evaporated. The product was isolated through a flash column chromatography with SiO_2 using ethyl acetate/hexane (1:10, v/v) as an eluent to give colorless oil. Yield: 1.1 g (90%).

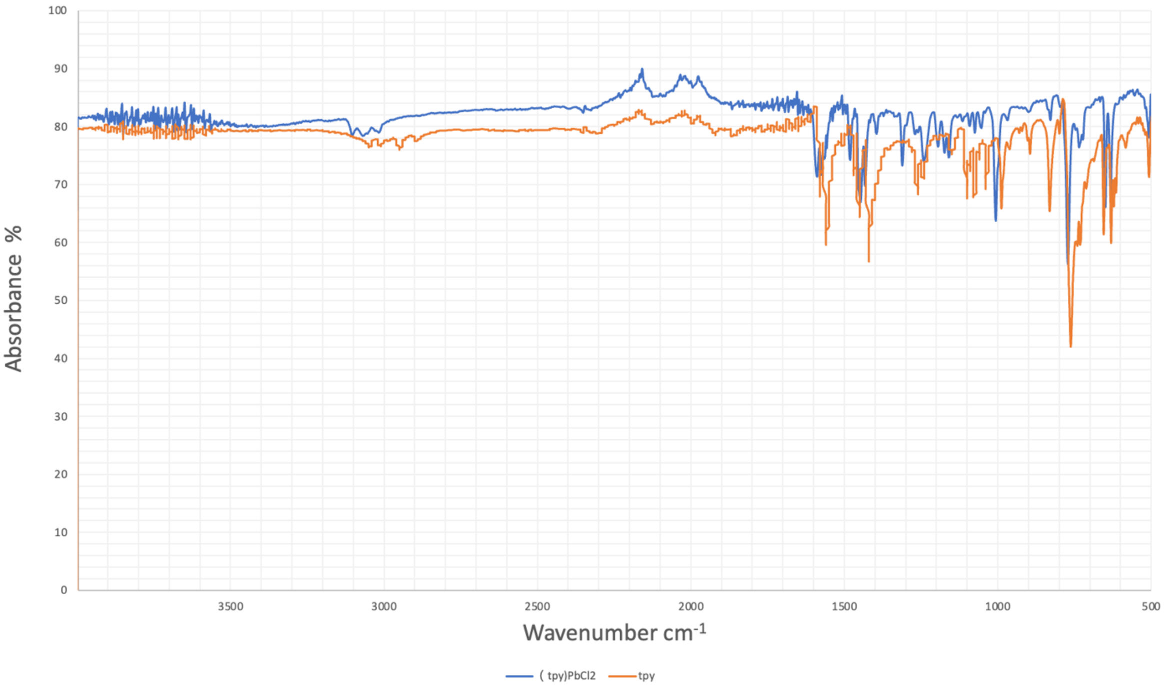

Fig. S1. The comparison of FT-IR spectra between tpy and (tpy)$PbCl_2$.

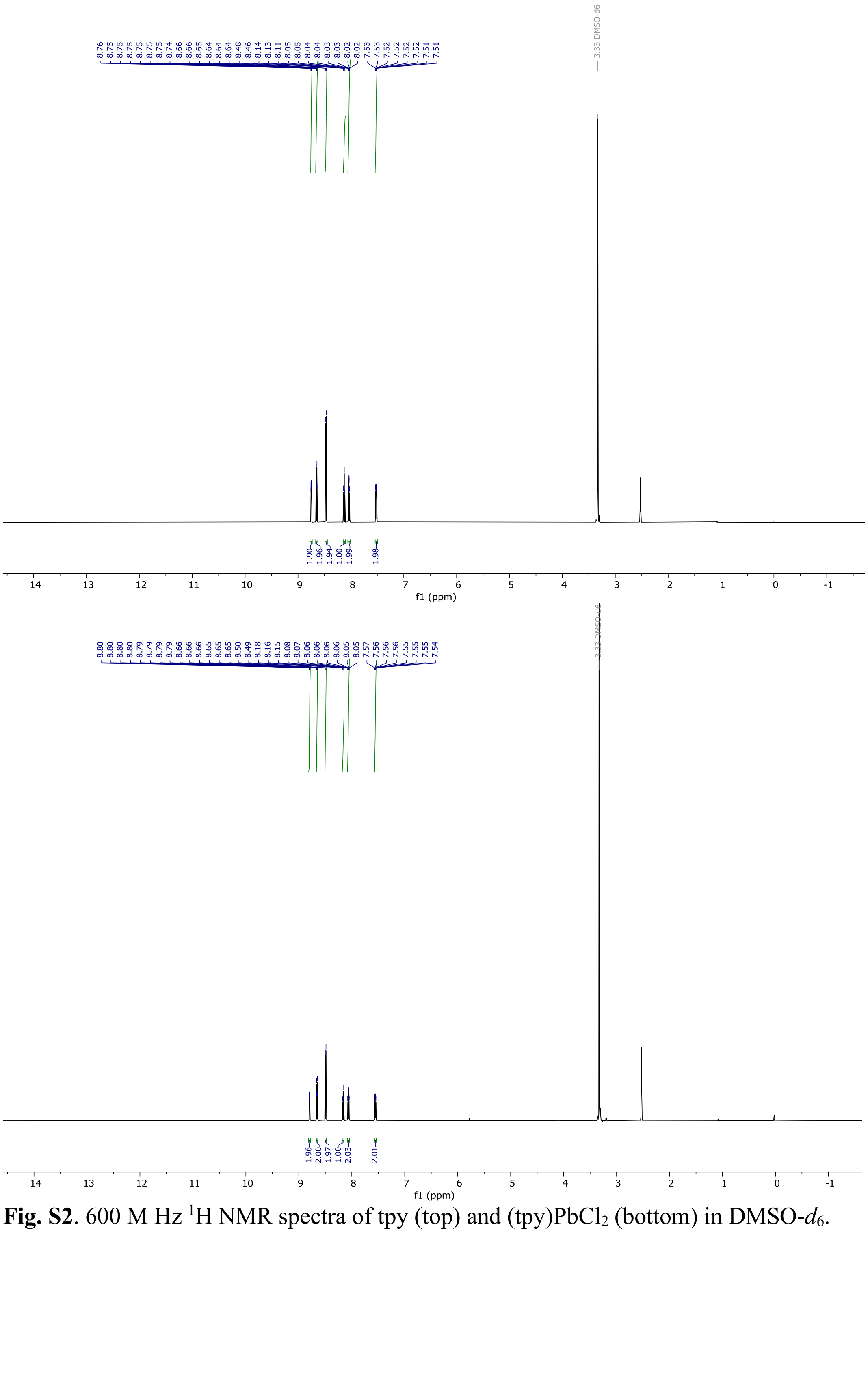

Fig. S2. 600 M Hz ^{1}H NMR spectra of tpy (top) and (tpy)$PbCl_2$ (bottom) in DMSO-d_6.

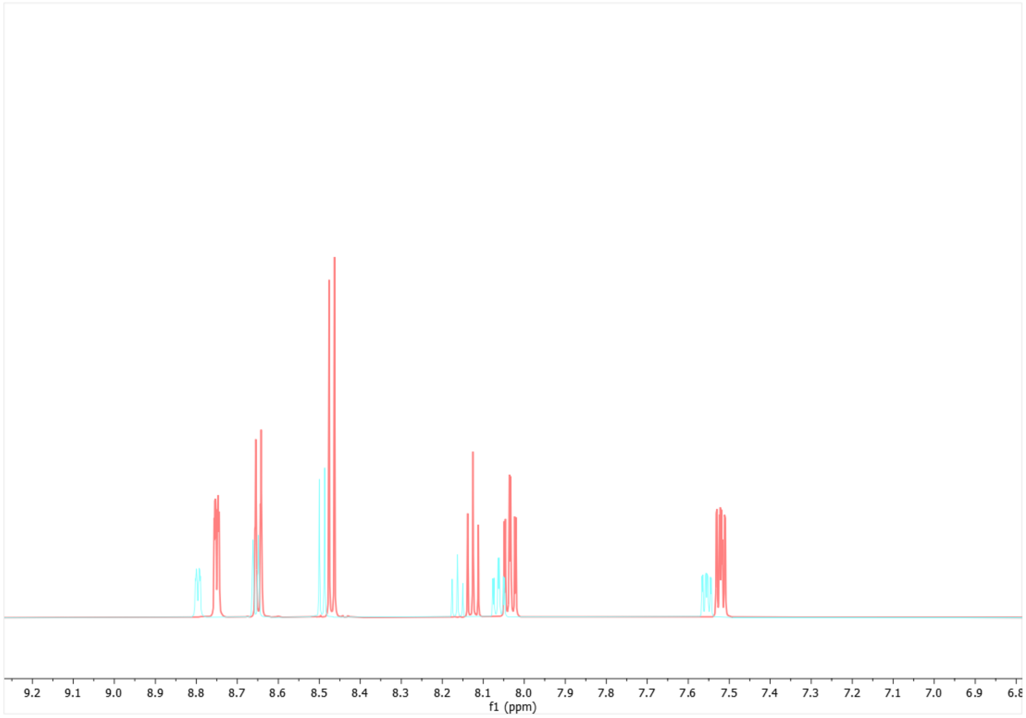

Fig. S3. The superimposed ^{1}H NMR spectra of tpy (red) and (tpy)$PbCl_2$ (cyan) in DMSO-d_6.

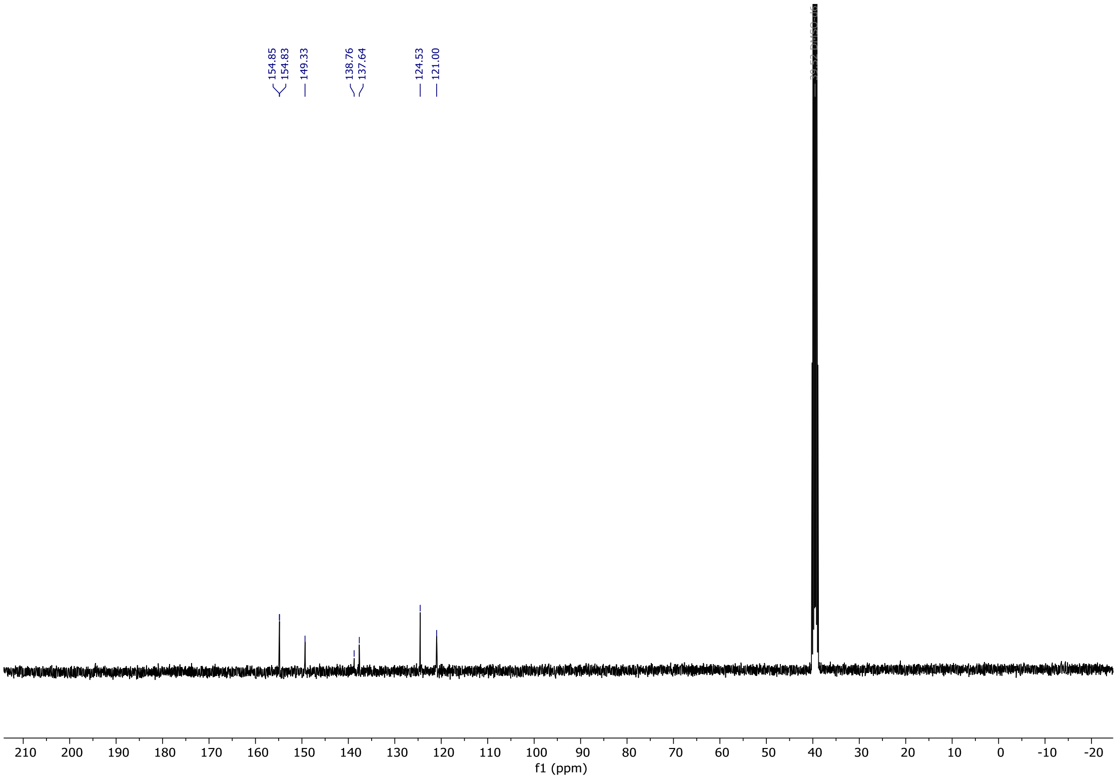

Fig. S4. The ^{13}C NMR spectra of (tpy)$PbCl_2$ in DMSO-d_6.

X-ray Crystallography. Data for **1** were collected on a Bruker X8 Kappa Apex II diffractometer, while data for $[Pb_2Cl_4(DMSO)_3]_n$ were collected on a Bruker D8 VENTURE diffractometer, both using Mo Kα radiation. Crystal data, data collection and refinement parameters are summarized in Table S1.

The structure for **1** was solved using a dual-space method and standard difference map techniques, and was refined by full-matrix least-squares procedures on F^2 with SHELXTL (Version 2017/1).[1] The structure for $[Pb_2Cl_4(DMSO)_3]_n$ was solved using direct methods and standard difference map techniques, and was also refined by full-matrix least-squares procedures on F^2 with SHELXTL (Version 2018/3).[1] All hydrogen atoms were placed in calculated positions and refined with a riding model [$U_{iso}(H) = 1.2–1.5U_{eq}(C)$]. A twin law was required to acount for pseudosymmetry in **1**, which is broken by mixed orientation of the $SiMe_3$ groups. Cambridge Crystallographic Data Centre (CCDC) No. 2171871 and 2179452 contain the supplementary crystallographic data for this paper. These data can be obtained free of charge via http://www.ccdc.cam.ac.uk/conts/retrieving.html, or from the Cambridge Crystallographic Data Centre, 12 Union Road, Cambridge CB2 1EZ, UK; fax: (+44) 1223-336-033; or e-mail: deposit@ccdc.cam.ac.uk.

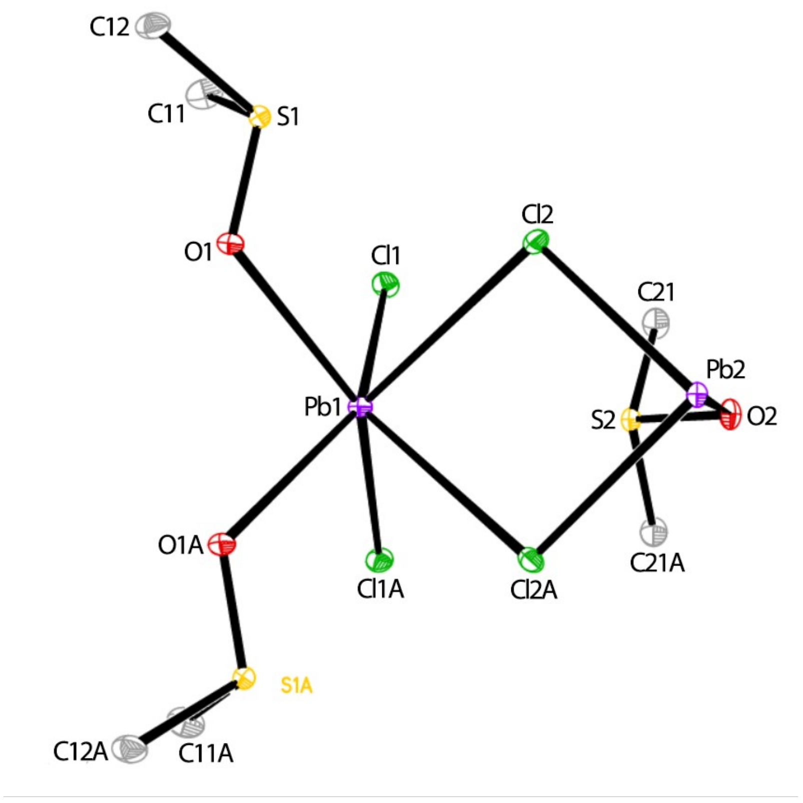

(a)

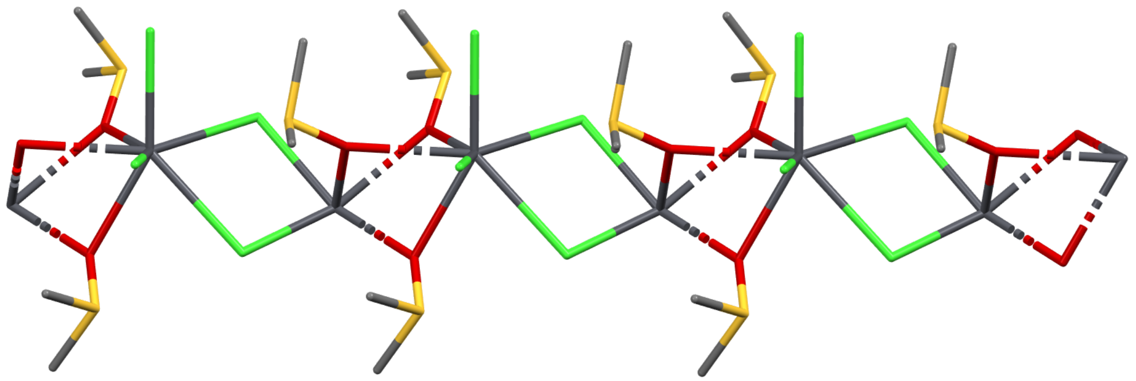

(b)

Fig. S5. The ORTEP diagram of $[Pb_2Cl_4(DMSO)_3]_n$ displays thermal ellipsoids drawn at the 30% probability level (a) and the partial extended structure of the polymer observed in the crystal (b).

Table S1. Crystal, intensity collection, and refinement data.

	**1**	**$[Pb_2Cl_4(DMSO)_3]_n$**
lattice	Trigonal	Orthorhombic
formula	$C_{24}H_{66}Pb_2Si_6$	$C_6H_{18}Cl_4O_3Pb_2S_3$
formula weight	937.68	790.56
space group	*P3*	*Pnma*
a/Å	10.6008(2)	13.9407(5)
b/Å	10.6008(2)	17.4187(6)
c/Å	9.9986(2)	7.3245(2)
α/°	90	90
β/°	90	90
γ/°	120	90
V/Å^3	973.08(4)	1778.60(10)
Z	1	4
temperature (K)	130(2)	130(2)
radiation (λ, Å)	0.71073	0.71073
ρ (calcd.) g cm^{-3}	1.600	2.952
μ (Mo Kα), mm^{-1}	8.836	19.855
θ max, deg.	36.261	36.333
no. of data collected	29256	93502
no. of data	6315	4427
no. of parameters	104	109
R_1 [$I > 2\sigma(I)$]	0.0179	0.0151
wR_2 [$I > 2\sigma(I)$]	0.0460	0.0319
R_1 [all data]	0.0191	0.0157
wR_2 [all data]	0.0465	0.0321
GOF	1.071	1.228
R_{int}	0.0271	0.0599

Synthetic details and characterization data

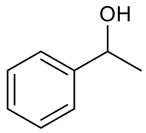

2a:[2] In a glovebox under nitrogen atmosphere, complex **1** (2.35 mg, 0.25 mmol, 0.25 mol%) was placed in a 3.8 mL glass vial equipped with a stir bar. Acetophenone (120.0 mg, 1.0 mmol) and pinacolborane (140.8 mg, 1.1 mmol) were then added. The reaction mixture was allowed to stir at room temperature for 16 h. At completion of the reaction, the reaction was exposed to the air and the solvent was evaporated. The product was purified through flash column chromatography with SiO_2 using ethyl acetate/hexane (1:10, v/v) as eluent to give colorless oil. Yield: 115.0 mg (94%). ^{1}H NMR (500 MHz, $CDCl_3$) δ 7.24 – 7.14 (m, 4H), 7.10 (m, 1H), 4.68 (q, J = 6.5 Hz, 1H), 2.16 (s, 1H), 1.30 (d, J = 6.5 Hz, 3H) ppm; ^{13}C NMR (126 MHz, $CDCl_3$) δ 145.9, 128.5, 127.5, 125.5, 70.4, 25.2 ppm.

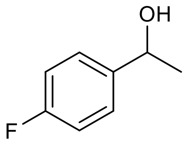

2b:[2] In a glovebox under nitrogen atmosphere, complex **1** (2.35 mg, 0.25 mmol, 0.25 mol%) was placed in a 3.8 mL glass vial equipped with a stir bar. 4'-Fluoroacetophenone (138.0 mg, 1.0 mmol) and pinacolborane (140.8 mg, 1.1 mmol) were then added. The reaction mixture was allowed to stir at room temperature for 16 h. At completion of the reaction, the reaction was exposed to the air and the solvent was evaporated. The product was purified through flash column chromatography with SiO_2 using ethyl acetate/hexane (1:10, v/v) as eluent to give colorless oil. Yield: 126 mg (90%). ^{1}H NMR (600 MHz, $CDCl_3$) δ 7.34 (ddd, J = 8.9, 5.4, 0.7 Hz, 2H), 7.03 (t, J = 8.7 Hz, 2H), 4.89 (q, J = 6.5 Hz, 1H), 1.79 (s, 1H), 1.48 (d, J = 6.5 Hz, 3H) ppm; ^{13}C NMR (151 MHz, $CDCl_3$) δ 162.30 (d, J = 245.3 Hz), 141.68 (d, J = 3.2 Hz), 127.19 (d, J = 8.2 Hz), 115.41 (d, J = 21.1 Hz), 69.9, 25.4 ppm.

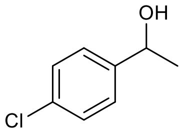

2c:[2] In a glovebox under nitrogen atmosphere, complex **1** (2.35 mg, 0.25 mmol, 0.25 mol%) was placed in a 3.8 mL glass vial equipped with a stir bar. 2'-Chloroacetophenone (154.0 mg, 1.0 mmol) and pinacolborane (140.8 mg, 1.1 mmol) were then added. The reaction mixture was allowed to stir at room temperature for 16 h. At completion of the reaction, the reaction was exposed to the air and the solvent was evaporated. The product was purified through flash column chromatography with SiO_2 using ethyl acetate/hexane (1:10, v/v) as eluent to give colorless oil. Yield: 138 mg (88%). ^{1}H NMR (600 MHz, $CDCl_3$) δ 7.46 (d, J = 8.4 Hz, 2H), 7.26 – 7.19 (m, 2H), 4.84 (d, J = 6.5 Hz, 1H), 2.00 (s, 1H), 1.46 (d, J = 6.6 Hz, 3H) ppm; ^{13}C NMR (151 MHz, $CDCl_3$) δ 144.9, 131.7, 127.3, 121.3, 69.9, 25.3 ppm.

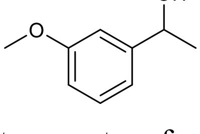

2d:[2] In a glovebox under nitrogen atmosphere, complex **1** (2.35 mg, 0.25 mmol, 0.25 mol%) was placed in a 3.8 mL glass vial equipped with a stir bar. 3'-Methoxyacetophenone (150.0 mg, 1.0 mmol) and pinacolborane (140.8 mg, 1.1 mmol) were then added. The reaction mixture was allowed to stir at room temperature for 16 h. At completion of the reaction, the reaction was exposed to the air and the solvent was evaporated. The product was purified through flash column chromatography with SiO_2 using ethyl acetate/hexane (1:10, v/v) as eluent to give colorless oil. Yield: 142 mg (93%). ^{1}H NMR (600 MHz, $CDCl_3$) δ 7.27 (t, J = 8.1 Hz, 1H), 6.99 – 6.91 (m, 2H), 6.82 (ddd, J = 8.2, 2.6, 1.1 Hz, 1H), 4.87 (q, J = 6.5 Hz, 1H), 3.82 (s, 3H), 2.08 (s, 1H), 1.49 (d, J = 6.6 Hz, 3H)

ppm; ^{13}C NMR (151 MHz, $CDCl_3$) δ 159.9, 147.8, 129.6, 117.8, 113.0, 111.1, 70.4, 55.3, 25.2 ppm.

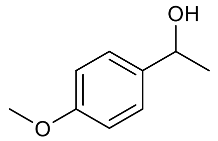

2e:[2] In a glovebox under nitrogen atmosphere, complex **1** (2.35 mg, 0.25 mmol, 0.25 mol%) was placed in a 3.8 mL glass vial equipped with a stir bar. 4'-Methoxyacetophenone (150.0 mg, 1.0 mmol) and pinacolborane (140.8 mg, 1.1 mmol) were then added. The reaction mixture was allowed to stir at room temperature for 16 h. At completion of the reaction, the reaction was exposed to the air and the solvent was evaporated. The product was purified through flash column chromatography with SiO_2 using ethyl acetate/hexane (1:10, v/v) as eluent to give colorless oil. Yield: 145 mg (95%). ^1H NMR (600 MHz, $CDCl_3$) δ 7.30 – 7.10 (m, 2H), 6.84 – 6.71 (m, 2H), 4.72 (q, J = 6.5 Hz, 1H), 3.77 – 3.60 (m, 3H), 2.22 (s, 1H), 1.36 (d, J = 6.4 Hz, 3H) ppm. ^{13}C NMR (151 MHz, $CDCl_3$) δ 158.9, 138.1, 126.7, 113.8, 69.9, 55.3, 25.1 ppm.

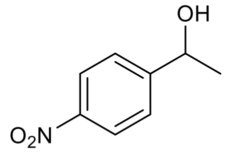

2f:[2] In a glovebox under nitrogen atmosphere, complex **1** (2.35 mg, 0.25 mmol, 0.25 mol%) was placed in a 3.8 mL glass vial equipped with a stir bar. 4'-Nitroacetophenone (165.0 mg, 1.0 mmol) and pinacolborane (140.8 mg, 1.1 mmol) were then added. The reaction mixture was allowed to stir at room temperature for 16 h. At completion of the reaction, the reaction was exposed to the air and the solvent was evaporated. The product was purified through flash column chromatography with SiO_2 using ethyl acetate/hexane (1:10, v/v) as eluent to give yellowish oil. Yield: 137 mg (82%). ^1H NMR (600 MHz, $CDCl_3$) δ 8.18 – 8.07 (m, 2H), 7.55 – 7.43 (m, 2H), 4.99 (d, J = 6.6 Hz, 1H), 2.45 (s, 1H), 1.49 (d, J = 6.6 Hz, 3H) ppm; ^{13}C NMR (151 MHz, $CDCl_3$) δ 153.3, 147.2, 126.2, 123.8, 69.5, 25.5 ppm.

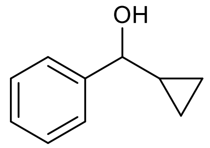

2g:[2] In a glovebox under nitrogen atmosphere, complex **1** (2.35 mg, 0.25 mmol, 0.25 mol%) was placed in a 3.8 mL glass vial equipped with a stir bar. Cyclopropyl phenyl ketone (146.0 mg, 1.0 mmol) and pinacolborane (140.8 mg, 1.1 mmol) were then added. The reaction mixture was allowed to stir at room temperature for 16 h. At completion of the reaction, the reaction was exposed to the air and the solvent was evaporated. The product was purified through flash column chromatography with SiO_2 using ethyl acetate/hexane (1:10, v/v) as eluent to give colorless oil. Yield: 126 mg (85%). ^1H NMR (500 MHz, $CDCl_3$) δ 7.48 – 7.40 (m, 2H), 7.37 (dd, J = 8.5, 6.7 Hz, 2H), 7.33 – 7.27 (m, 1H), 4.00 (d, J = 8.2 Hz, 1H), 2.27 (s, 1H), 1.22 (dt, J = 8.1, 4.9 Hz, 1H), 0.69 – 0.59 (m, 1H), 0.59 – 0.51 (m, 1H), 0.47 (dq, J = 9.8, 5.0 Hz, 1H), 0.38 (dt, J = 9.6, 4.8 Hz, 1H) ppm; ^{13}C NMR (126 MHz, $CDCl_3$) δ 144.0, 128.4, 127.6, 126.1, 78.5, 19.2, 3.7, 2.9 ppm.

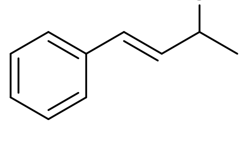

2h:[2] In a glovebox under nitrogen atmosphere, complex **1** (2.35 mg, 0.25 mmol, 0.25 mol%) was placed in a 3.8 mL glass vial equipped with a stir bar. 4-Phenylbut-3-en-2-one (146.0 mg, 1.0 mmol) and pinacolborane (140.8 mg, 1.1 mmol) were then added. The reaction mixture was allowed to stir at room temperature for 16 h. At completion of the reaction, the reaction was exposed to the air and the solvent was evaporated. The product was purified through flash column chromatography with SiO_2 using ethyl acetate/hexane (1:10, v/v) as eluent to give colorless oil. Yield: 129 mg (87%). ^1H NMR (600 MHz, $CDCl_3$) δ 7.45 – 7.31 (m, 4H), 7.29 –

7.23 (m, 1H), 6.60 (d, J = 15.8 Hz, 1H), 6.29 (dd, J = 15.9, 6.3 Hz, 1H), 4.52 (t, J = 6.4 Hz, 1H), 1.72 (s, 1H), 1.40 (d, J = 6.4 Hz, 3H) ppm; ^{13}C NMR (151 MHz, $CDCl_3$) δ 136.9, 133.9, 129.6, 128.8, 127.8, 126.7, 69.1, 23.6 ppm.

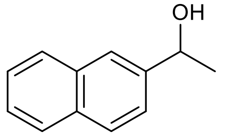

2i:[3] In a glovebox under nitrogen atmosphere, complex **1** (2.35 mg, 0.25 mmol, 0.25 mol%) was placed in a 3.8 mL glass vial equipped with a stir bar. 2-Acetylnaphthalene (170.0 mg, 1.0 mmol) and pinacolborane (140.8 mg, 1.1 mmol) were then added. The reaction mixture was allowed to stir at room temperature for 16 h. At completion of the reaction, the reaction was exposed to the air and the solvent was evaporated. The product was purified through flash column chromatography with SiO_2 using ethyl acetate/hexane (1:10, v/v) as eluent to give a white solid. Yield: 146 mg (85%). ^{1}H NMR (600 MHz, $CDCl_3$) δ 7.84 (d, J = 8.3 Hz, 3H), 7.80 (s, 1H), 7.55 – 7.42 (m, 3H), 5.05 (d, J = 6.4 Hz, 1H), 2.11 (s, 1H), 1.59 (d, J = 6.4 Hz, 3H) ppm; ^{13}C NMR (151 MHz, $CDCl_3$) δ 143.3, 133.5, 133.1, 128.4, 128.1, 127.8, 126.3, 125.9, 123.95, 123.92, 70.6, 25.2.

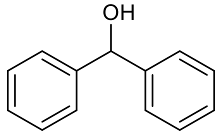

2j:[2] In a glovebox under nitrogen atmosphere, complex **1** (2.35 mg, 0.25 mmol, 0.25 mol%) was placed in a 3.8 mL glass vial equipped with a stir bar. Benzophenone (182.1 mg, 1.0 mmol) and pinacolborane (140.8 mg, 1.1 mmol) were then added. The reaction mixture was allowed to stir at room temperature for 16 h. At completion of the reaction, the reaction was exposed to the air and the solvent was evaporated. The product was purified through flash column chromatography with SiO_2 using ethyl acetate/hexane (1:10, v/v) as eluent to give a white solid. Yield: 158 mg (86%). ^{1}H NMR (600 MHz, $CDCl_3$) δ 7.48 – 7.33 (m, 8H), 7.32 – 7.26 (m, 2H), 5.88 (s, 1H), 2.22 (s, 1H) ppm; ^{13}C NMR (151 MHz, $CDCl_3$) δ 144.0, 128.7, 127.7, 126.7, 76.5 ppm.

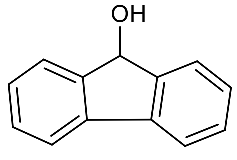

2k:[4] In a glovebox under nitrogen atmosphere, complex **1** (2.35 mg, 0.25 mmol, 0.25 mol%) was placed in a 3.8 mL glass vial equipped with a stir bar. Fluorenone (180.0 mg, 1.0 mmol) and pinacolborane (140.8 mg, 1.1 mmol) were then added. The reaction mixture was allowed to stir at room temperature for 16 h. At completion of the reaction, the reaction was exposed to the air and the solvent was evaporated. The product was purified through flash column chromatography with SiO_2 using ethyl acetate/hexane (1:10, v/v) as eluent to give a white solid. Yield: 164 mg (90%). ^{1}H NMR (600 MHz, CD_3OD) δ 7.71 – 7.63 (m, 2H), 7.63 – 7.56 (m, 2H), 7.36 (td, J = 7.5, 1.5 Hz, 2H), 7.30 (td, J = 7.4, 1.4 Hz, 2H), 5.49 (s, 1H), 4.94 (s, 1H) ^{1}H NMR (600 MHz, $CDCl_3$) δ 7.67 – 7.58 (m, 4H), 7.38 (td, J = 7.5, 1.2 Hz, 2H), 7.31 (td, J = 7.4, 1.2 Hz, 2H), 5.52 (s, 1H), 2.04 – 1.97 (m, 1H) ppm; ^{13}C NMR (151 MHz, $CDCl_3$) δ 145.8, 140.1, 129.2, 127.9, 125.2, 120.1, 75.3 ppm.

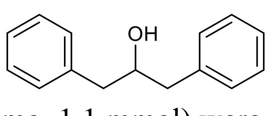

2l:[4] In a glovebox under nitrogen atmosphere, complex **1** (2.35 mg, 0.25 mmol, 0.25 mol%) was placed in a 3.8 mL glass vial equipped with a stir bar. 1,3-Diphenylacetone (210.0 mg, 1.0 mmol) and pinacolborane (140.8 mg, 1.1 mmol) were then added. The reaction mixture was allowed to stir at room temperature for 16 h. At completion of the reaction, the reaction was exposed to the air and the solvent was evaporated. The product was purified through flash column chromatography with SiO_2 using ethyl acetate/hexane (1:10, v/v) as eluent to give colorless oil. Yield: 194 mg (92%). ^{1}H NMR (600 MHz, $CDCl_3$) δ 7.26 (dd, J = 8.9, 6.6 Hz, 4H), 7.18 (dd, J = 8.7, 6.5 Hz, 6H), 3.99 (tt, J =

8.1, 4.7 Hz, 1H), 2.79 (dd, J = 13.7, 4.8 Hz, 2H), 2.70 (dd, J = 13.7, 8.1 Hz, 2H), 1.69 (s, 1H) ppm; ^{13}C NMR (151 MHz, $CDCl_3$) δ 138.6, 129.5, 128.6, 126.5, 73.6, 43.5 ppm.

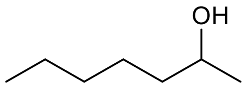

2m:[4] In a glovebox under nitrogen atmosphere, complex **1** (2.35 mg, 0.25 mmol, 0.25 mol%) was placed in a 3.8 mL glass vial equipped with a stir bar. 2-Heptanone (114.0 mg, 1.0 mmol) and pinacolborane (140.8 mg, 1.1 mmol) were then added. The reaction mixture was allowed to stir at room temperature for 16 h. At completion of the reaction, the reaction was exposed to the air and the solvent was evaporated. The product was purified through flash column chromatography with SiO_2 using ethyl acetate/hexane (1:30, v/v) as eluent to give colorless oil. Yield: 90 mg (78%). ^{1}H NMR (600 MHz, $CDCl_3$) δ 3.83 – 3.74 (m, 1H), 1.49 – 1.37 (m, 4H), 1.35 – 1.26 (m, 5H), 1.18 (d, J = 6.2 Hz, 3H), 0.91 – 0.86 (m, 3H) ppm; ^{13}C NMR (151 MHz, $CDCl_3$) δ 68.3, 39.5, 32.0, 25.6, 23.6, 22.8, 14.1 ppm.

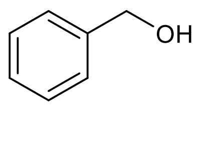

3a:[4] In a glovebox under nitrogen atmosphere, complex **1** (2.35 mg, 0.25 mmol, 0.25 mol%) was placed in a 3.8 mL glass vial equipped with a stir bar. Benzaldehyde (106.0 mg, 1.0 mmol) and pinacolborane (140.8 mg, 1.1 mmol) were then added. The reaction mixture was allowed to stir at room temperature for 16 h. At completion of the reaction, the reaction was exposed to the air and the solvent was evaporated. The product was purified through flash column chromatography with SiO_2 using ethyl acetate/hexane (1:10, v/v) as eluent to give colorless oil. Yield: 101 mg (93%). ^{1}H NMR (500 MHz, $CDCl_3$) δ 7.25 (m, 4H), 7.19 (m, 1H), 4.54 (s, 2H), 2.90 (s, 1H) ppm; ^{13}C NMR (126 MHz, $CDCl_3$) δ 140.9, 128.6, 127.7, 127.1, 65.3 pm.

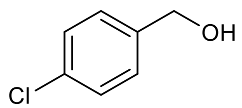

3b:[2] In a glovebox under nitrogen atmosphere, complex **1** (2.35 mg, 0.25 mmol, 0.25 mol%) was placed in a 3.8 mL glass vial equipped with a stir bar. 4-Chlorobenzaldehyde (140.5 mg, 1.0 mmol) and pinacolborane (140.8 mg, 1.1 mmol) were then added. The reaction mixture was allowed to stir at room temperature for 16 h. At completion of the reaction, the reaction was exposed to the air and the solvent was evaporated. The product was purified through flash column chromatography with SiO_2 using ethyl acetate/hexane (1:10, v/v) as eluent to give colorless oil. Yield: 128 mg (90%). ^{1}H NMR (600 MHz, $CDCl_3$) δ 7.27 – 7.16 (m, 2H), 7.13 (d, J = 8.6 Hz, 2H), 4.47 (d, J = 1.4 Hz, 2H), 2.62 (s, 1H) ppm; ^{13}C NMR (151 MHz, $CDCl_3$) δ 139.3, 133.4, 128.7, 128.3, 64.4 ppm.

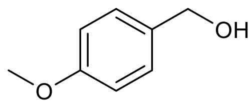

3c:[2] In a glovebox under nitrogen atmosphere, complex **1** (2.35 mg, 0.25 mmol, 0.25 mol%) was placed in a 3.8 mL glass vial equipped with a stir bar. 4-Methoxybenzaldehyde (140 mg, 1.0 mmol) and pinacolborane (140.8 mg, 1.1 mmol) were then added. The reaction mixture was allowed to stir at room temperature for 16 h. At completion of the reaction, the reaction was exposed to the air and the solvent was evaporated. The product was purified through flash column chromatography with SiO_2 using ethyl acetate/hexane (1:10, v/v) as eluent to give colorless oil. Yield: 136.0 mg (96%). ^{1}H NMR (600 MHz, $CDCl_3$) δ 7.28 – 7.19 (m, 2H), 6.89 – 6.82 (m, 2H), 4.53 (s, 2H), 3.76 (d, J = 3.9 Hz, 3H), 2.42 (s, 1H) ppm; ^{13}C NMR (151 MHz, $CDCl_3$) δ 159.2, 133.3, 128.7, 114.0, 64.8, 55.3 ppm.

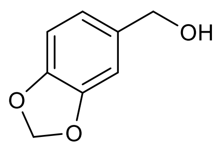

3d:[4] In a glovebox under nitrogen atmosphere, complex **1** (2.35 mg, 0.25 mmol, 0.25 mol%) was placed in a 3.8 mL glass vial equipped with a stir bar. 1,3-Benzodioxole-5-carboxaldehyde (121.0 mg, 1.0 mmol) and pinacolborane (150.0 mg, 1.1 mmol) were then added. The reaction mixture was allowed to stir at room temperature for 16 h. At completion of the reaction, the reaction was exposed to the air and the solvent was evaporated. The product was purified through flash column chromatography with SiO_2 using ethyl acetate/hexane (1:10, v/v) as eluent to give colorless oil. Yield: 135 mg (89%). ^{1}H NMR (600 MHz, $CDCl_3$) δ 6.81 (d, J = 1.7 Hz, 1H), 6.79 – 6.70 (m, 2H), 5.91 (s, 2H), 4.50 (s, 2H), 2.47 (d, J = 2.2 Hz, 1H) ppm; ^{13}C NMR (151 MHz, $CDCl_3$) δ 147.8, 147.1, 135.0, 120.5, 108.2, 107.9, 101.0, 65.1 ppm.

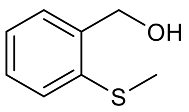

3e:[3] In a glovebox under nitrogen atmosphere, complex **1** (2.35 mg, 0.25 mmol, 0.25 mol%) was placed in a 3.8 mL glass vial equipped with a stir bar. 2-Methylthiobenzaldehyde (152.0 mg, 1.0 mmol) and pinacolborane (140.8 mg, 1.1 mmol) were then added. The reaction mixture was allowed to stir at room temperature for 16 h. At completion of the reaction, the reaction was exposed to the air and the solvent was evaporated. The product was purified through flash column chromatography with SiO_2 using ethyl acetate/hexane (1:10, v/v) as eluent to give yellowish oil. Yield: 139 mg (90%). ^{1}H NMR (600 MHz, $CDCl_3$) δ 7.37 (dd, J = 7.6, 1.4 Hz, 1H), 7.33 – 7.23 (m, 2H), 7.18 (ddd, J = 7.5, 6.4, 2.3 Hz, 1H), 4.73 (d, J = 1.2 Hz, 2H), 2.66 (s, 1H), 2.47 (s, 3H) ppm; ^{13}C NMR (126 MHz, $CDCl_3$) δ 138.9, 136.5, 128.2, 127.8, 126.4, 125.4, 63.2, 16.0 ppm.

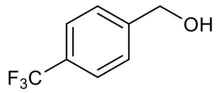

3f:[2] In a glovebox under nitrogen atmosphere, complex **1** (2.35 mg, 0.25 mmol, 0.25 mol%) was placed in a 3.8 mL glass vial equipped with a stir bar. 4′-Trifluoromethylbenzaldehyde (178.0 mg, 1.0 mmol) and pinacolborane (140.8 mg, 1.1 mmol) were then added. The reaction mixture was allowed to stir at room temperature for 16 h. At completion of the reaction, the reaction was exposed to the air and the solvent was evaporated. The product was purified through flash column chromatography with SiO_2 using ethyl acetate/hexane (1:10, v/v) as eluent to give yellowish oil. Yield: 164 mg (93%). ^{1}H NMR (600 MHz, $CDCl_3$) δ 7.58 (d, J = 8.3 Hz, 2H), 7.42 (d, J = 8.3 Hz, 2H), 4.69 (s, 2H), 2.68 (s, 1H) ppm; ^{13}C NMR (150 MHz, $CDCl_3$) δ 144.8, 129.9 (q, J = 32.4 Hz), 126.9, 125.5 (q, J = 3.8 Hz), 125.2, 123.4, 64.4 ppm.

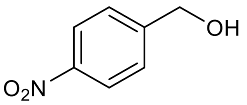

3g:[4] In a glovebox under nitrogen atmosphere, complex **1** (2.35 mg, 0.25 mmol, 0.25 mol%) was placed in a 3.8 mL glass vial equipped with a stir bar. 4′-Nitrobenzaldehyde (121.0 mg, 1.0 mmol) and pinacolborane (140.8 mg, 1.1 mmol) were then added. The reaction mixture was allowed to stir at room temperature for 16 h. At completion of the reaction, the reaction was exposed to the air and the solvent was evaporated. The product was purified through flash column chromatography with SiO_2 using ethyl acetate/hexane (1:10, v/v) as eluent to give yellowish oil. Yield: 129 mg (84%). ^{1}H NMR (600 MHz, $CDCl_3$) δ 8.22 – 8.11 (m, 2H), 7.55 – 7.46 (m, 2H), 4.81 (s, 2H), 2.31 (s, 1H) ppm; ^{13}C NMR (151 MHz, $CDCl_3$) δ 148.4, 147.4, 127.1, 123.8, 64.1 ppm.

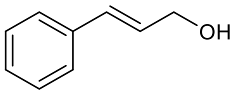

3h:[2] In a glovebox under nitrogen atmosphere, complex **1** (2.35 mg, 0.25 mmol, 0.25 mol%) was placed in a 3.8 mL glass vial equipped with a stir bar. *Trans*-cinnamaldehyde (132.0 mg, 1.0 mmol) and pinacolborane (140.8

mg, 1.1 mmol) were then added. The reaction mixture was allowed to stir at room temperature for 16 h. At completion of the reaction, the reaction was exposed to the air and the solvent was evaporated. The product was purified through flash column chromatography with SiO_2 using ethyl acetate/hexane (1:10, v/v) as eluent to give colorless oil. Yield: 123 mg (92%). ^{1}H NMR (600 MHz, $CDCl_3$) δ 7.29 (d, *J* = 7.9 Hz, 2H), 7.22 (t, *J* = 7.6 Hz, 2H), 7.15 (t, *J* = 7.3 Hz, 1H), 6.51 (d, *J* = 15.9 Hz, 1H), 6.32 – 6.19 (m, 1H), 4.21 (d, *J* = 6.1 Hz, 2H), 1.90 (s, 1H) ppm; ^{13}C NMR (151 MHz, $CDCl_3$) δ 136.8, 131.2, 128.69, 128.64, 127.8, 126.6, 63.7 ppm.

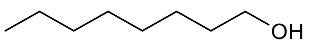

3i:[4] In a glovebox under nitrogen atmosphere, complex **1** (2.35 mg, 0.25 mmol, 0.25 mol%) was placed in a 3.8 mL glass vial equipped with a stir bar. 1-Octanal (128.0 mg, 1.0 mmol) and pinacolborane (140.8 mg, 1.1 mmol) were then added. The reaction mixture was allowed to stir at room temperature for 16 h. At completion of the reaction, the reaction was exposed to the air and the solvent was evaporated. The product was purified through flash column chromatography with SiO_2 using ethyl acetate/hexane (1:10, v/v) as eluent to give colorless oil. Yield: 117 mg (90%). ^{1}H NMR (500 MHz, $CDCl_3$) δ 3.61 (t, *J* = 6.9 Hz, 2H), 1.74 (s, 1H), 1.55 (t, *J* = 7.1 Hz, 2H), 1.40 – 1.17 (m, 10H), 0.92 – 0.82 (m, 3H) ppm; ^{13}C NMR (126 MHz, $CDCl_3$) δ 63.1, 32.9, 31.9, 29.5, 29.4, 25.9, 22.8, 14.2 ppm.

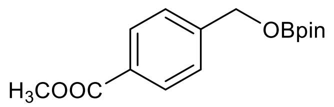

4: In a glovebox under nitrogen atmosphere, complex **1** (2.35 mg, 0.25 mmol, 0.25 mol%) was placed in a 3.8 mL glass vial equipped with a stir bar. Methyl 4-formylbenzoate (164.1 mg, 1.0 mmol) and pinacolborane (140.8 mg, 1.1 mmol) were then added. The reaction mixture was allowed to stir at room temperature for 16 h. At completion of the reaction, the reaction was exposed to the air and the solvent was evaporated. The product was purified through flash column chromatography with SiO_2 using ethyl acetate/hexane (1:10, v/v) as eluent to give yellowish oil. Yield: 274 mg (94%). ^{1}H NMR (500 MHz, $CDCl_3$) δ 7.93 (d, *J* = 7.7 Hz, 2H), 7.33 (d, *J* = 8.3 Hz, 2H), 4.91 (s, 2H), 3.83 (d, *J* = 2.4 Hz, 3H), 1.19 (d, *J* = 2.5 Hz, 13H) ppm; ^{13}C NMR (126 MHz, $CDCl_3$) δ 167.0, 144.4, 129.7, 129.1, 126.2, 83.2, 66.1, 52.1, 24.6 ppm. MS: 292 (cald. 292.1).

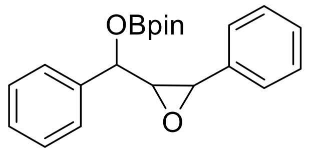

5: In a glovebox under nitrogen atmosphere, complex **1** (2.35 mg, 0.25 mmol, 0.25 mol%) was placed in a 3.8 mL glass vial equipped with a stir bar. Trans-2-benzoyl-3-phenyloxirane (224 mg, 1.0 mmol) and pinacolborane (140.8 mg, 1.1 mmol) were then added. THF (1 mL) was added to dissolve the substrate. The reaction mixture was allowed to stir at room temperature for 16 h. At completion of the reaction, white precipitate has formed and was filtered. The product was hydrolyzed in 1 M NaOH (10 mL) at room temperature for 1 h and extracted with diethyl ether and washed with 10% aq. $NaHCO_3$ and brine. The organic phase was separated and dried over Na_2SO_4. The solvent was removed to give the product as a white solid. Yield: 190 mg (84%). ^{1}H NMR (500 MHz, $CDCl_3$) δ 7.50 – 7.45 (m, 2H), 7.38 (dd, *J* = 8.2, 6.6 Hz, 2H), 7.35 – 7.31 (m, 3H), 7.30 – 7.25 (m, 3H), 5.30 (d, *J* = 3.6 Hz, 1H), 4.16 (d, *J* = 2.0 Hz, 1H), 3.23 (dd, *J* = 3.7, 2.0 Hz, 1H), 1.29 (s, 12H) ppm; ^{13}C NMR (126 MHz, $CDCl_3$) δ 139.1, 137.1, 128.6, 128.6, 128.3, 128.3, 126.5, 125.9, 83.4, 74.2, 64.7, 55.5, 24.8, 24.7 ppm. Anal. Calcd. for $C_{21}H_{25}BO_4$: C 71.61, H 7.15%. Found C 72.02, H 7.03%.

^{1}H NMR spectrum of **1** (600 MHz, toluene-d_8, * solvent residue Et_2O)

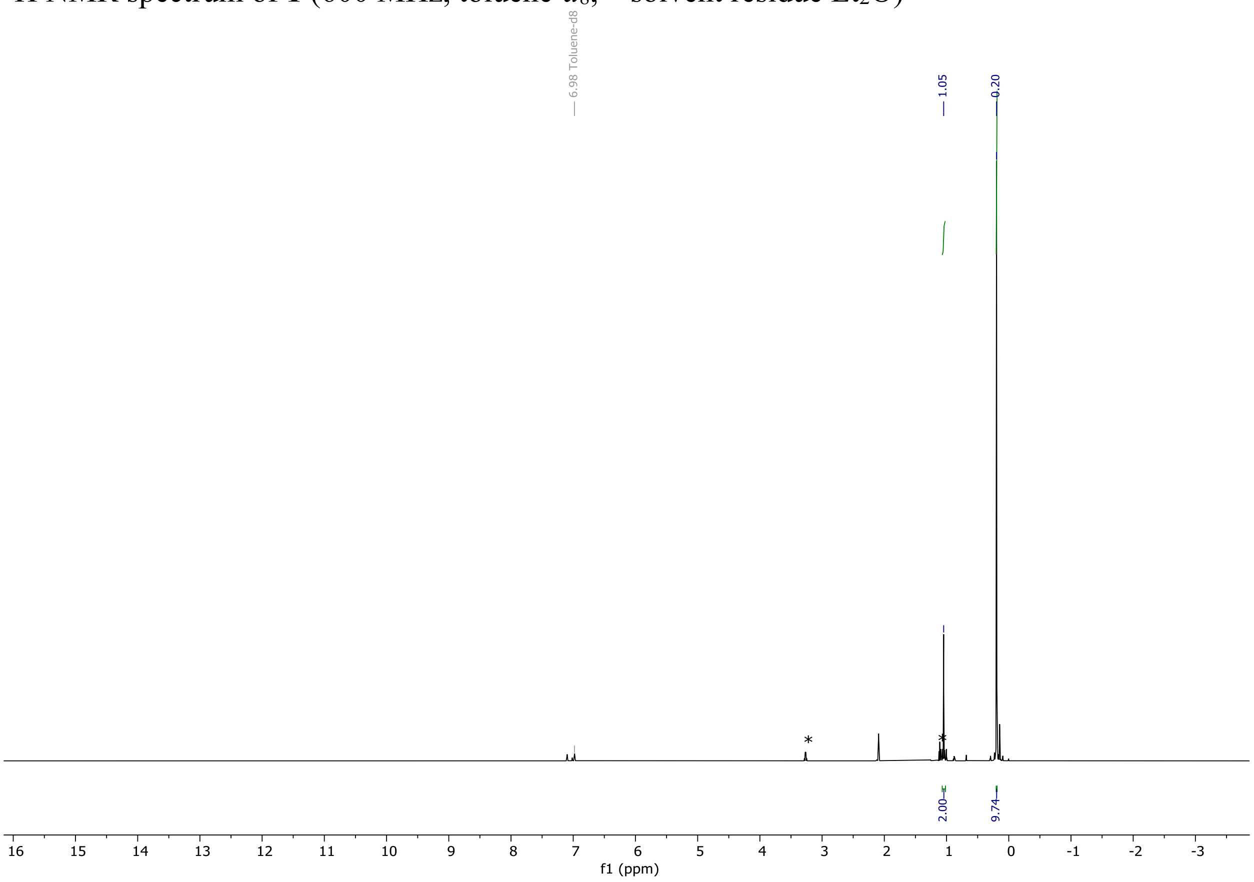

^{13}C NMR spectrum of **1** (126 MHz, toluene-d_8)

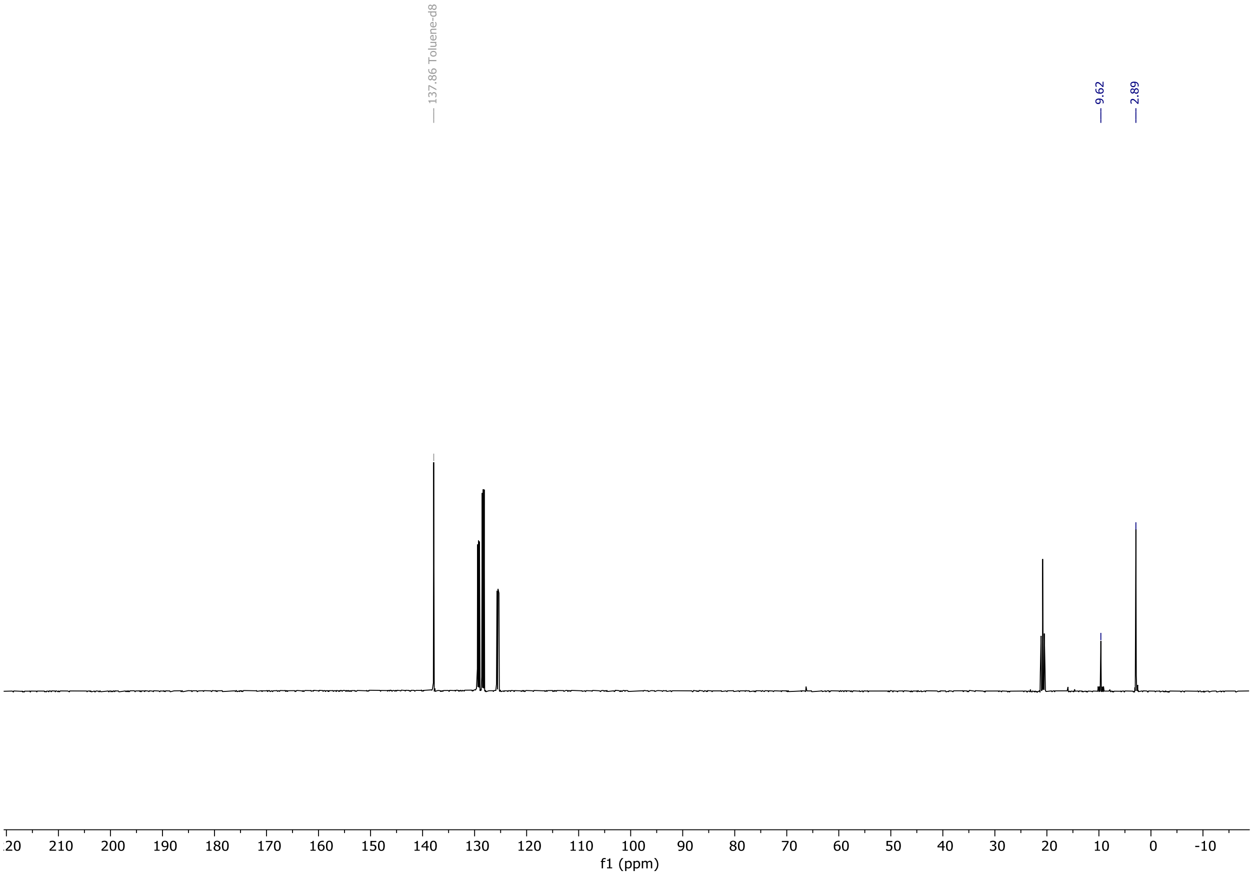

^{207}Pb NMR spectrum of **1** (84 MHz, toluene-d_8)

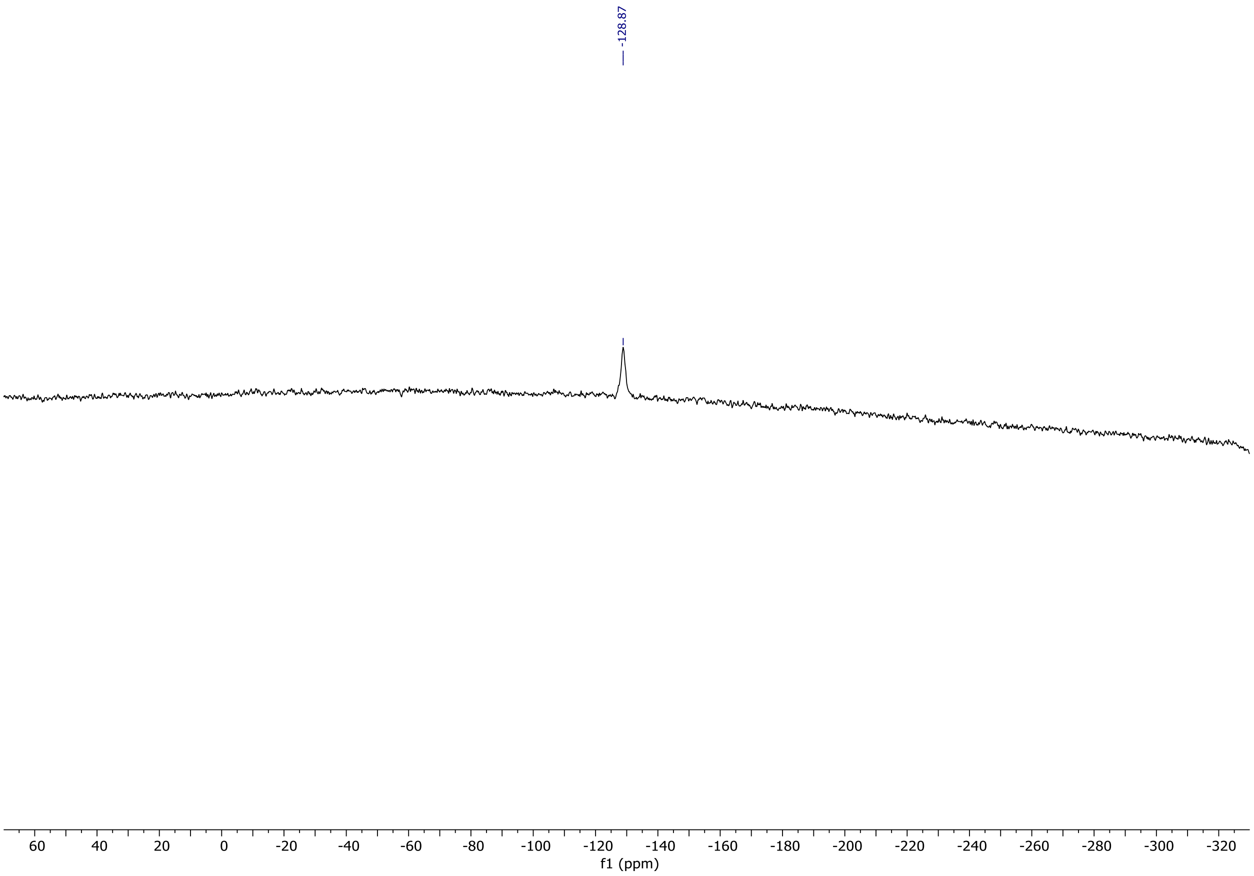

^{1}H NMR spectrum of **1** with added HBpin (5 eq.) (600 MHz, toluene-d_8)

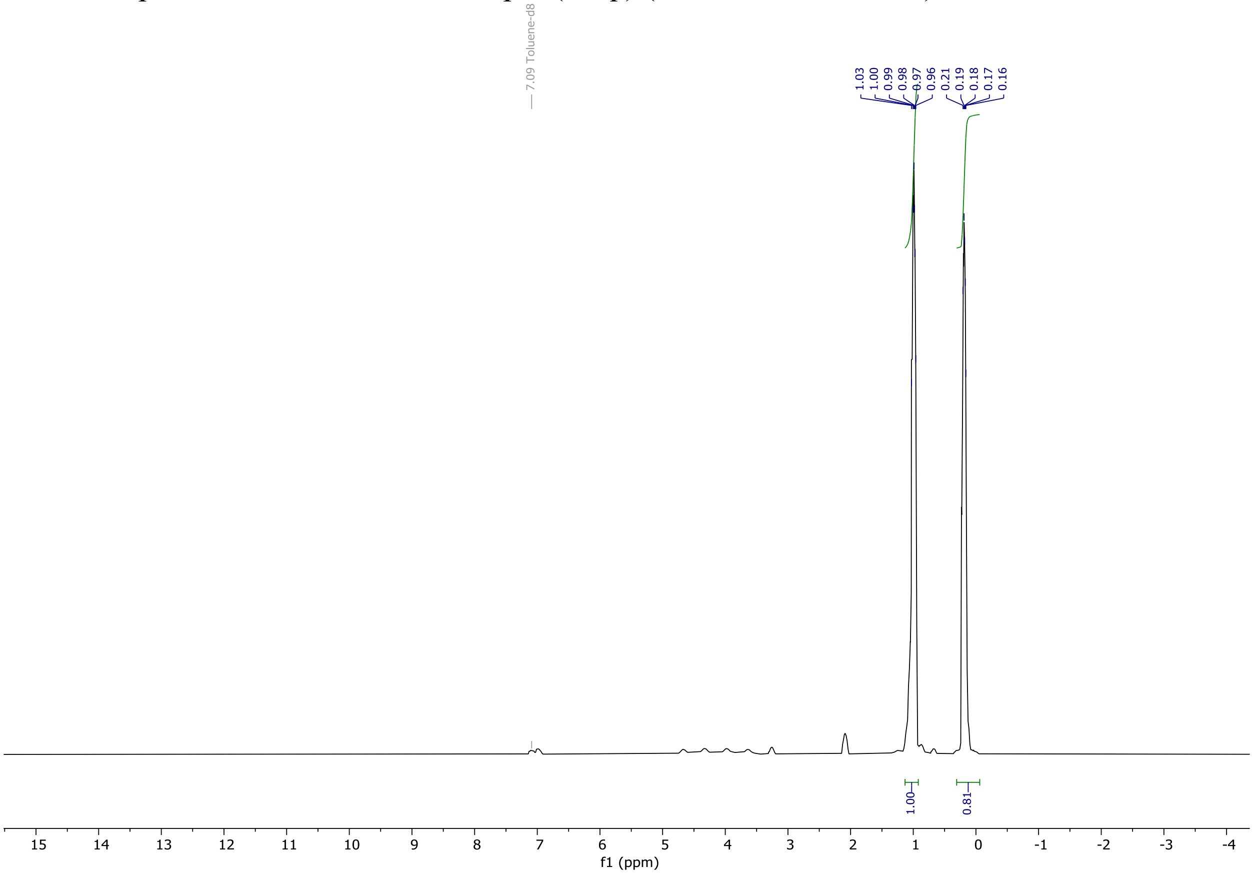

^{13}C NMR spectrum of **1** with added HBpin (5 eq.) (600 MHz, toluene-d_8)

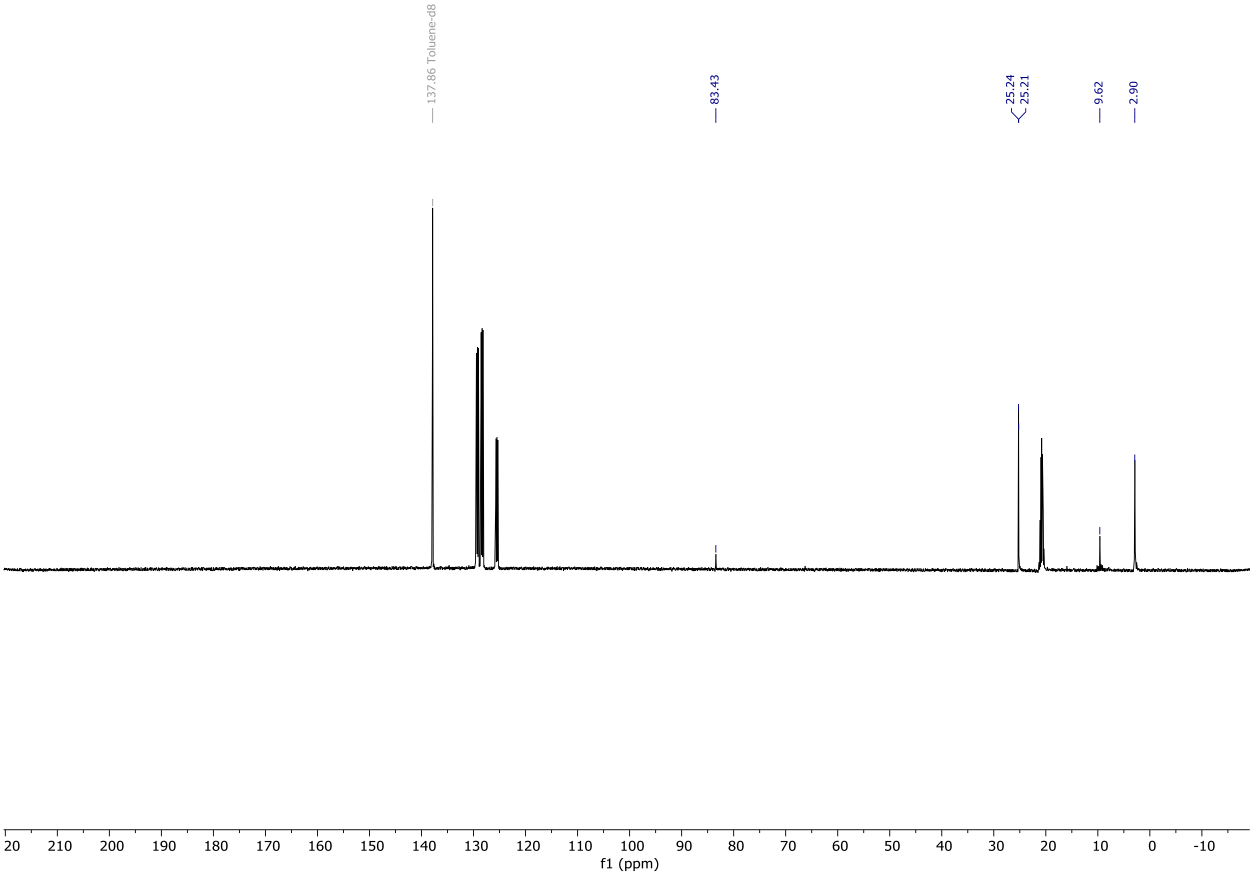

NMR spectra for isolated products:

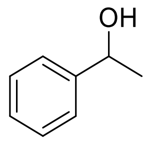

(**2a**)

^{1}H NMR (500 MHz, $CDCl_3$):

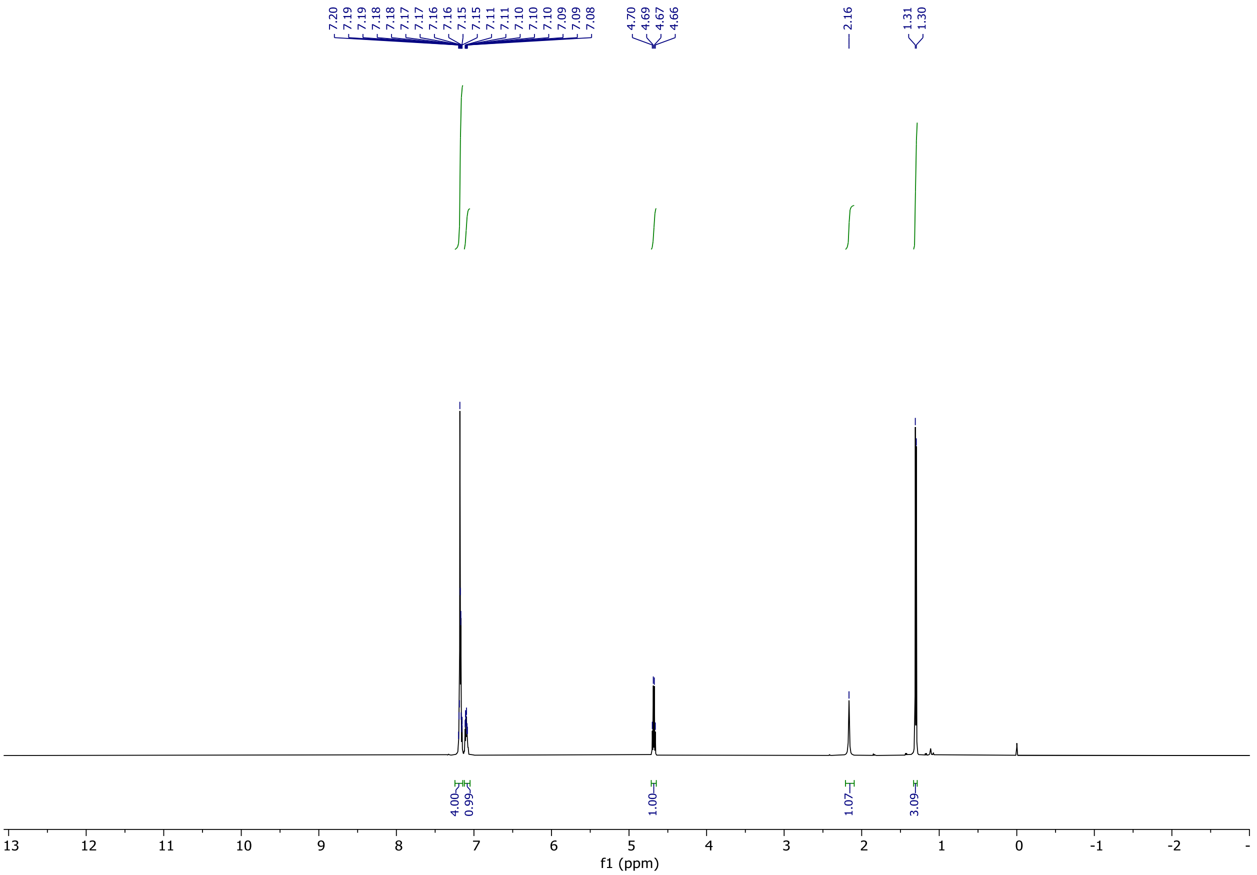

^{13}C NMR (126 MHz, $CDCl_3$):

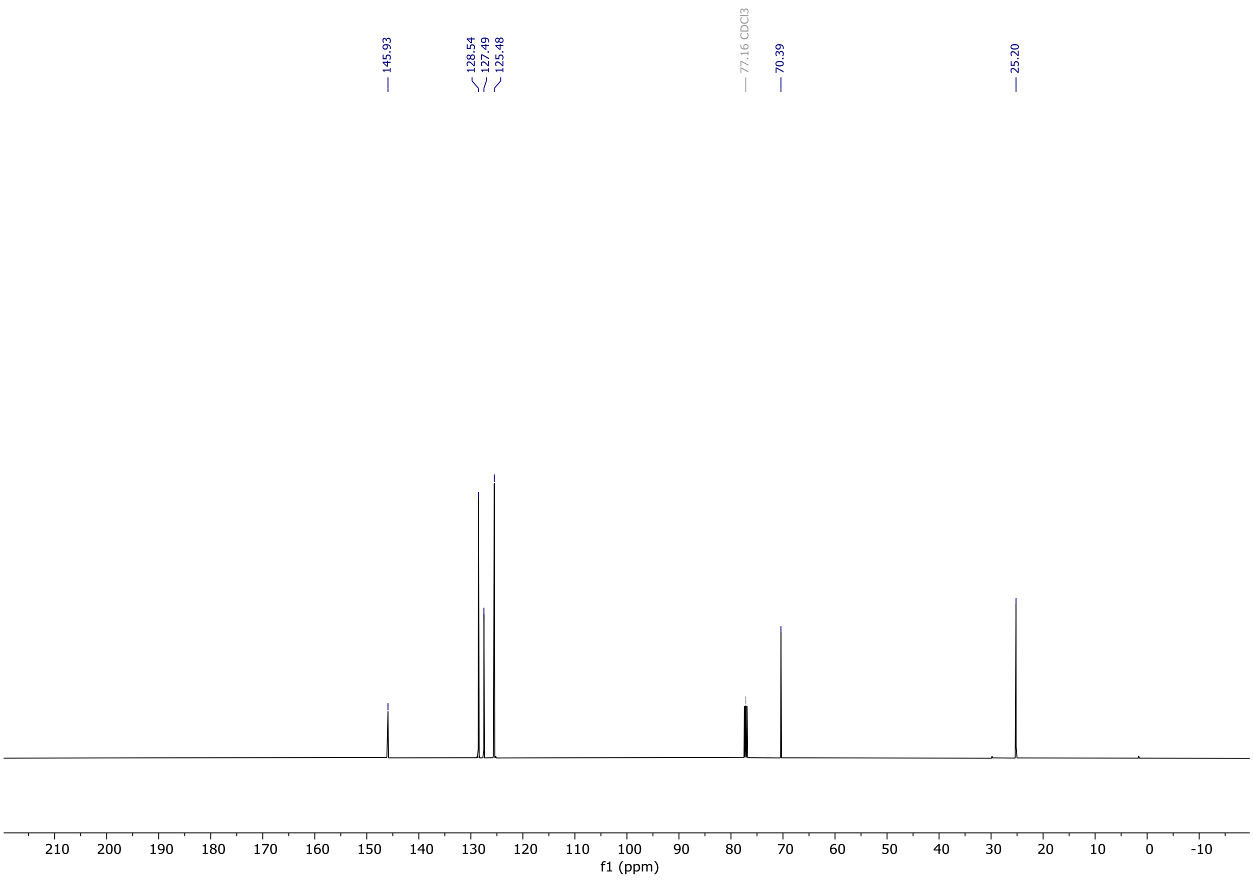

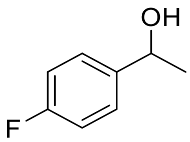

(**2b**)

^{1}H NMR (600 MHz, $CDCl_3$):

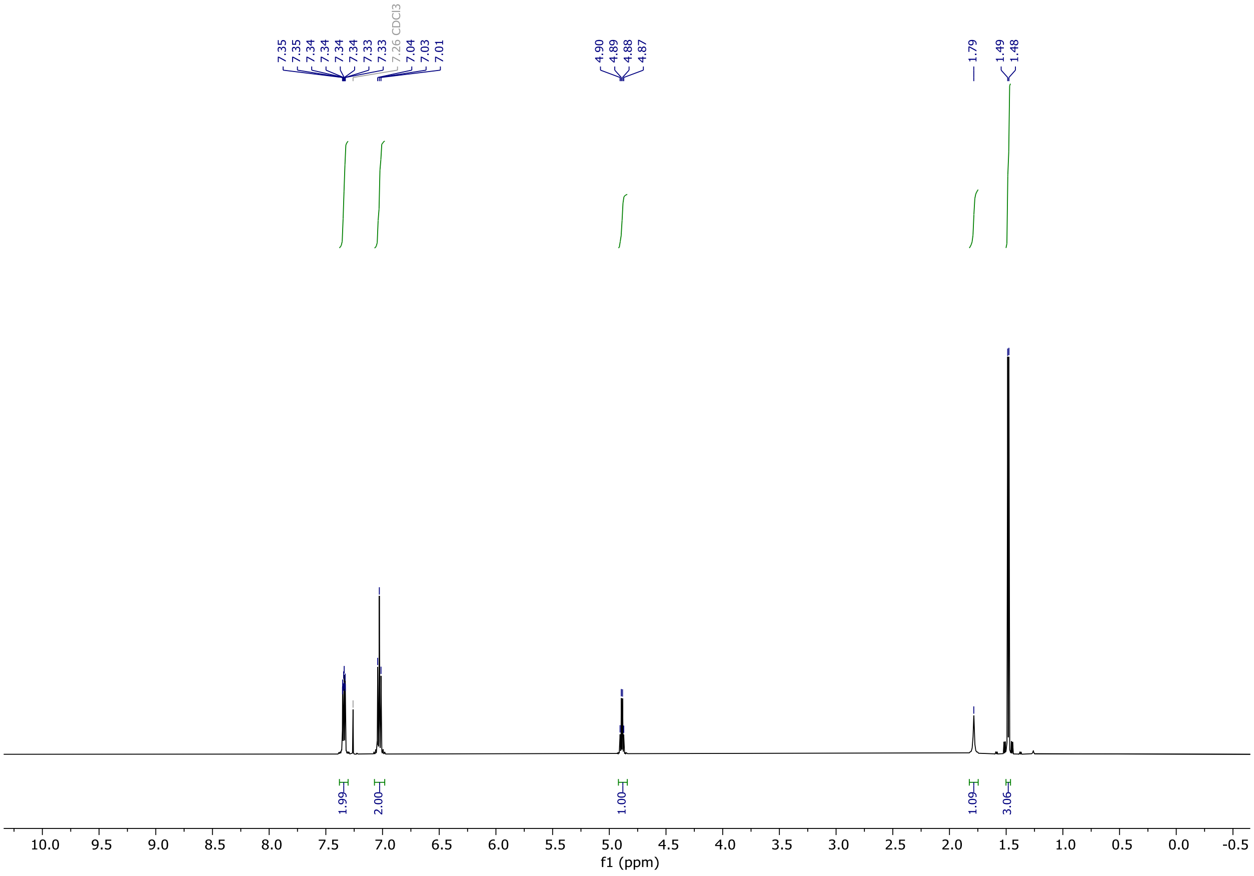

^{13}C NMR (151MHz, $CDCl_3$):

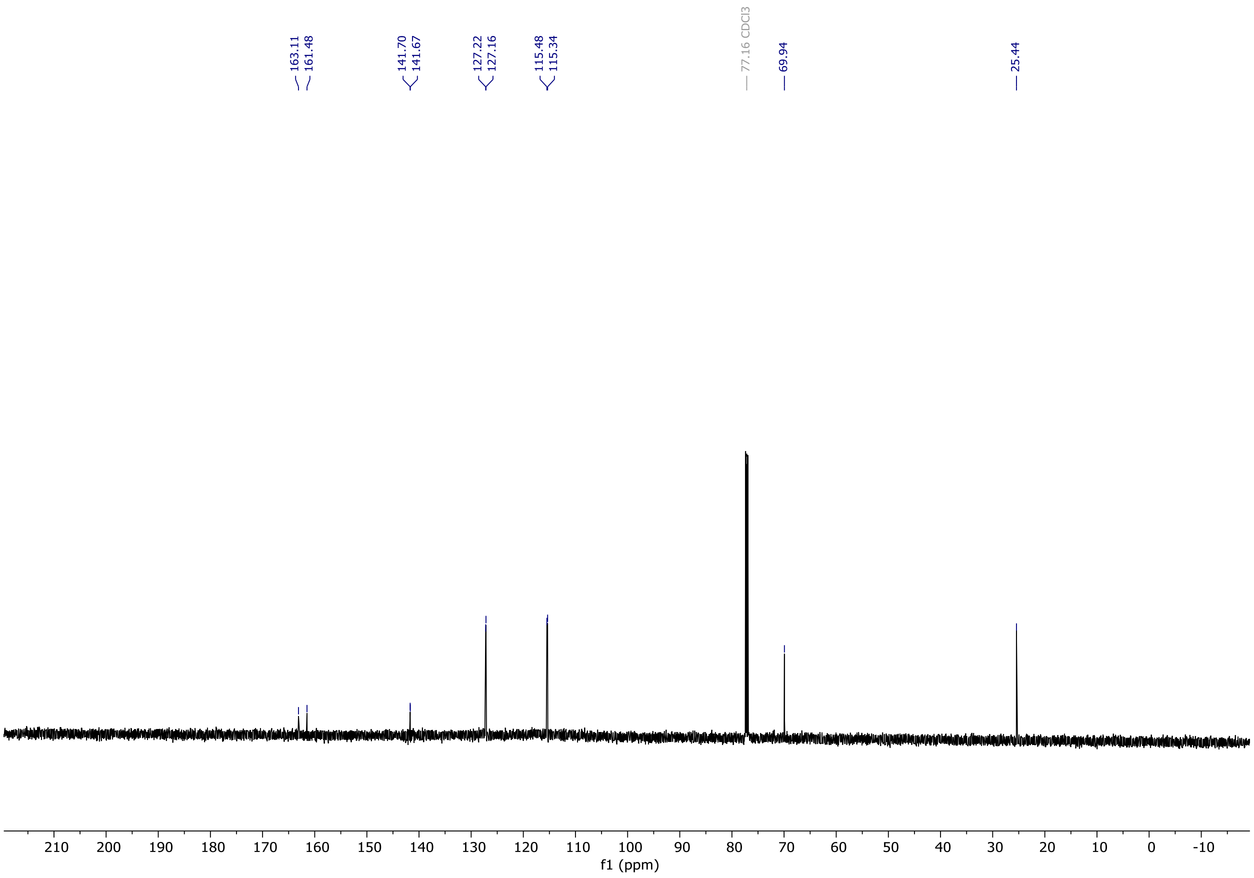

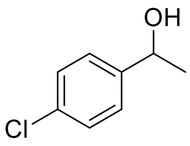

(**2c**)

^{1}H NMR (600 MHz, $CDCl_3$):

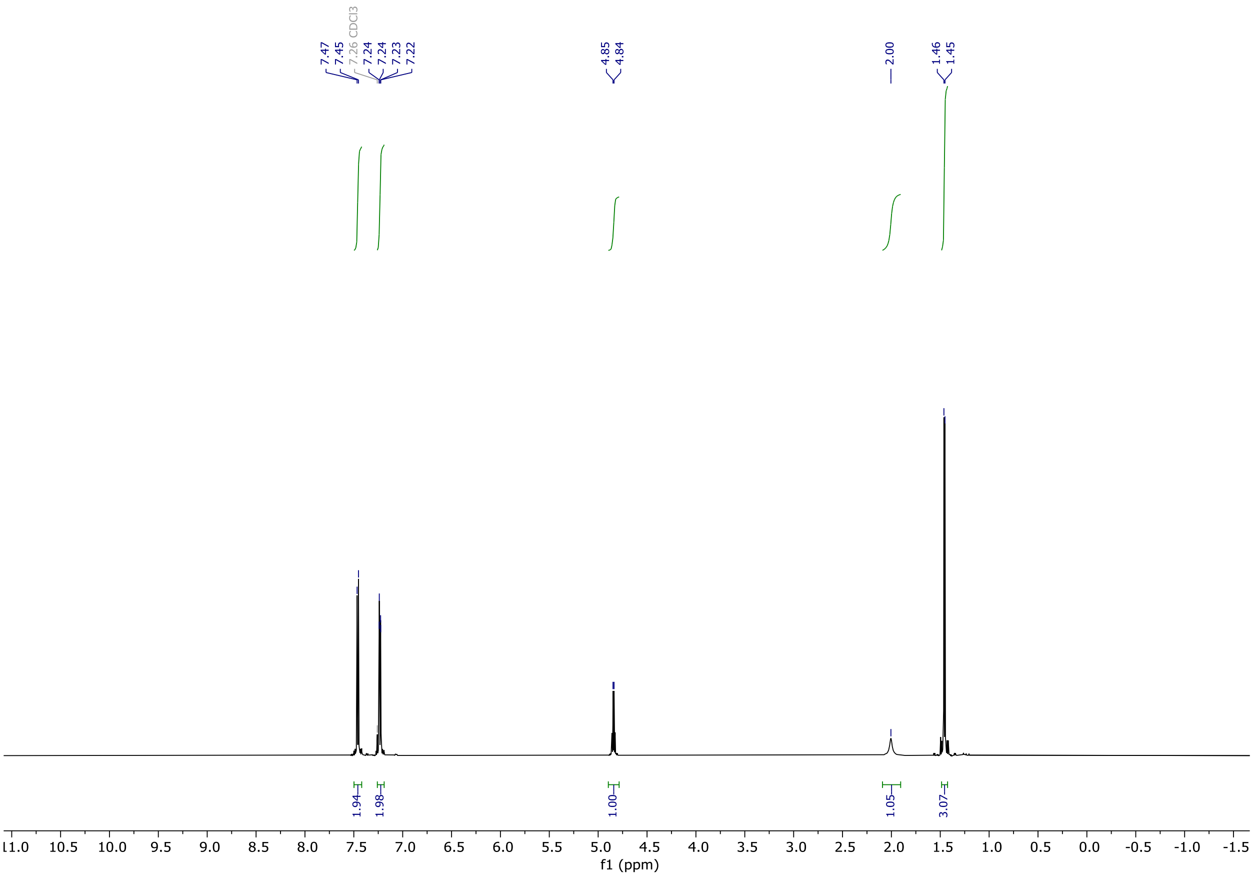

^{13}C NMR (151 MHz, $CDCl_3$):

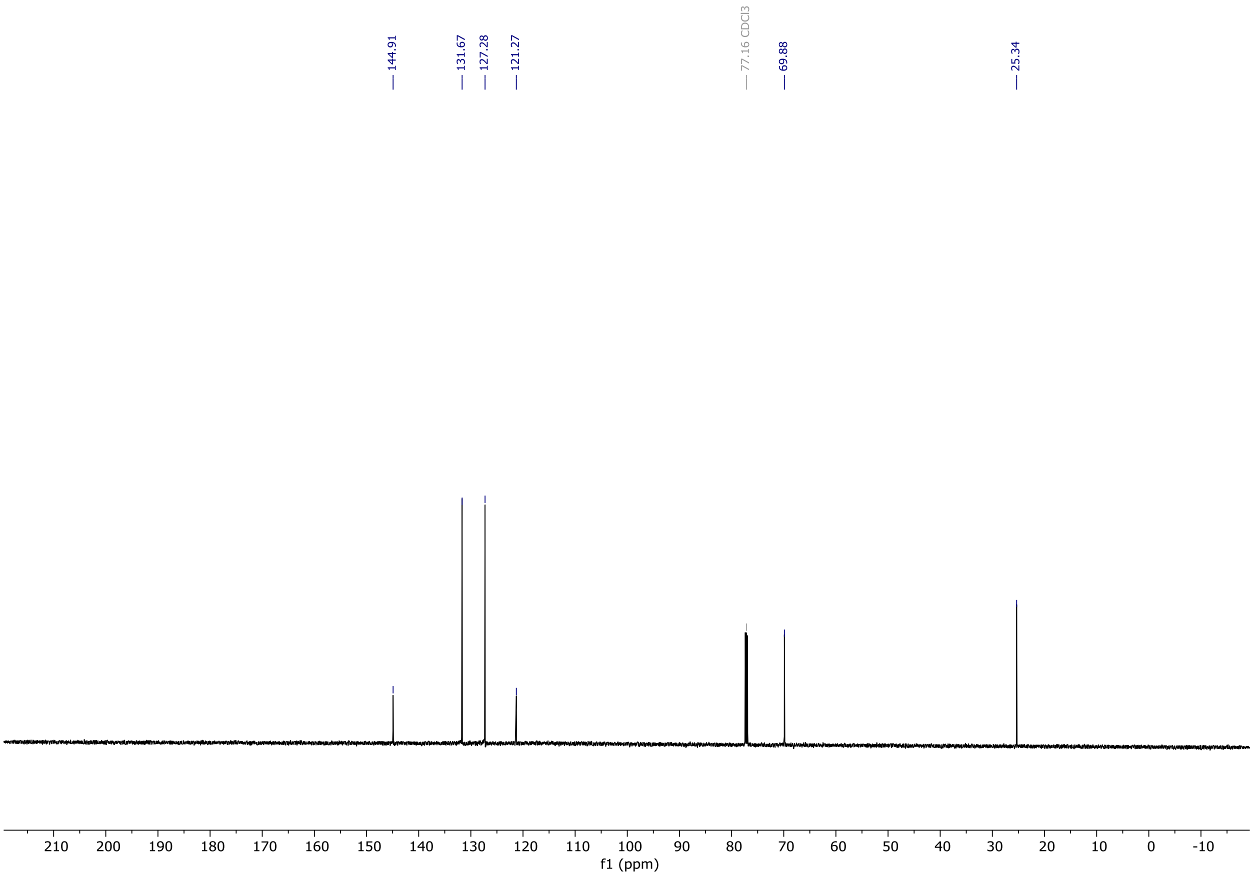

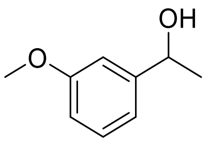

(**2d**)

^{1}H NMR (600 MHz, $CDCl_3$):

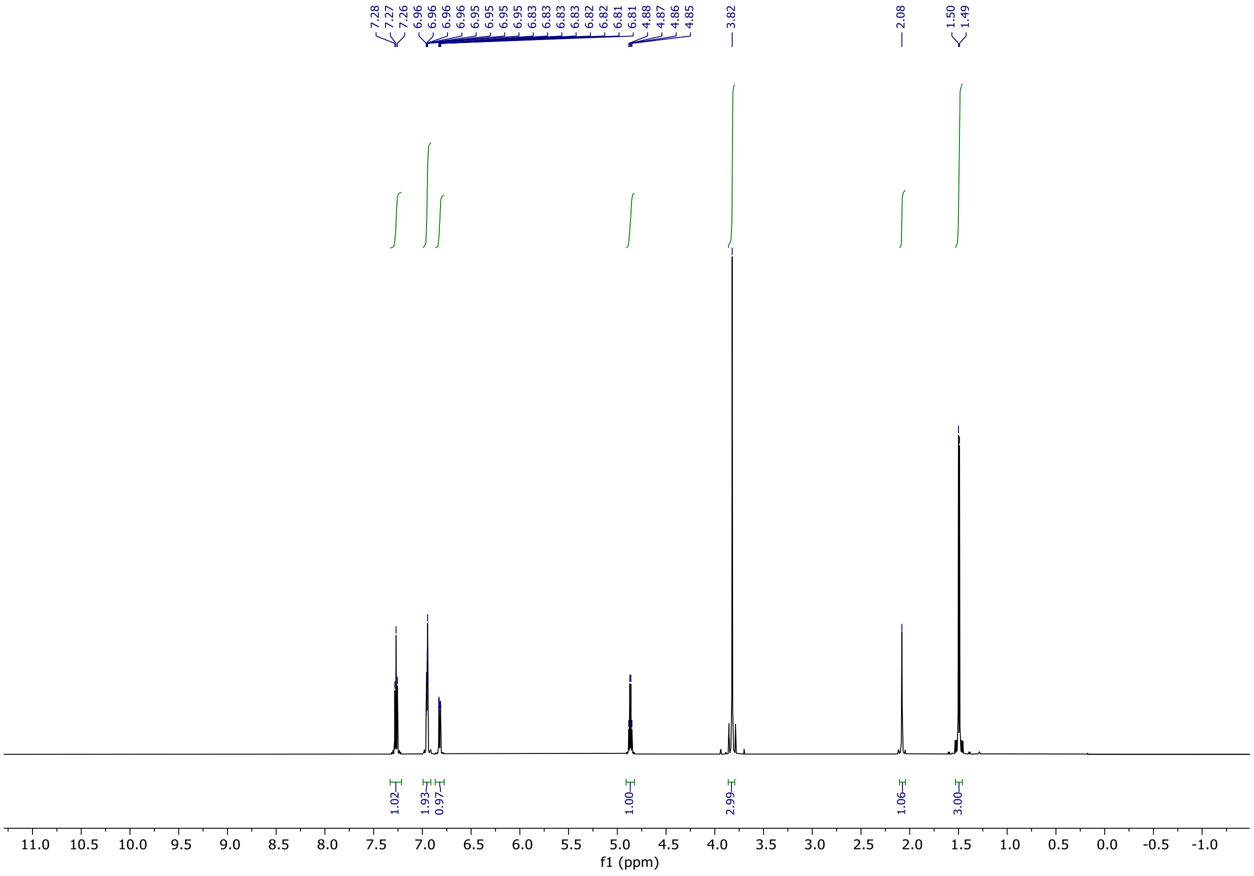

^{13}C NMR (151 MHz, $CDCl_3$):

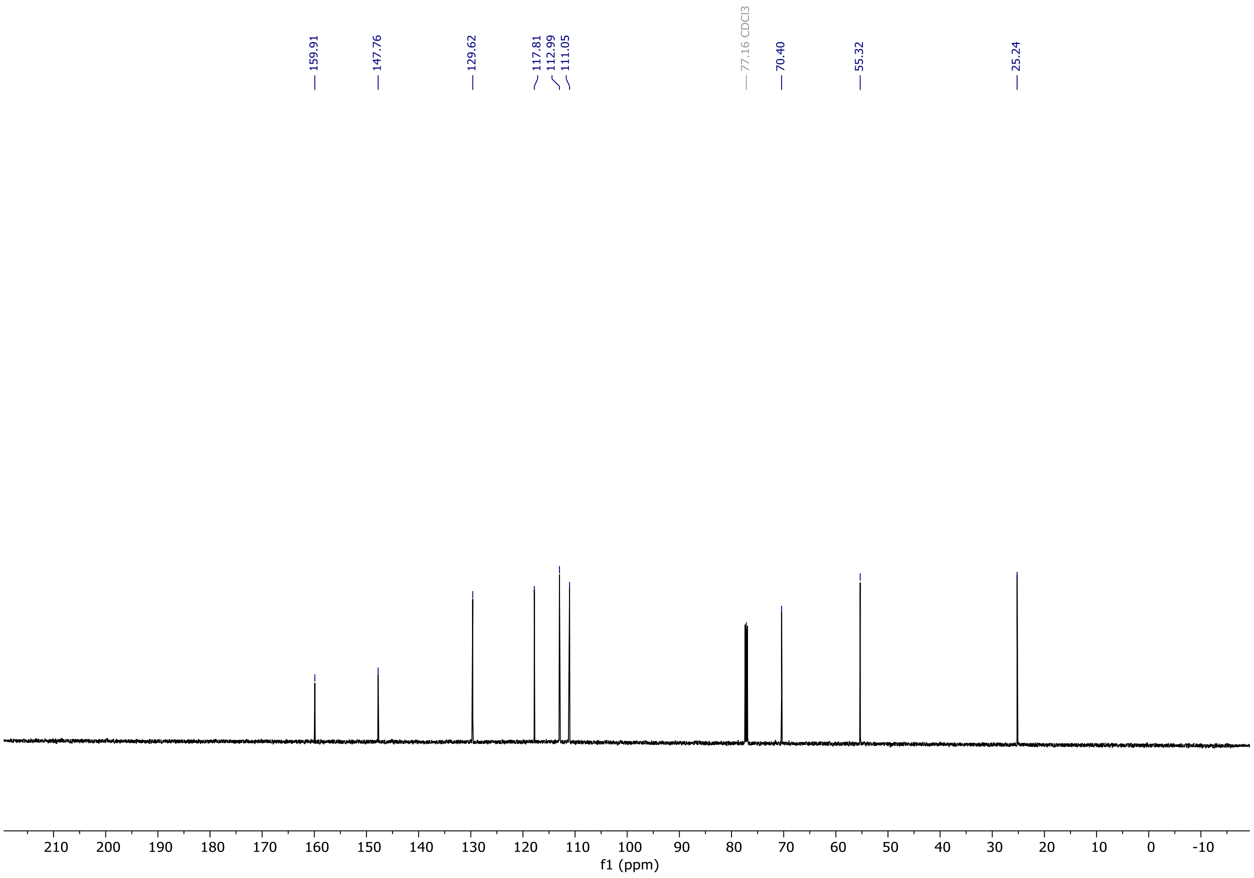

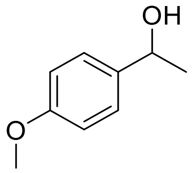

(**2e**)

^{1}H NMR (600 MHz, $CDCl_3$):

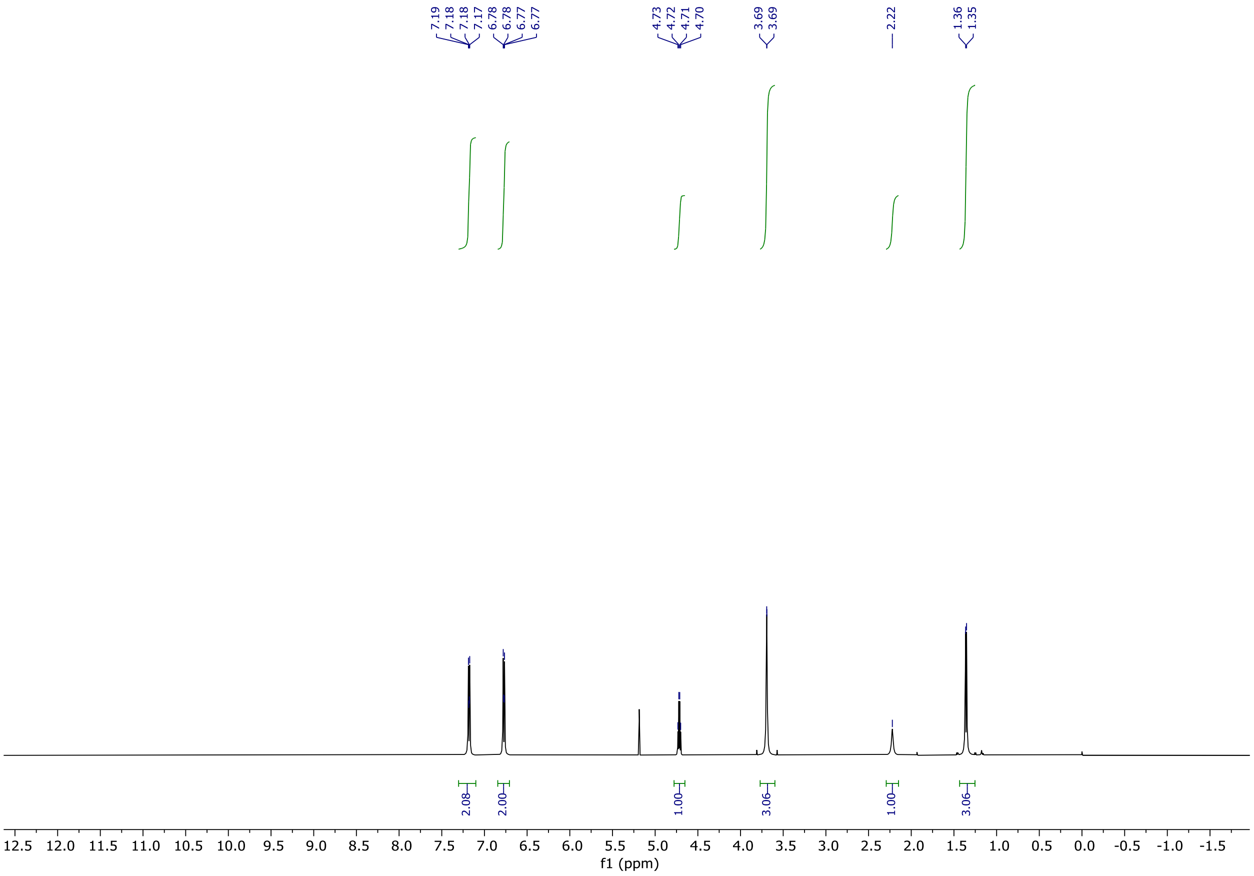

^{13}C NMR (151 MHz, $CDCl_3$):

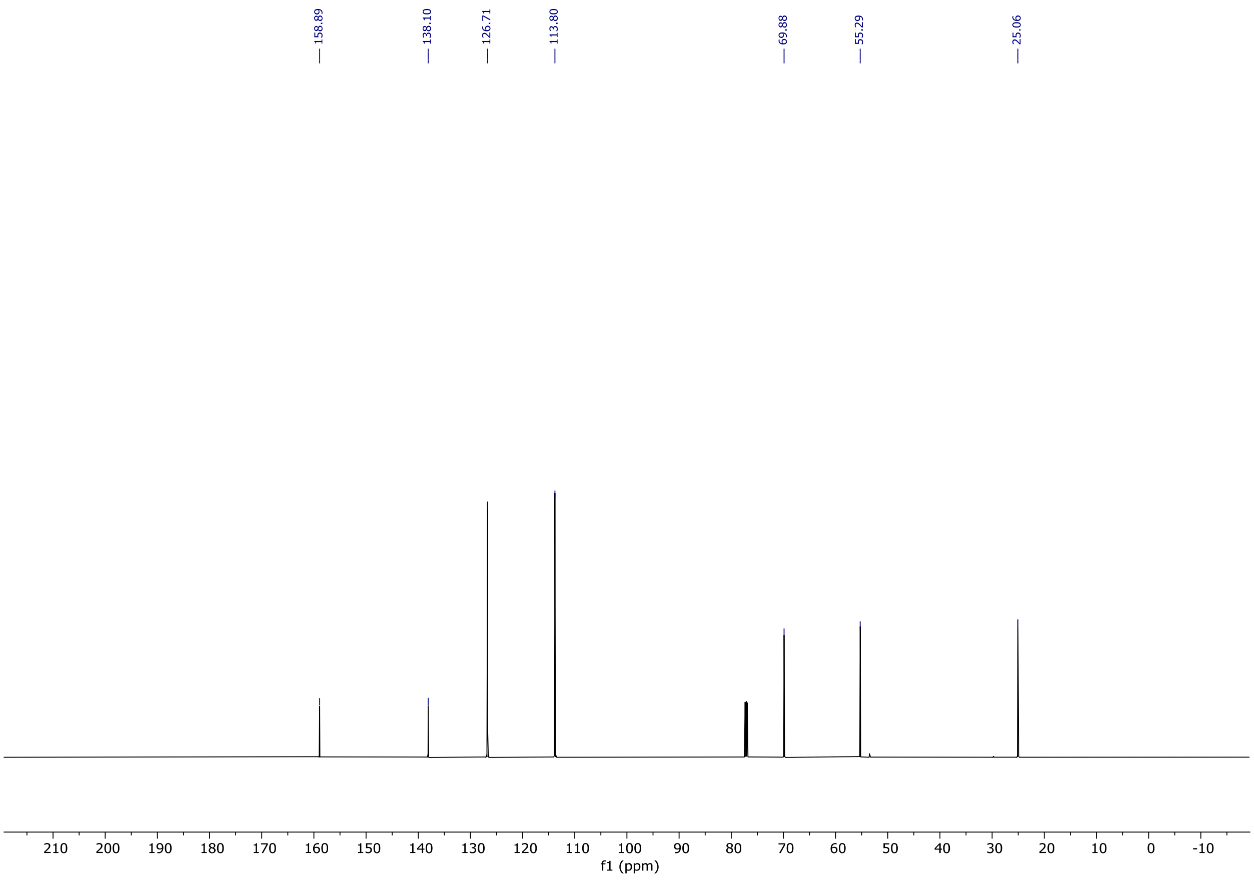

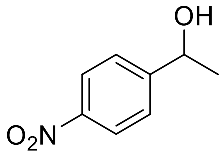

(**2f**)

^{1}H NMR (600 MHz, $CDCl_3$):

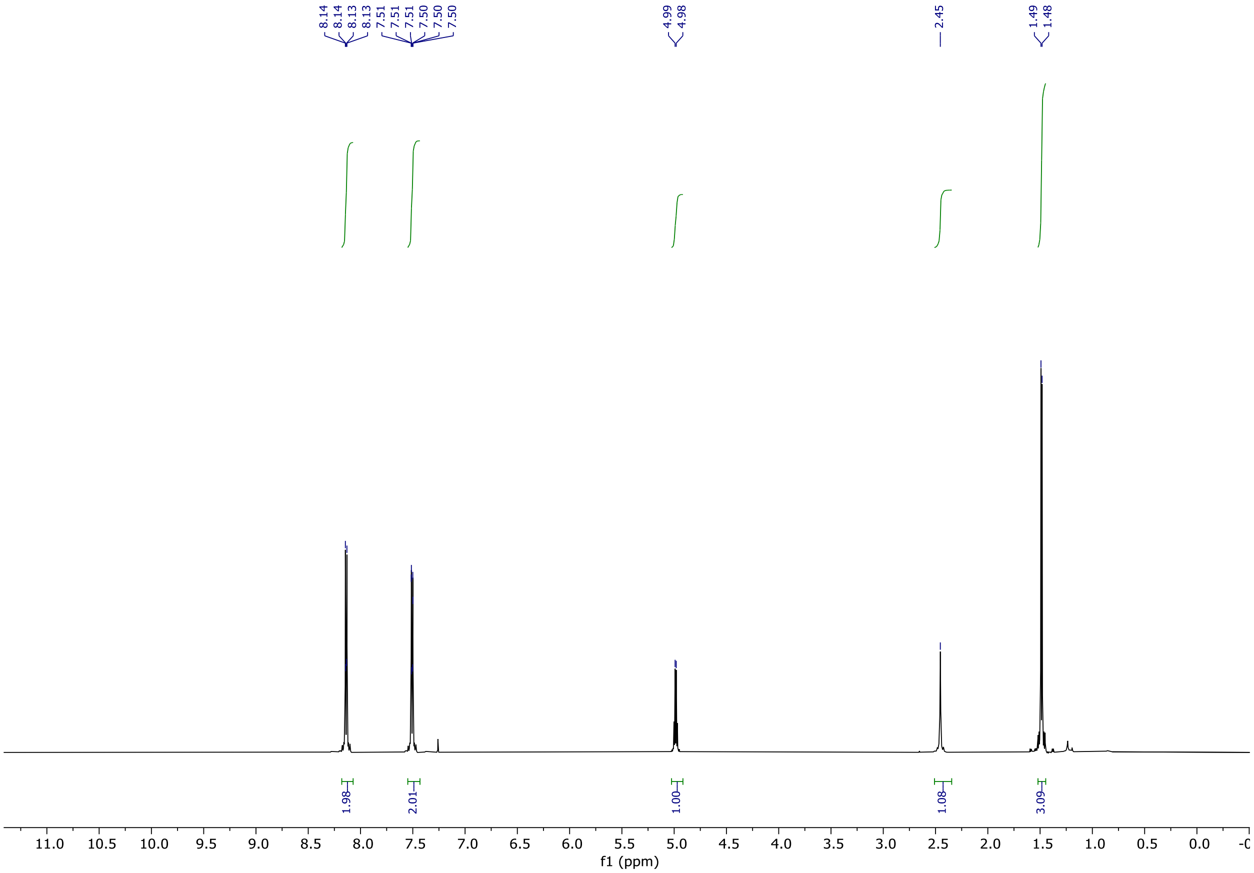

^{13}C NMR (151 MHz, $CDCl_3$):

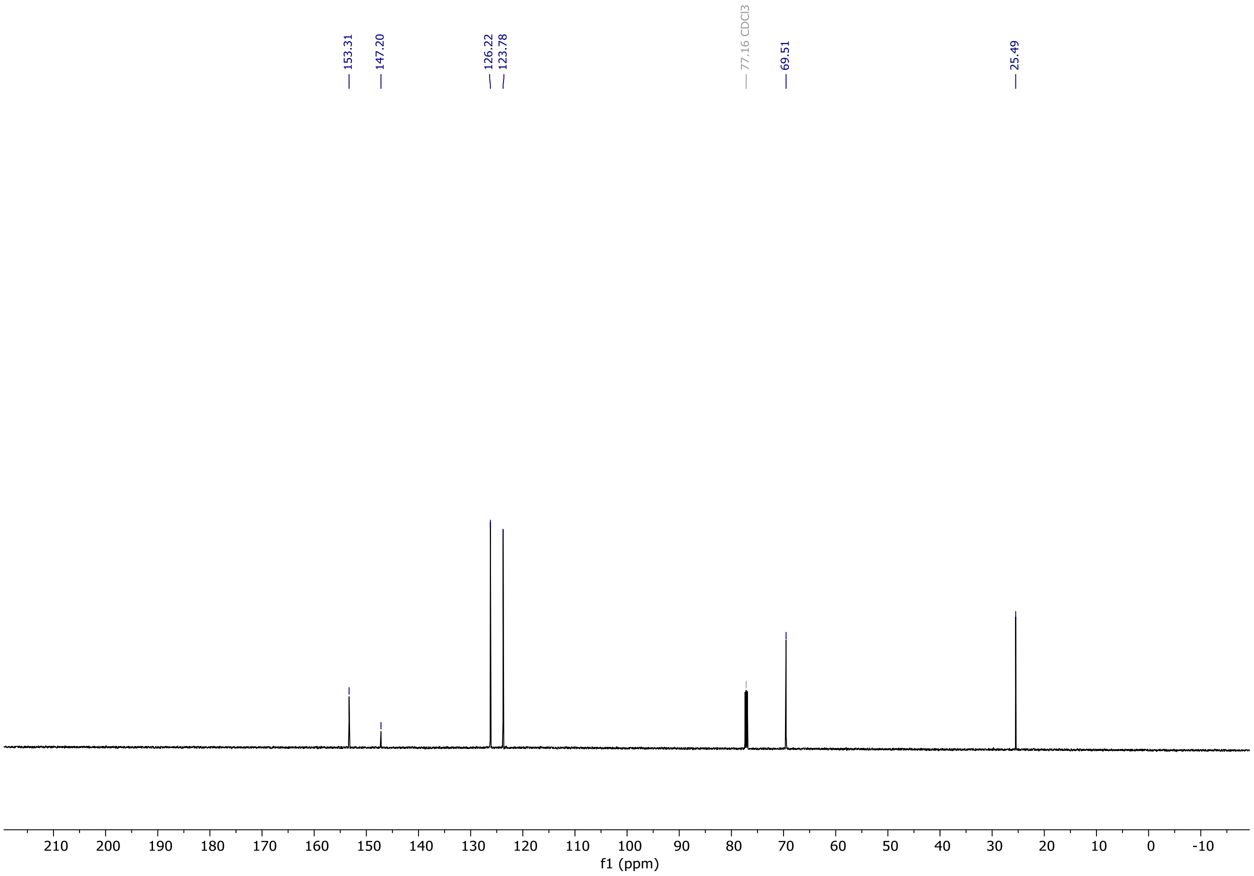

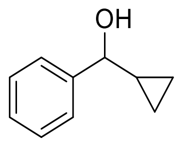

(**2g**)

^{1}H NMR (500 MHz, $CDCl_3$):

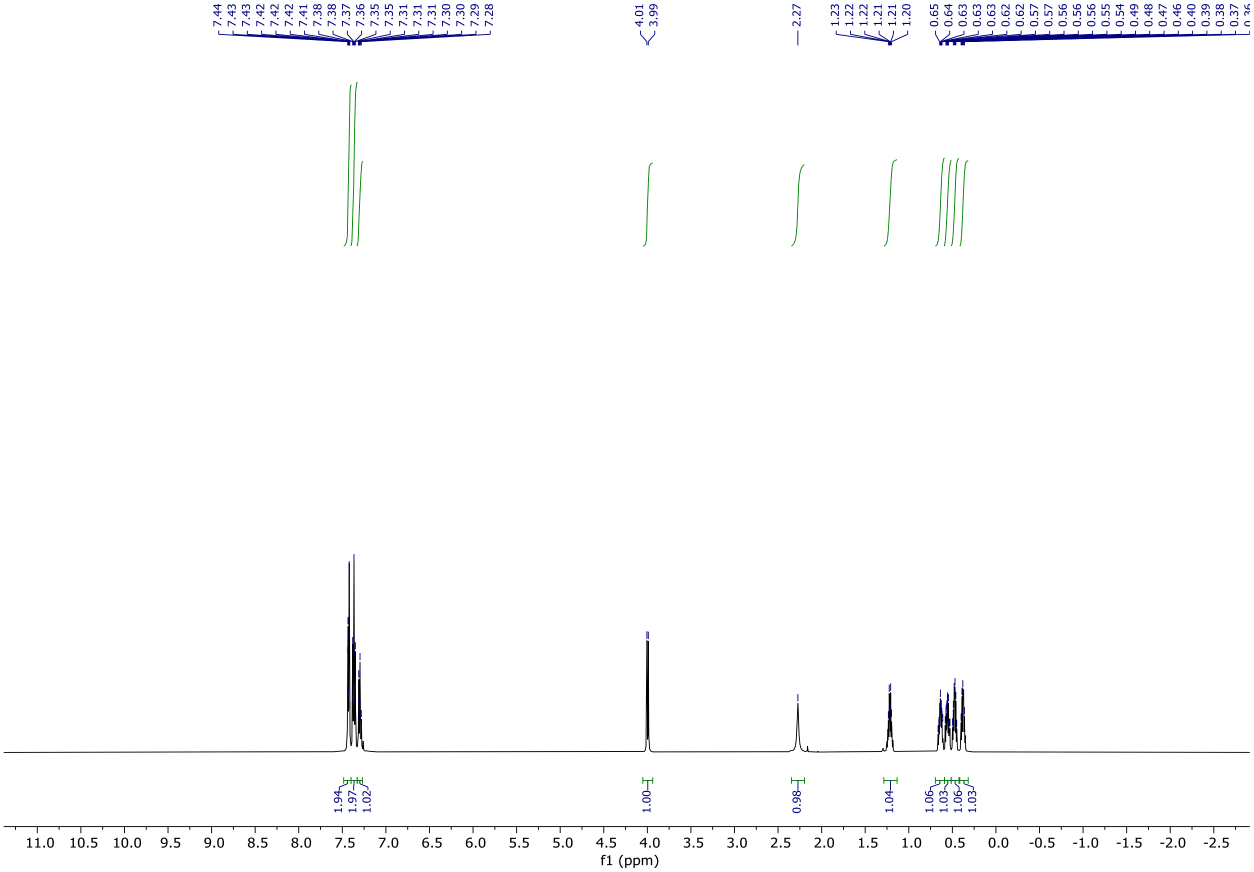

^{13}C NMR (126 MHz, $CDCl_3$):

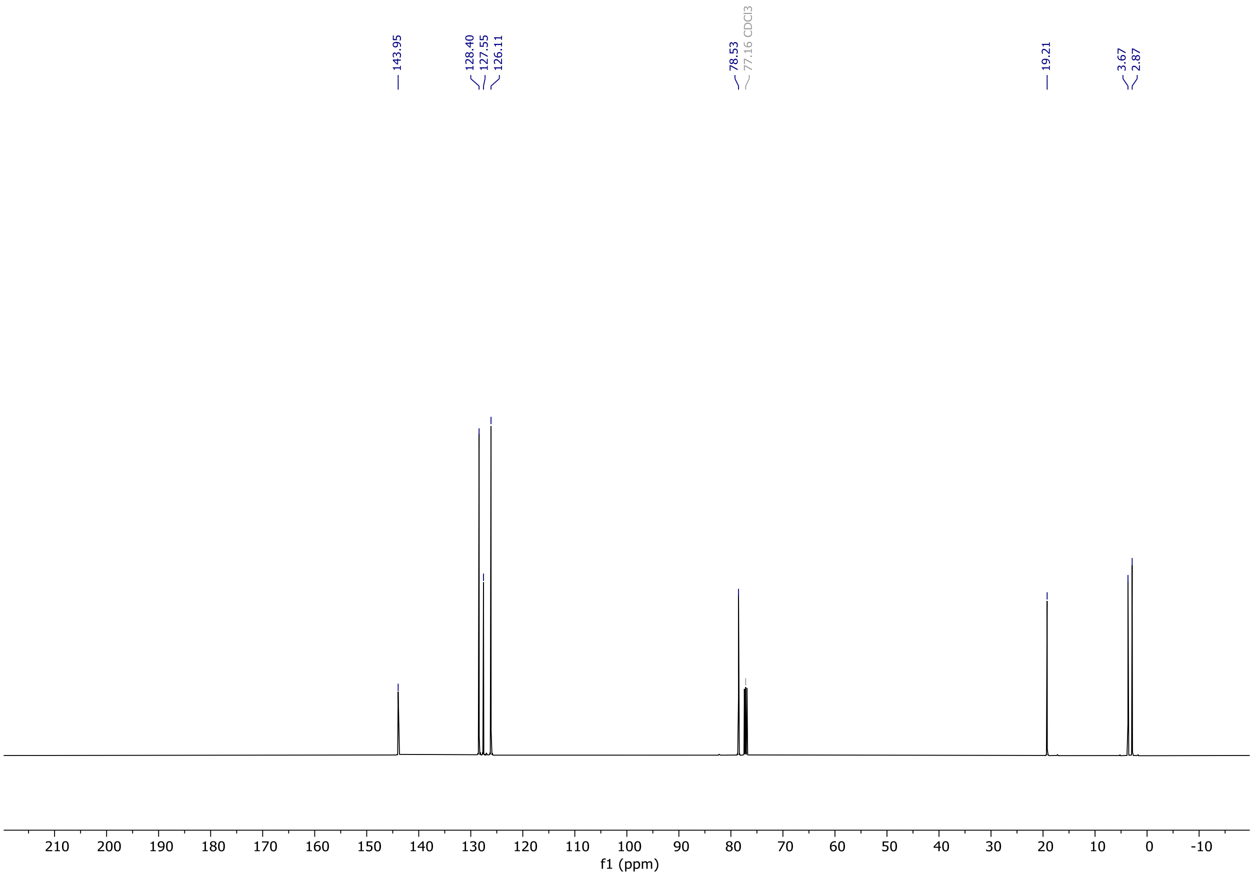

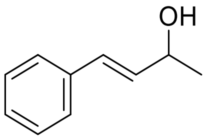

(**2h**)

^{1}H NMR (600 MHz, $CDCl_3$):

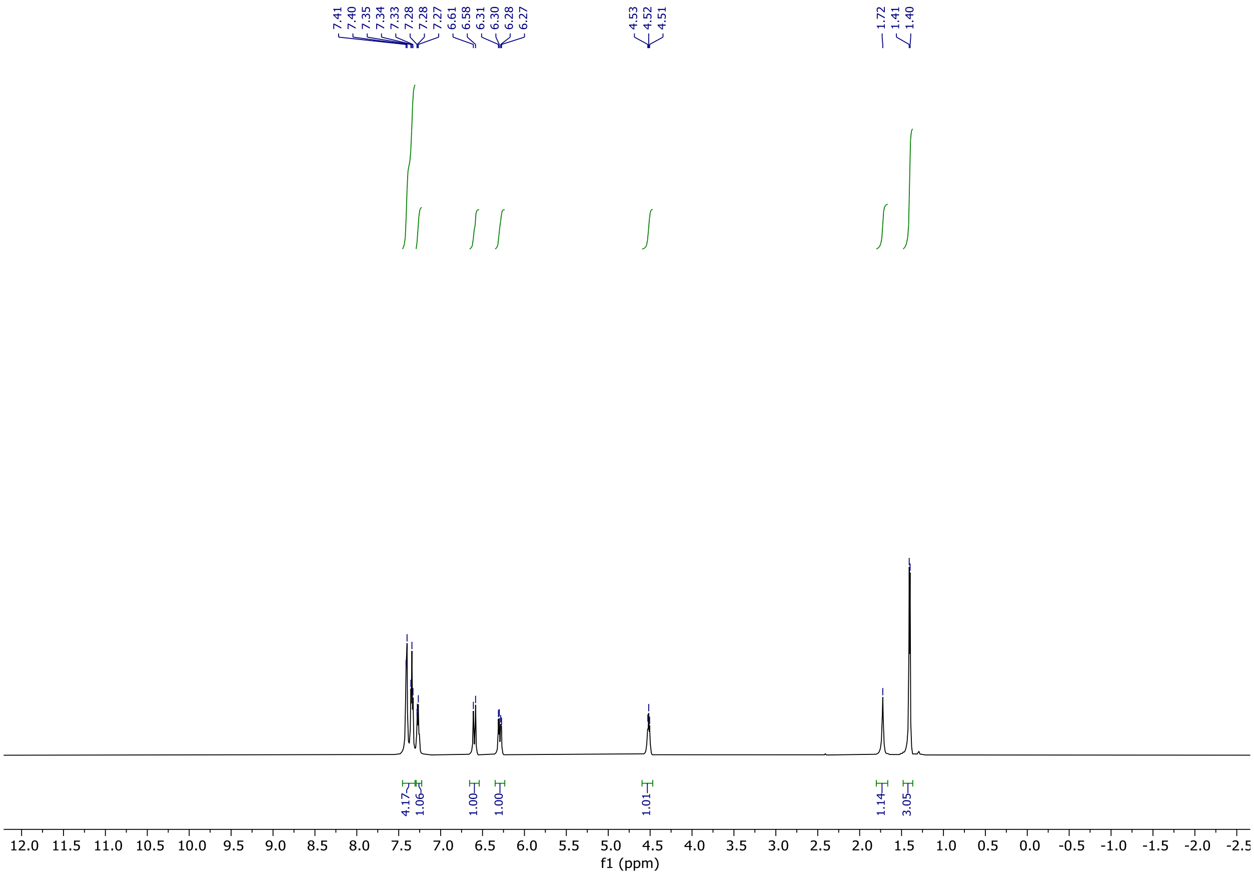

^{13}C NMR (151 MHz, $CDCl_3$):

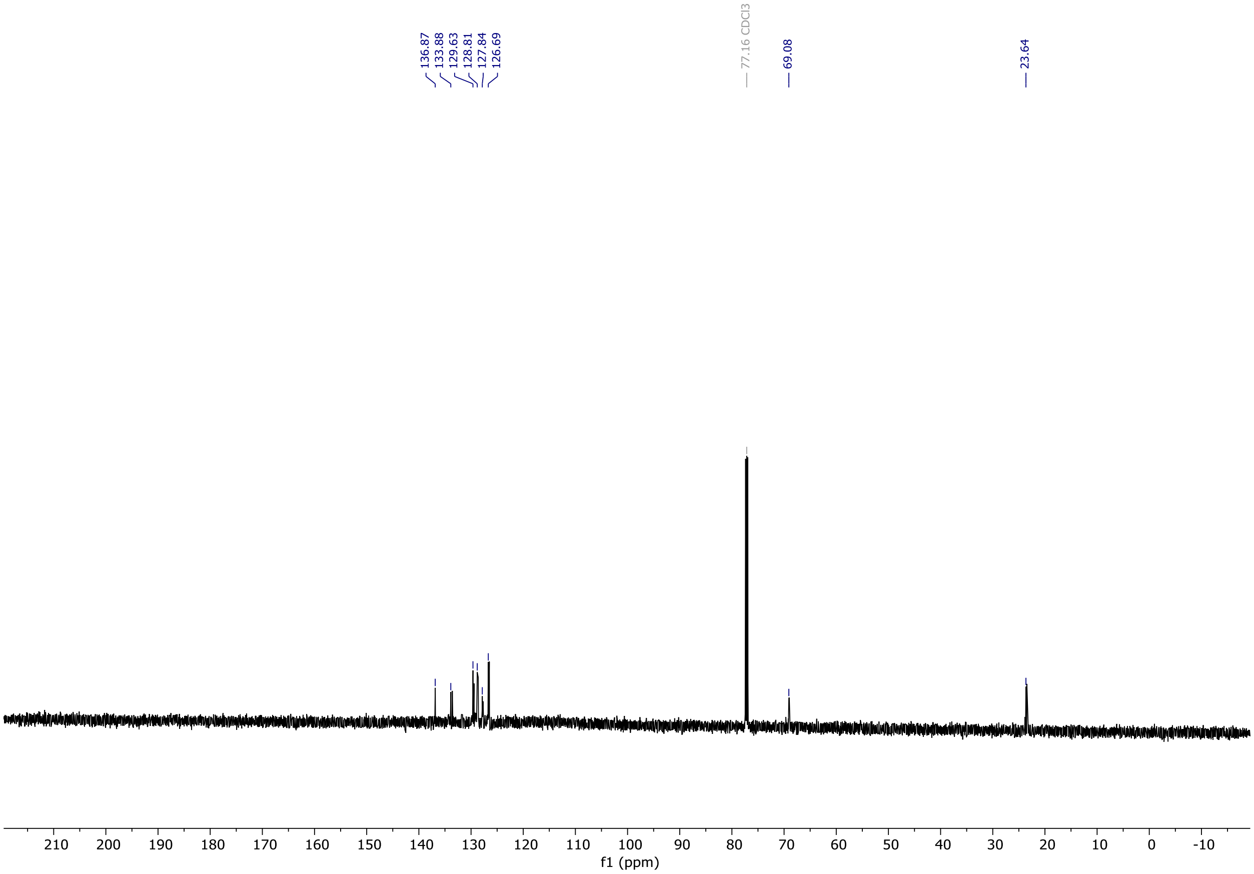

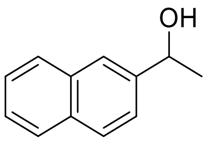

(**2i**)

^{1}H NMR (600 MHz, $CDCl_3$):

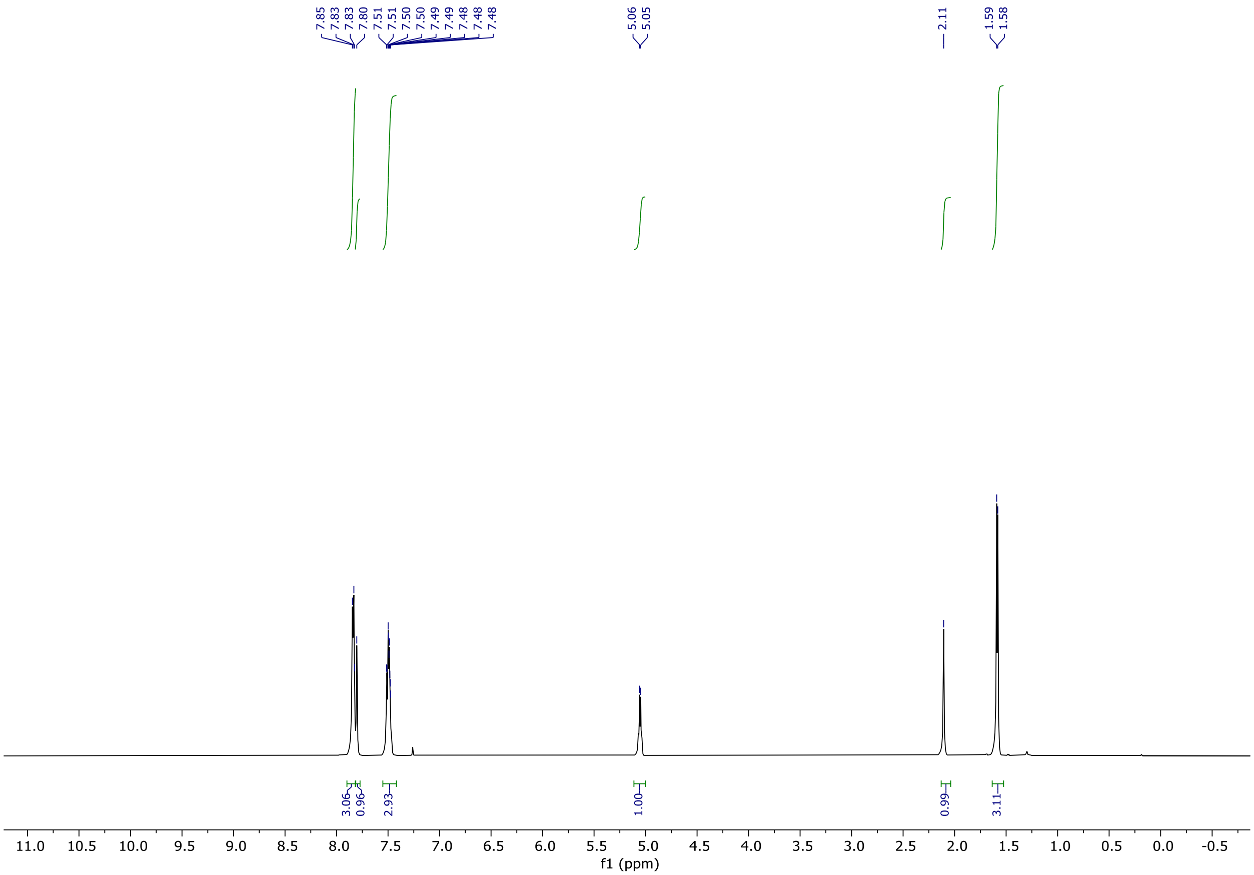

^{13}C NMR (151 MHz, $CDCl_3$):

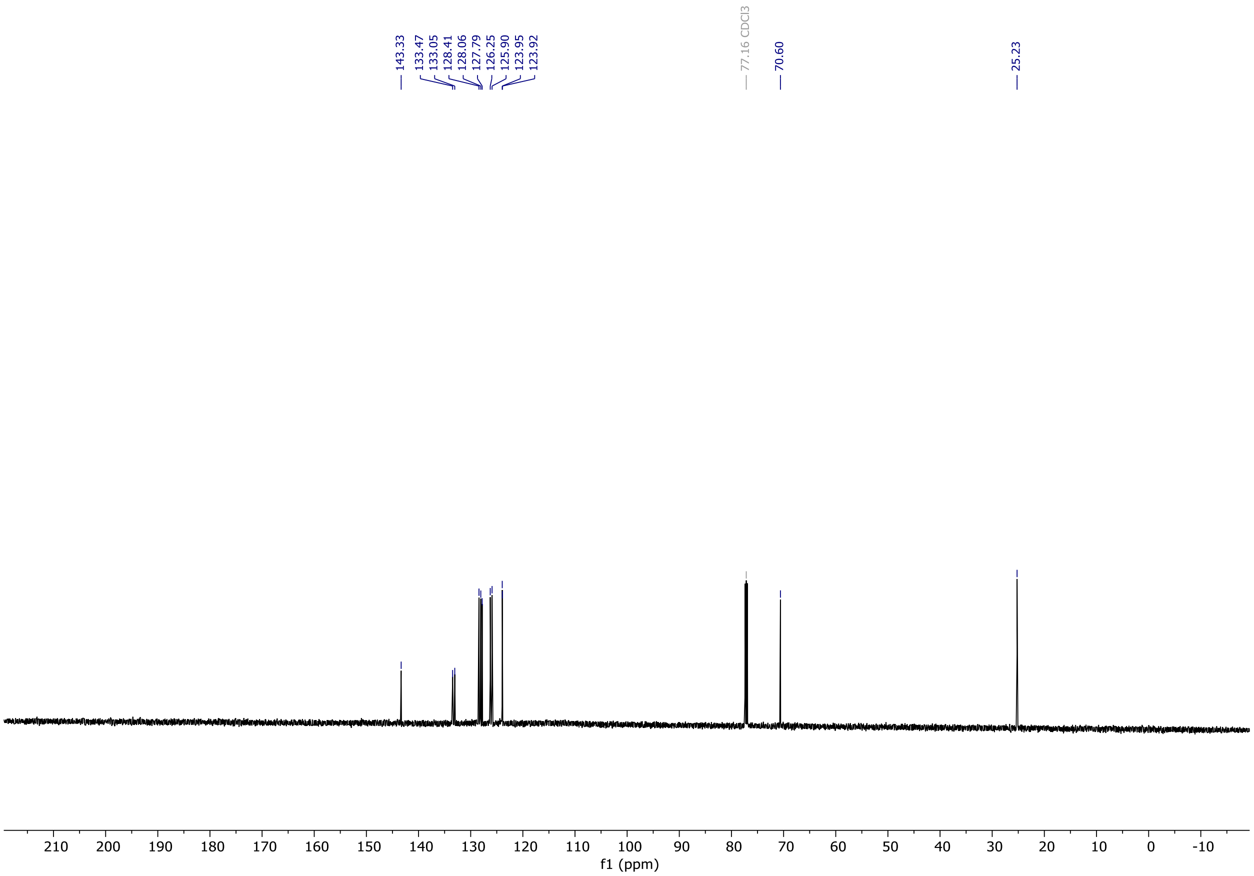

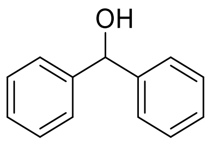
(**2j**)

^{1}H NMR (600 MHz, $CDCl_3$):

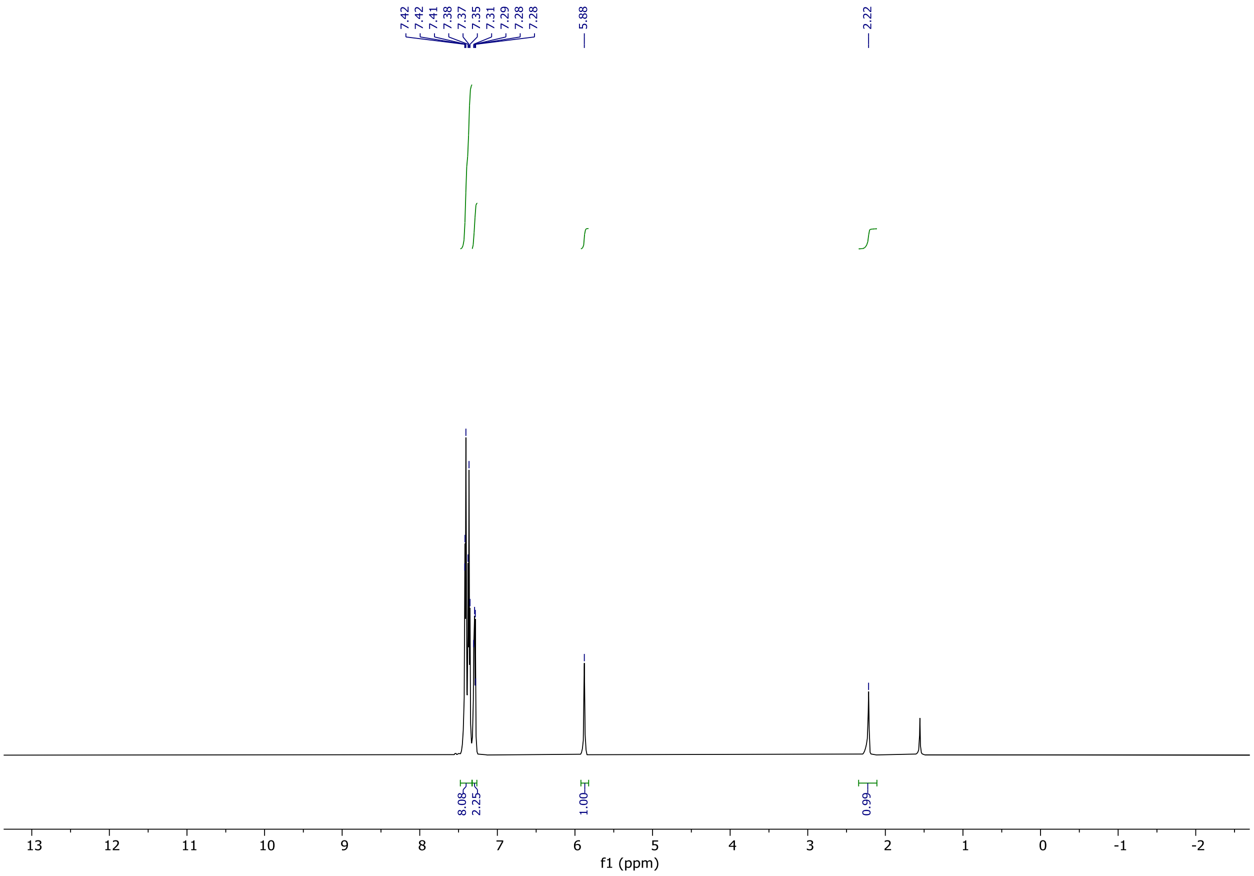

^{13}C NMR (151 MHz, $CDCl_3$):

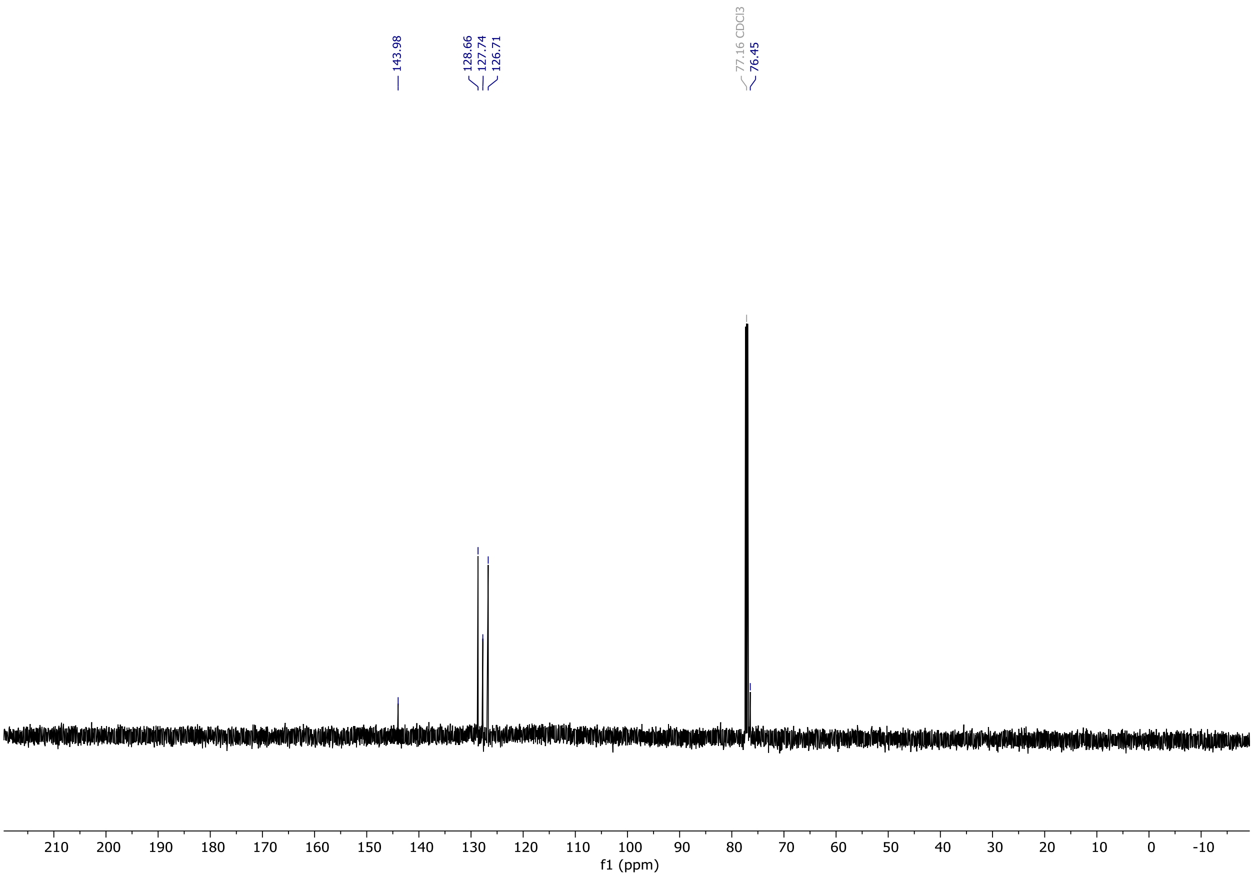

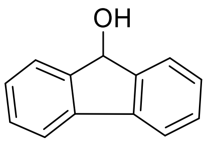

(**2k**)

^{1}H NMR (600 MHz, CD_3OD):

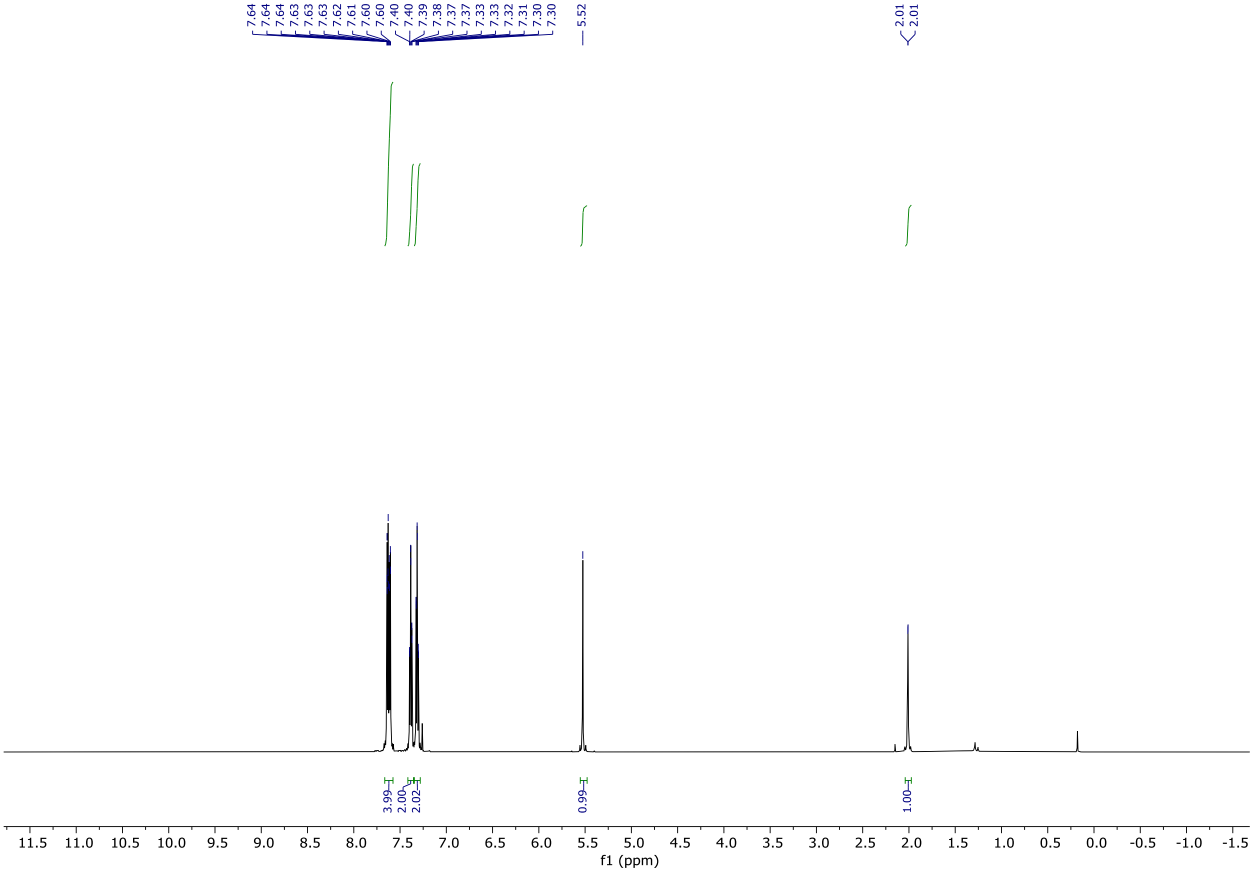

^{13}C NMR (151 MHz, CD_3OD):

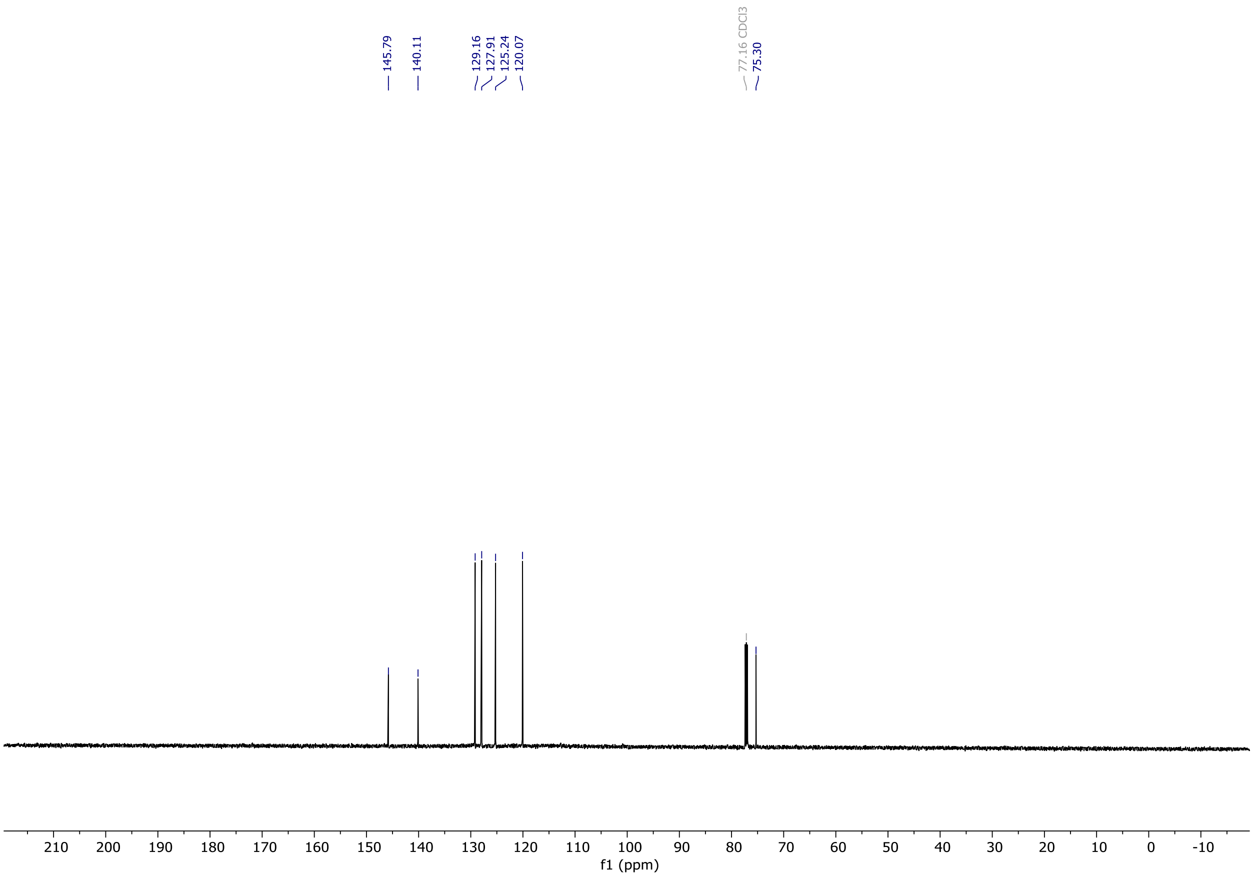

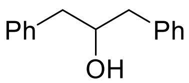

(**2l**)

^{1}H NMR (600 MHz, $CDCl_3$):

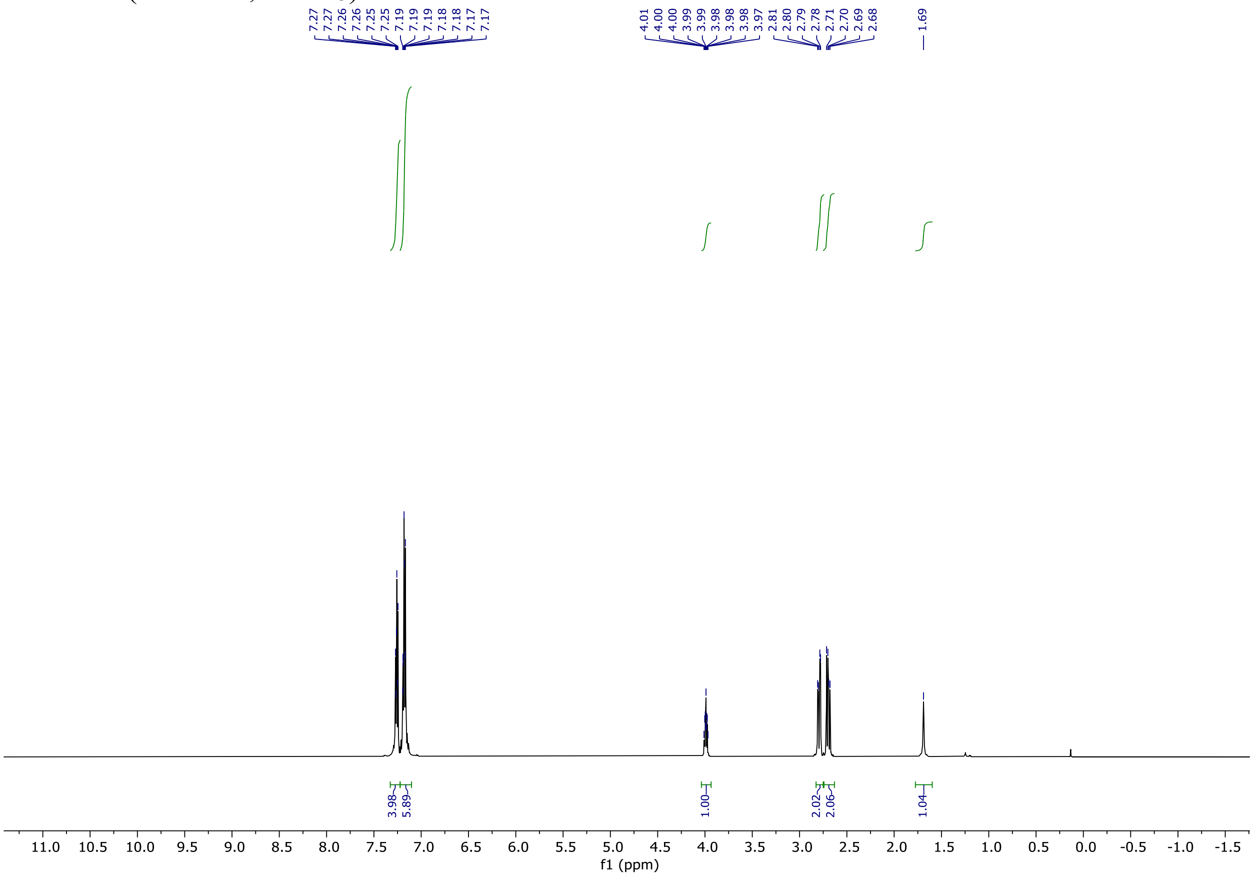

^{13}C NMR (151 MHz, $CDCl_3$):

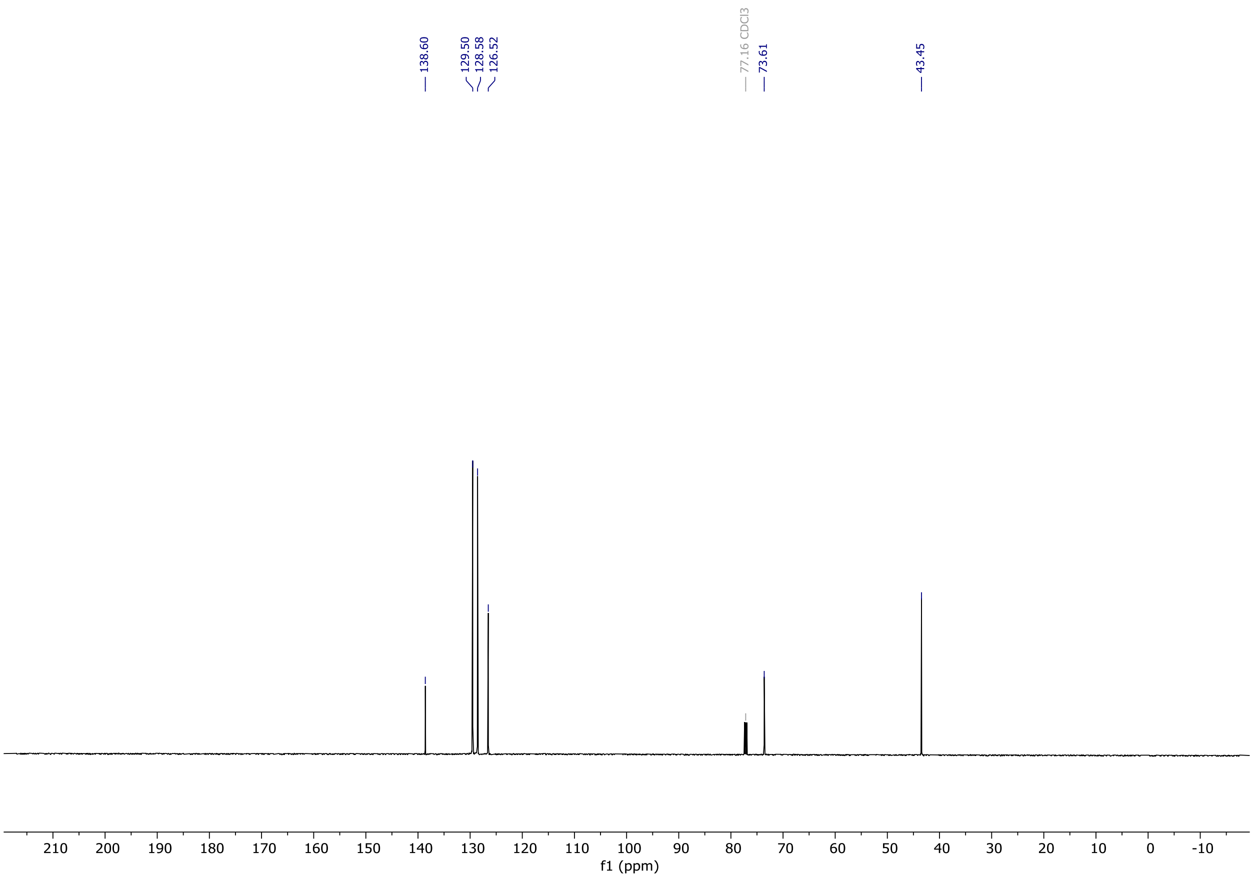

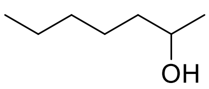

(**2m**)

^{1}H NMR (600 MHz, $CDCl_3$):

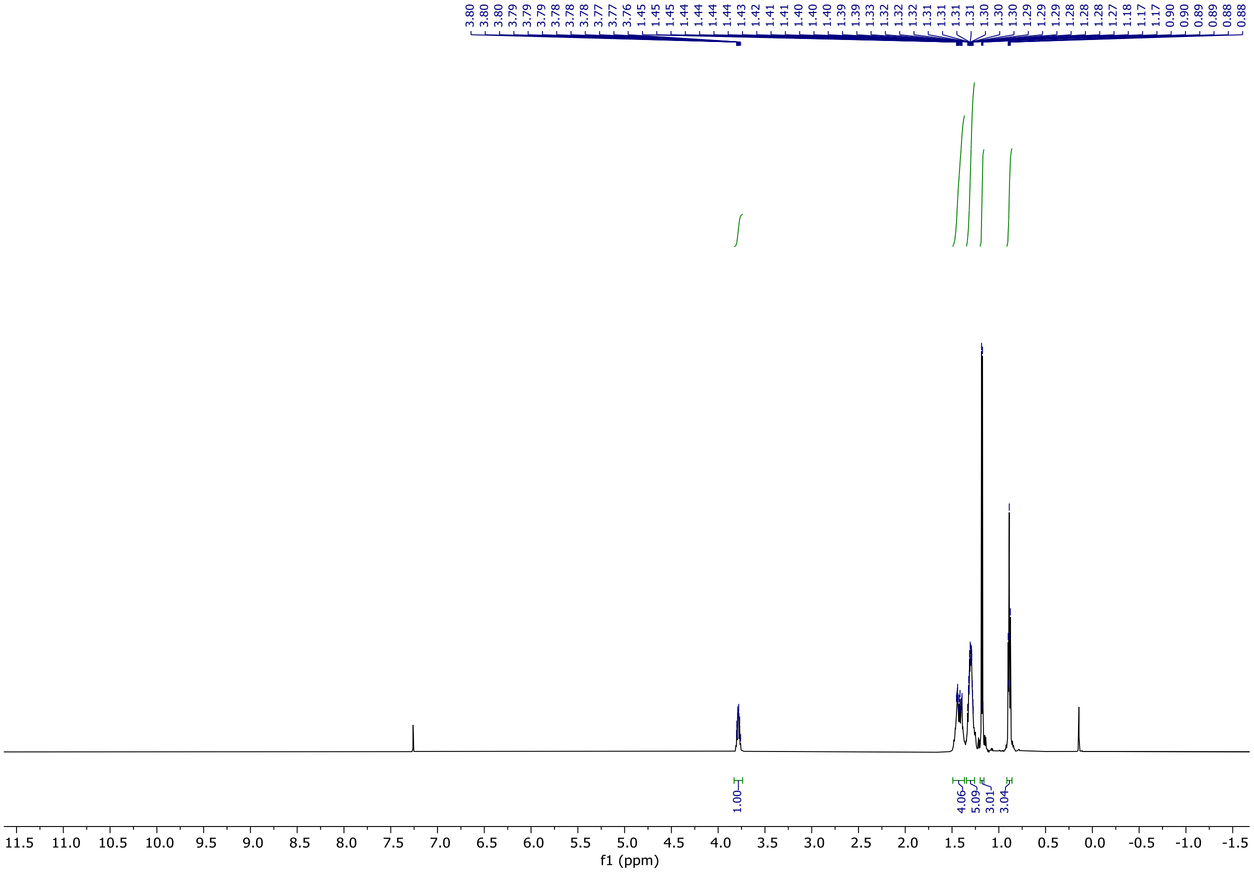

^{13}C NMR (151 MHz, $CDCl_3$):

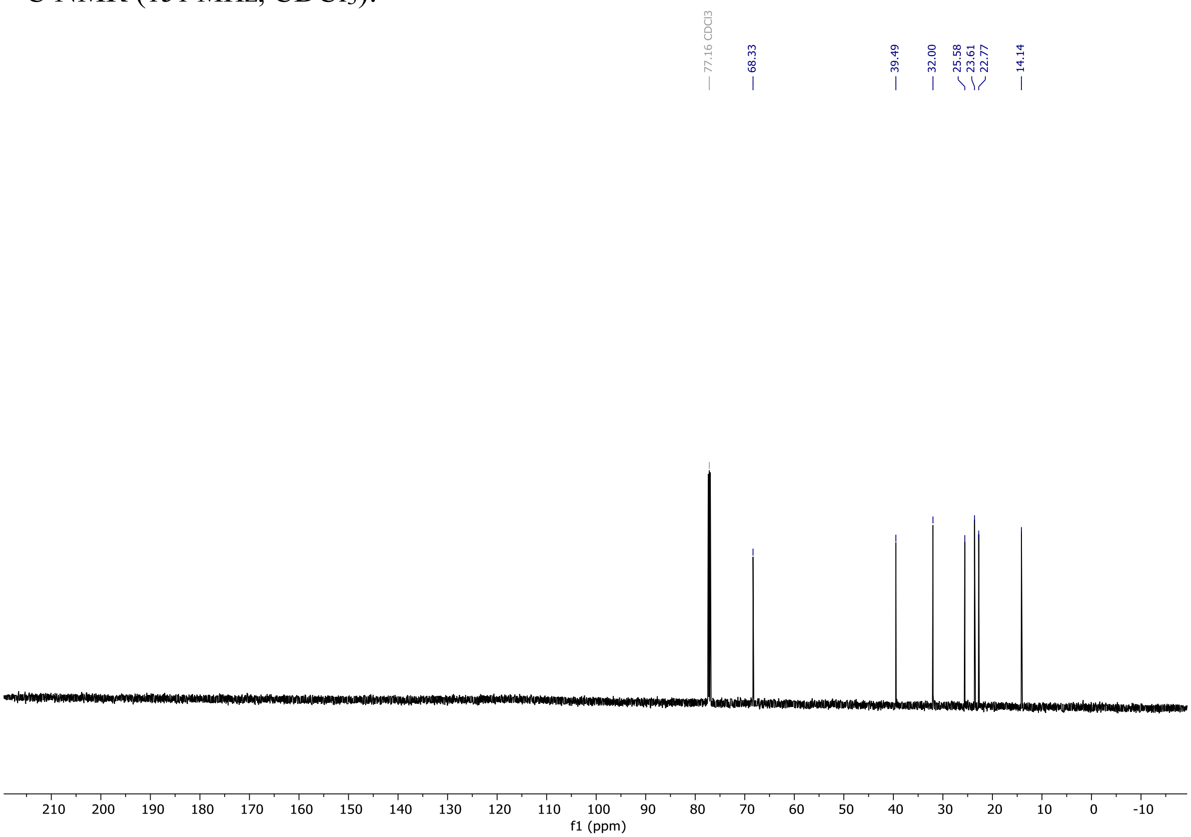

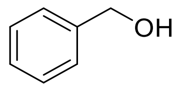

(**3a**)

^{1}H NMR (500 MHz, $CDCl_3$):

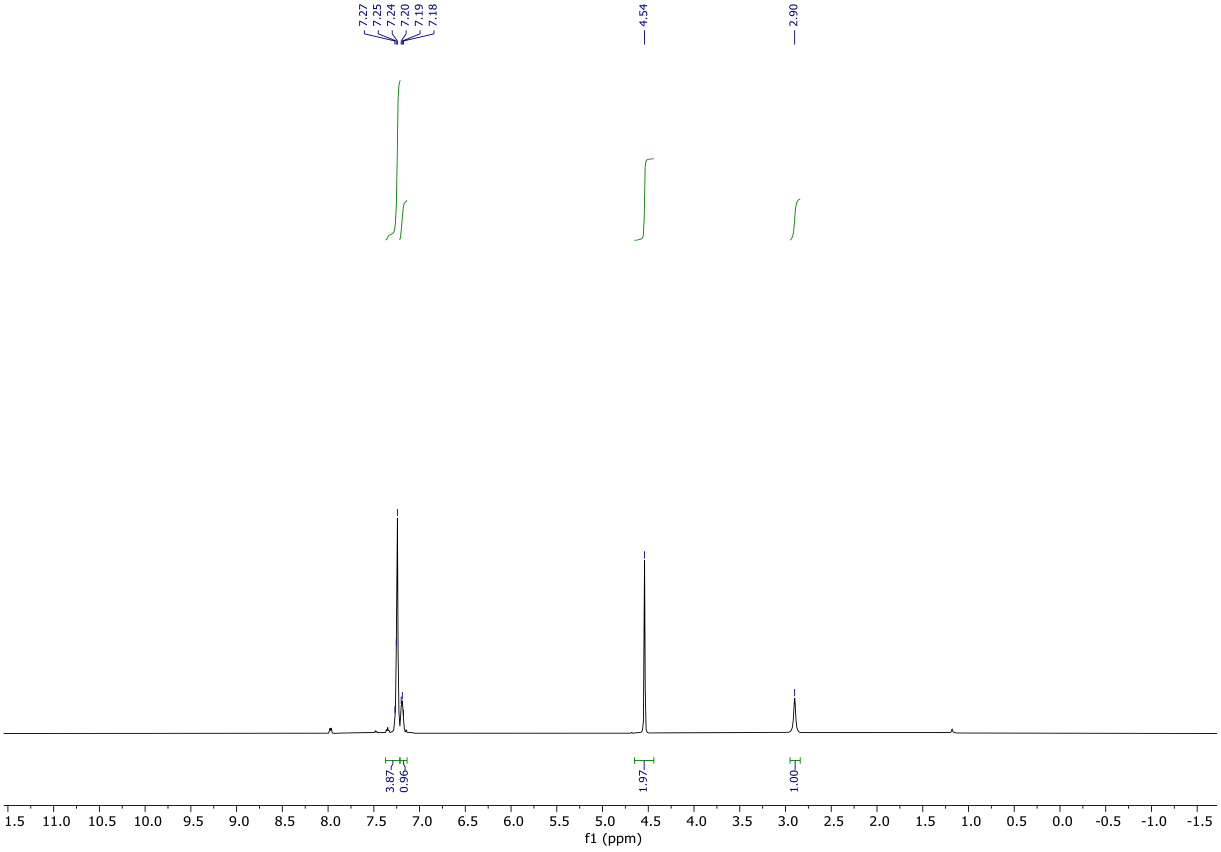

^{13}C NMR (126 MHz, $CDCl_3$):

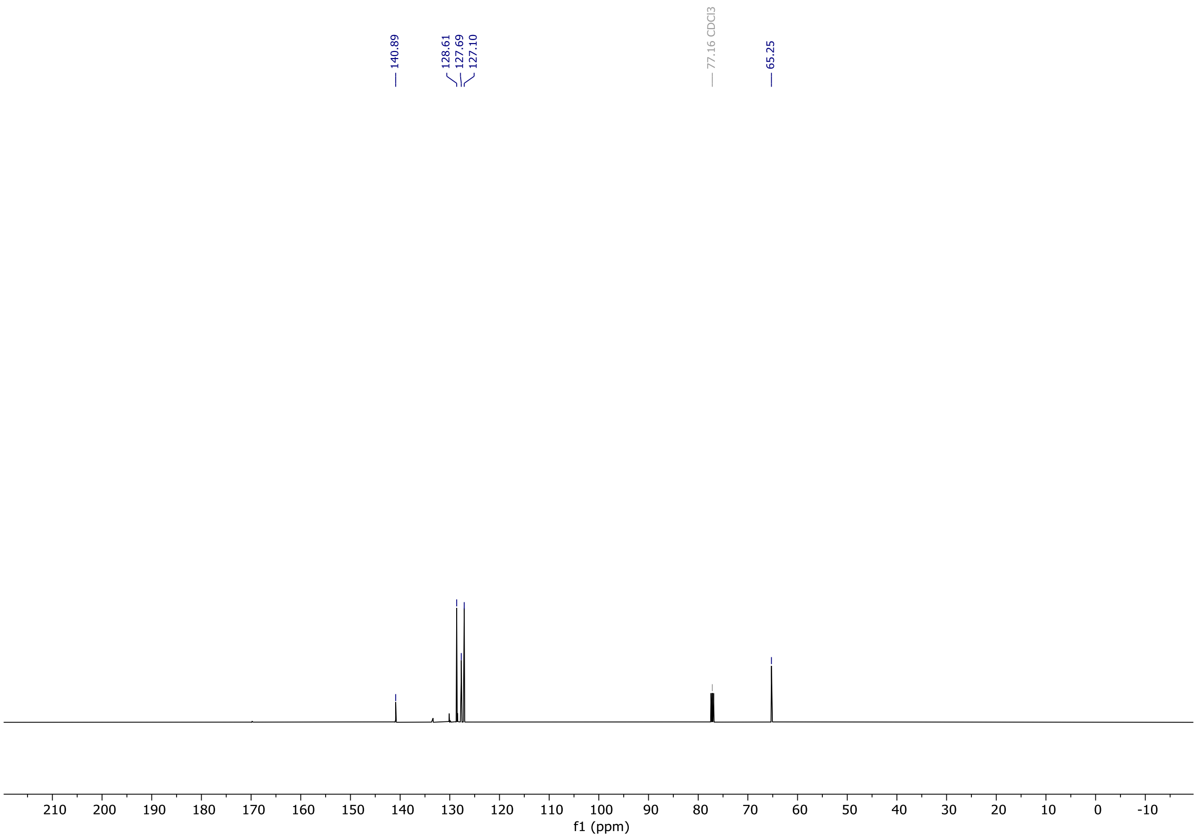

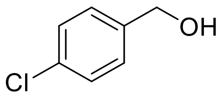

(**3b**)

^{1}H NMR (600 MHz, $CDCl_3$):

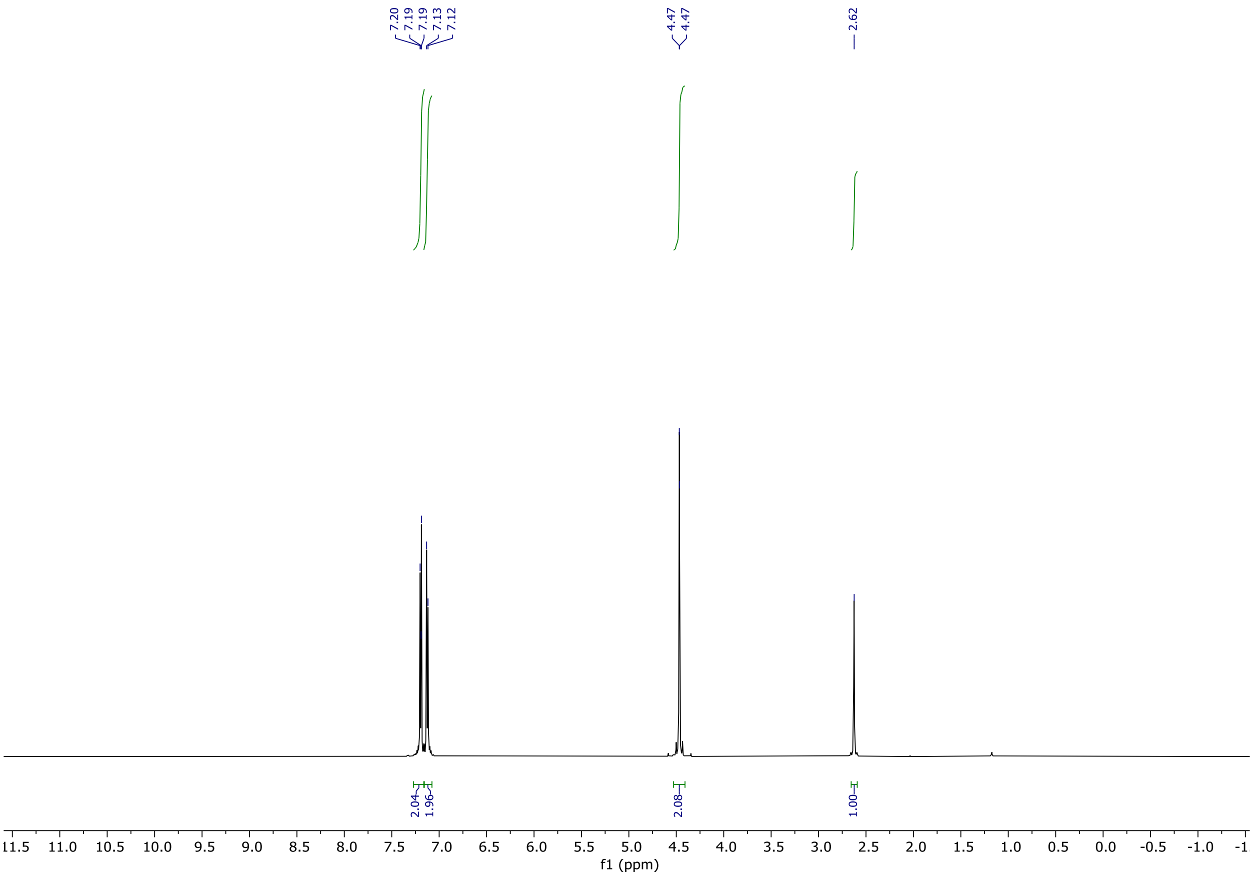

^{13}C NMR (151 MHz, $CDCl_3$):

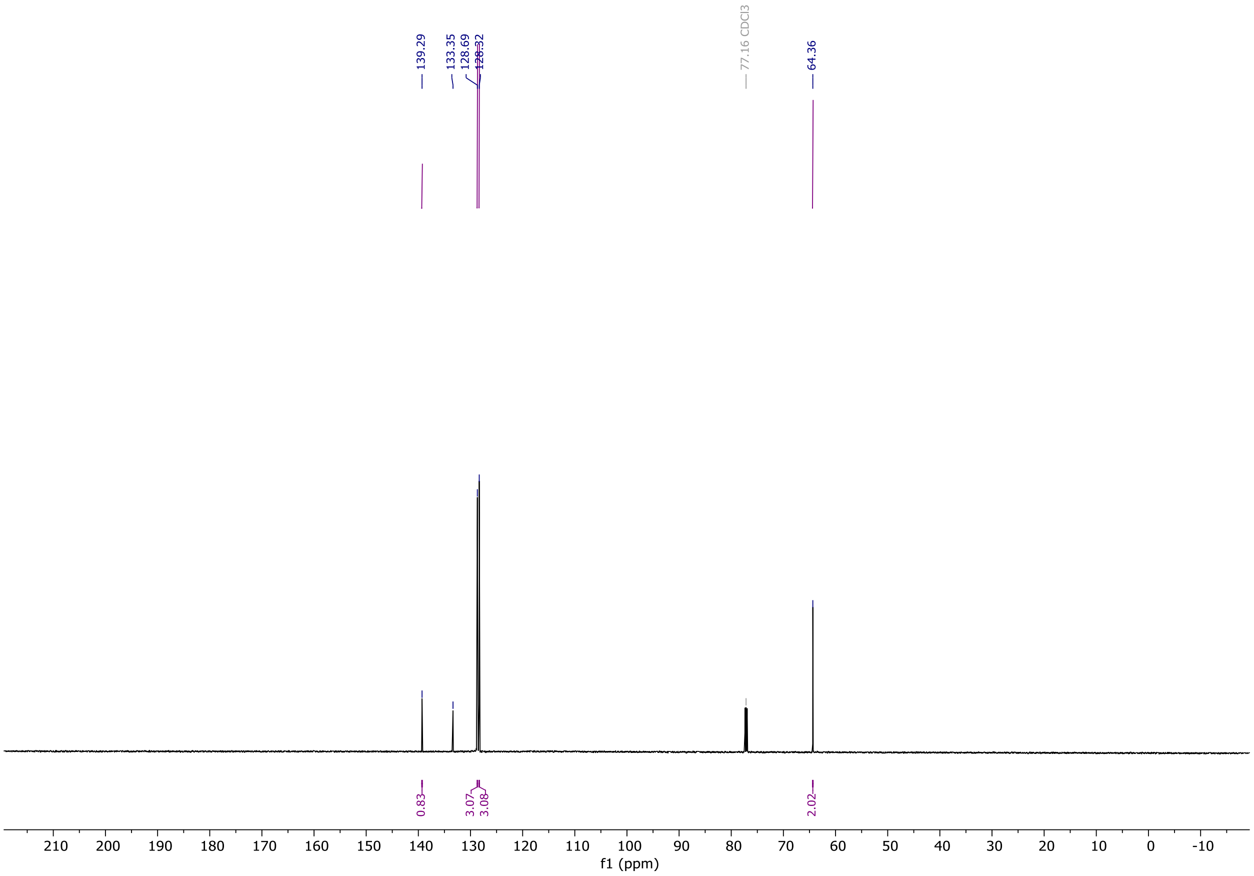

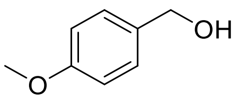

(**3c**)

^{1}H NMR (500 MHz, CDCl$_3$):

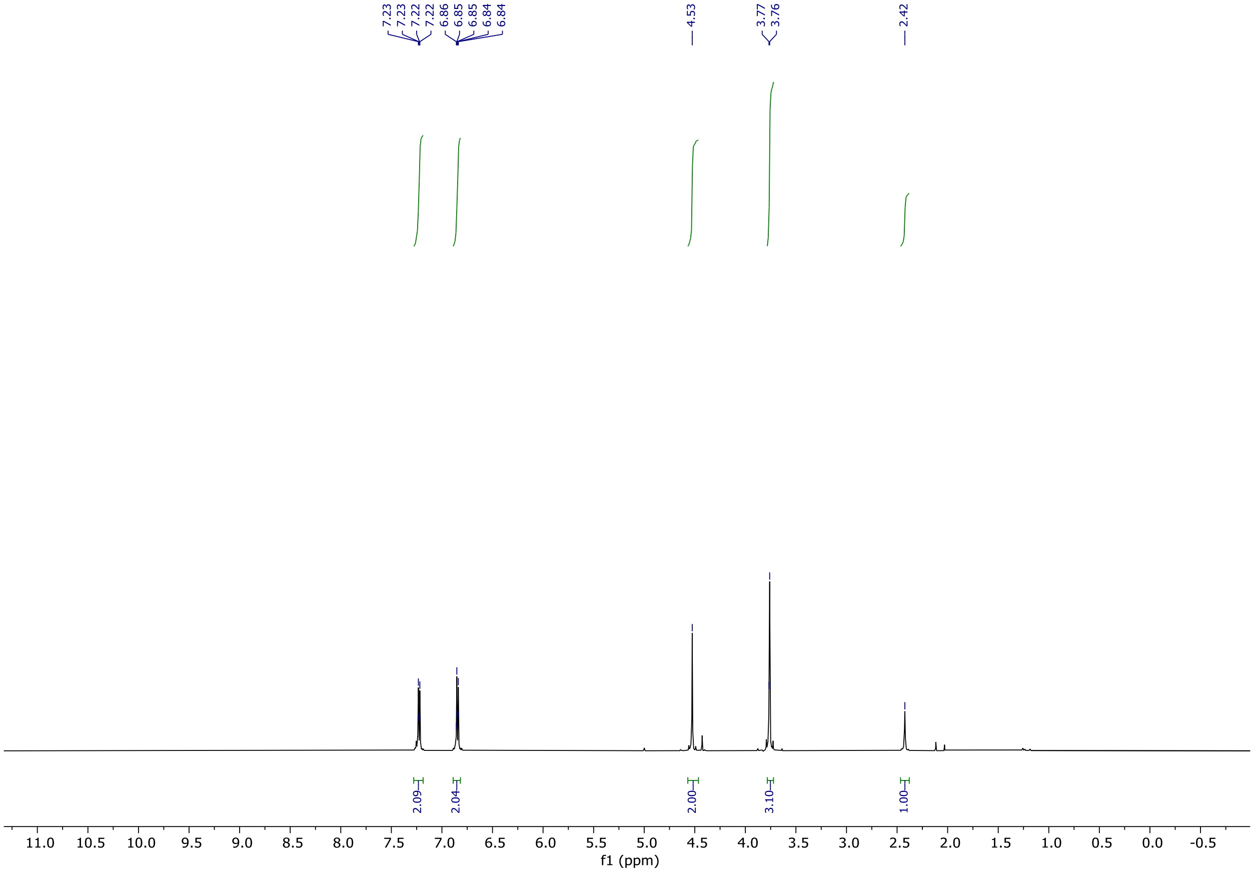

^{13}C NMR (151 MHz, $CDCl_3$):

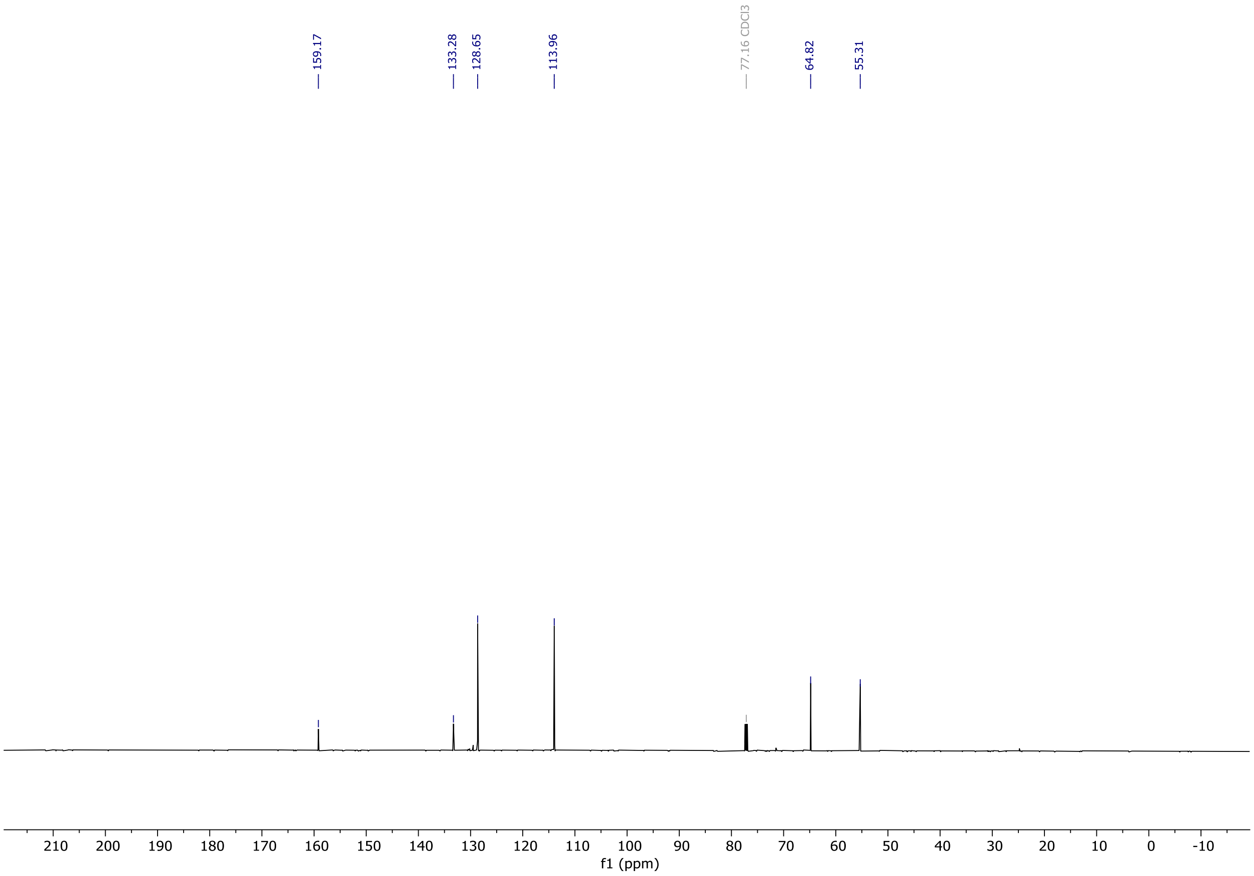

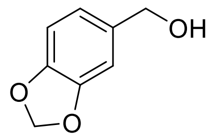

(**3d**)

^{1}H NMR (600 MHz, $CDCl_3$):

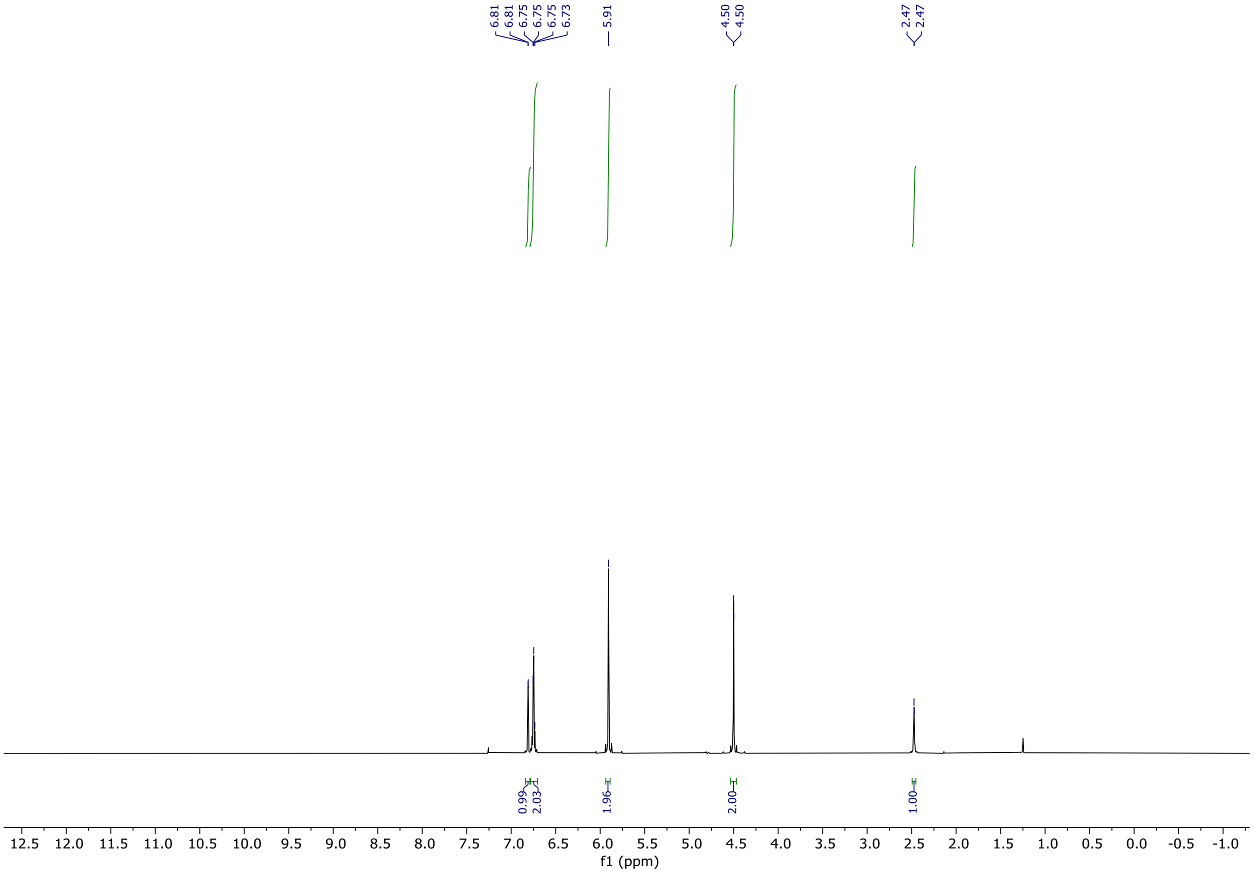

^{13}C NMR (151 MHz, $CDCl_3$):

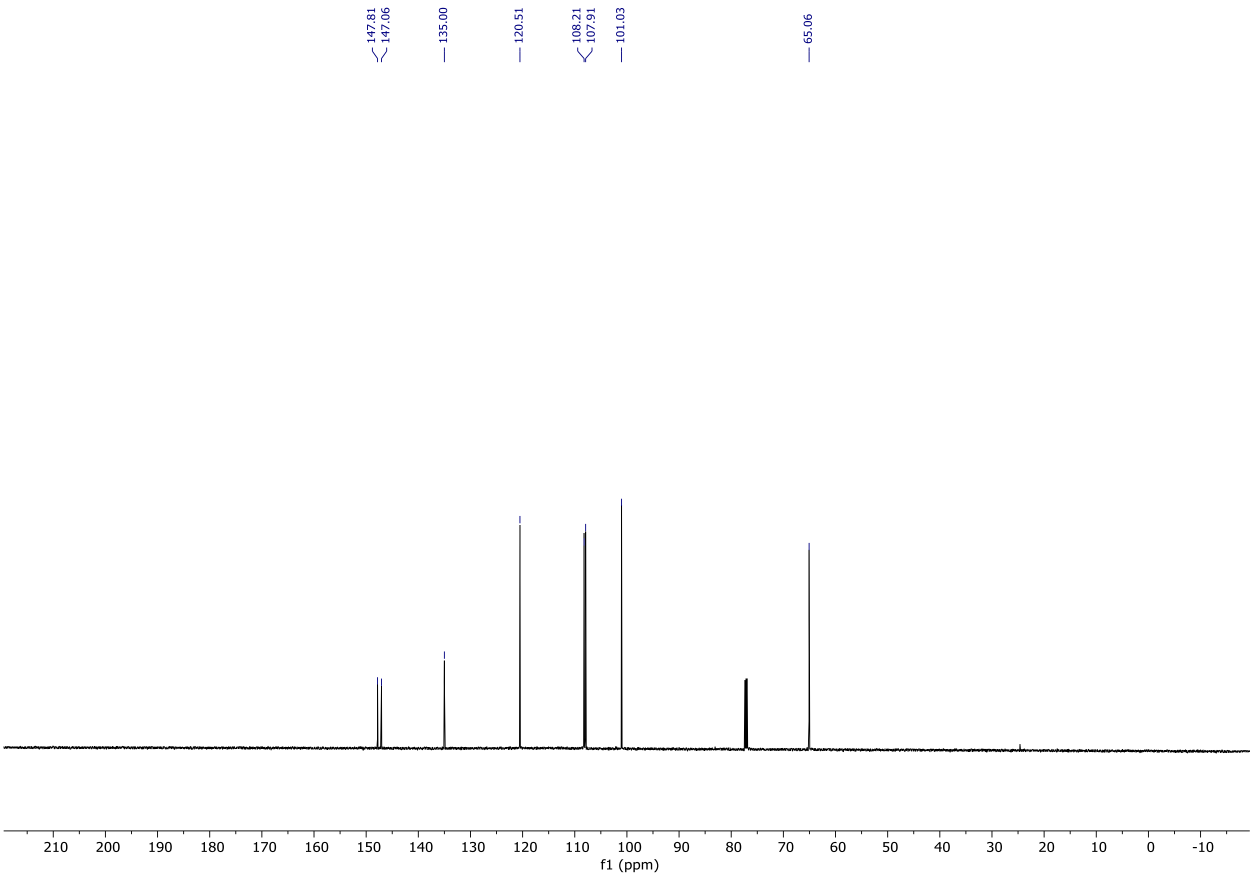

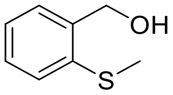

(**3e**)

^{1}H NMR (600 MHz, $CDCl_3$):

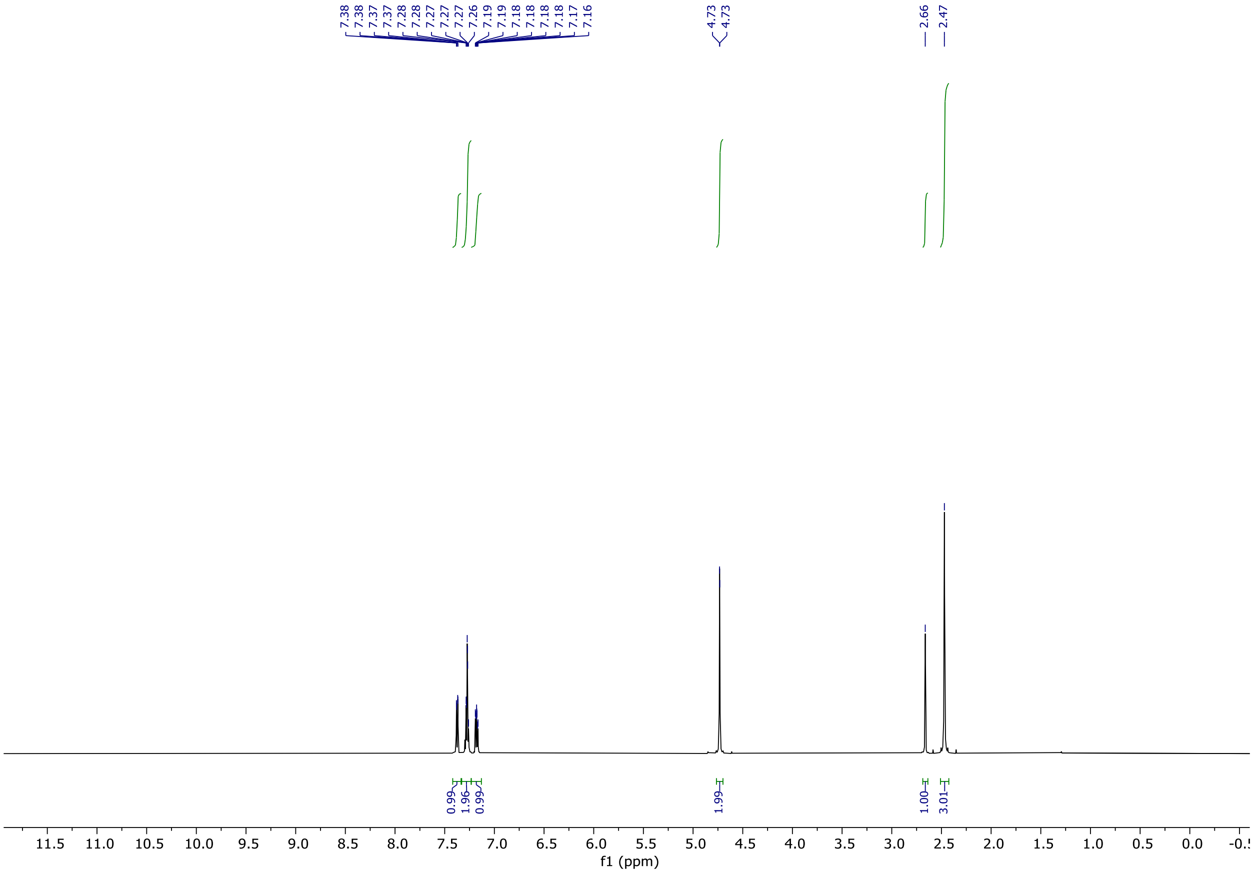

^{13}C NMR (126 MHz, $CDCl_3$):

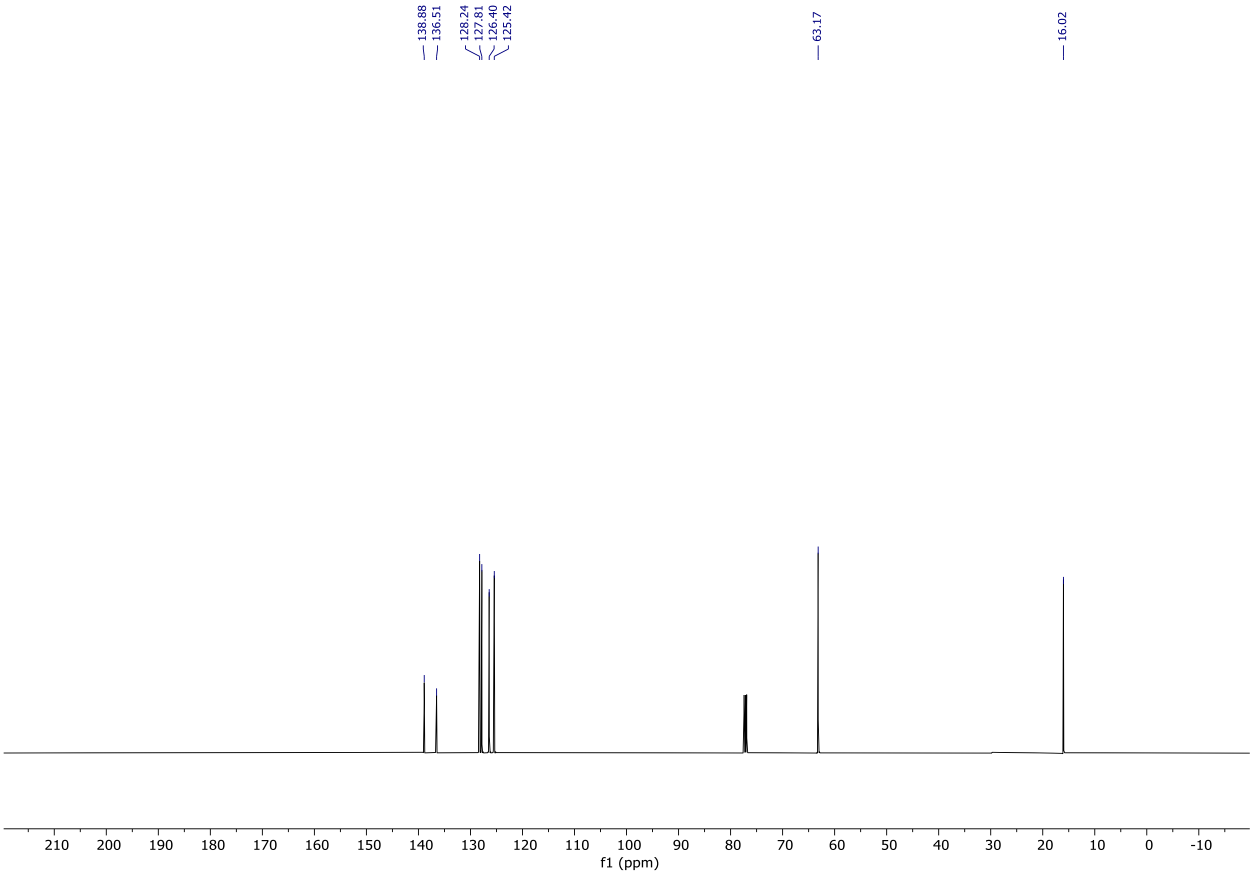

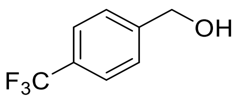

(**3f**)

^{1}H NMR (600 MHz, $CDCl_3$):

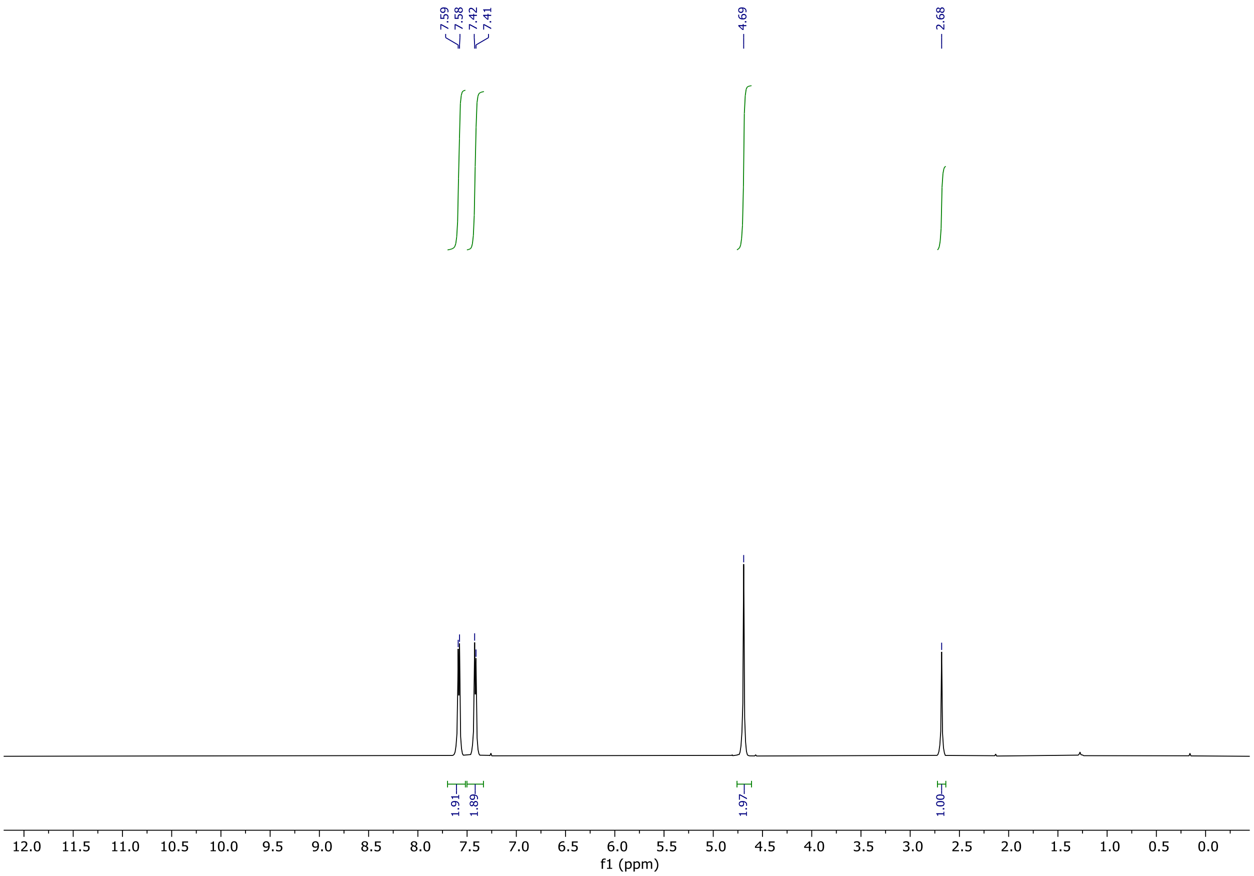

^{13}C NMR (151 MHz, $CDCl_3$):

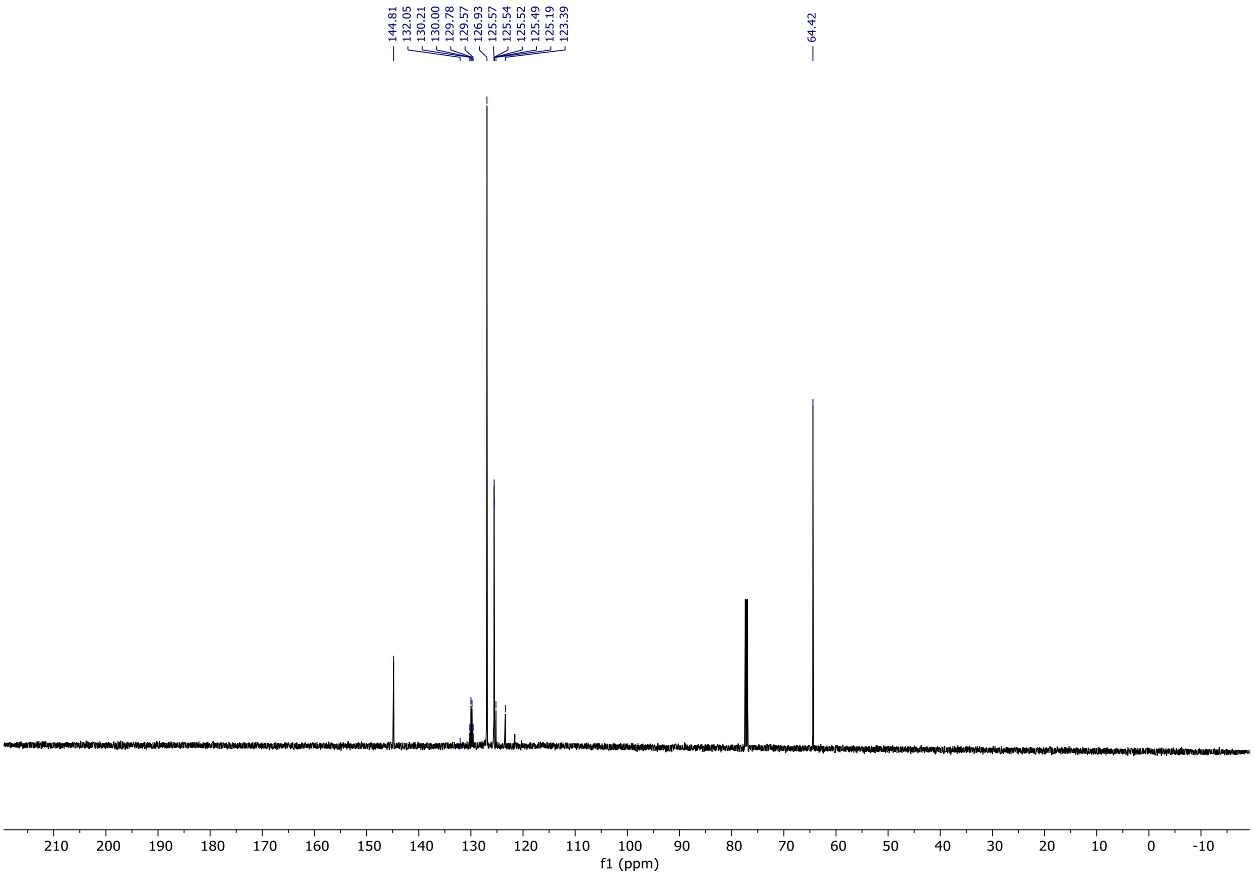

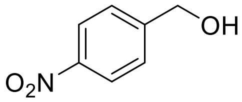

(**3g**)

^{1}H NMR (600 MHz, $CDCl_3$):

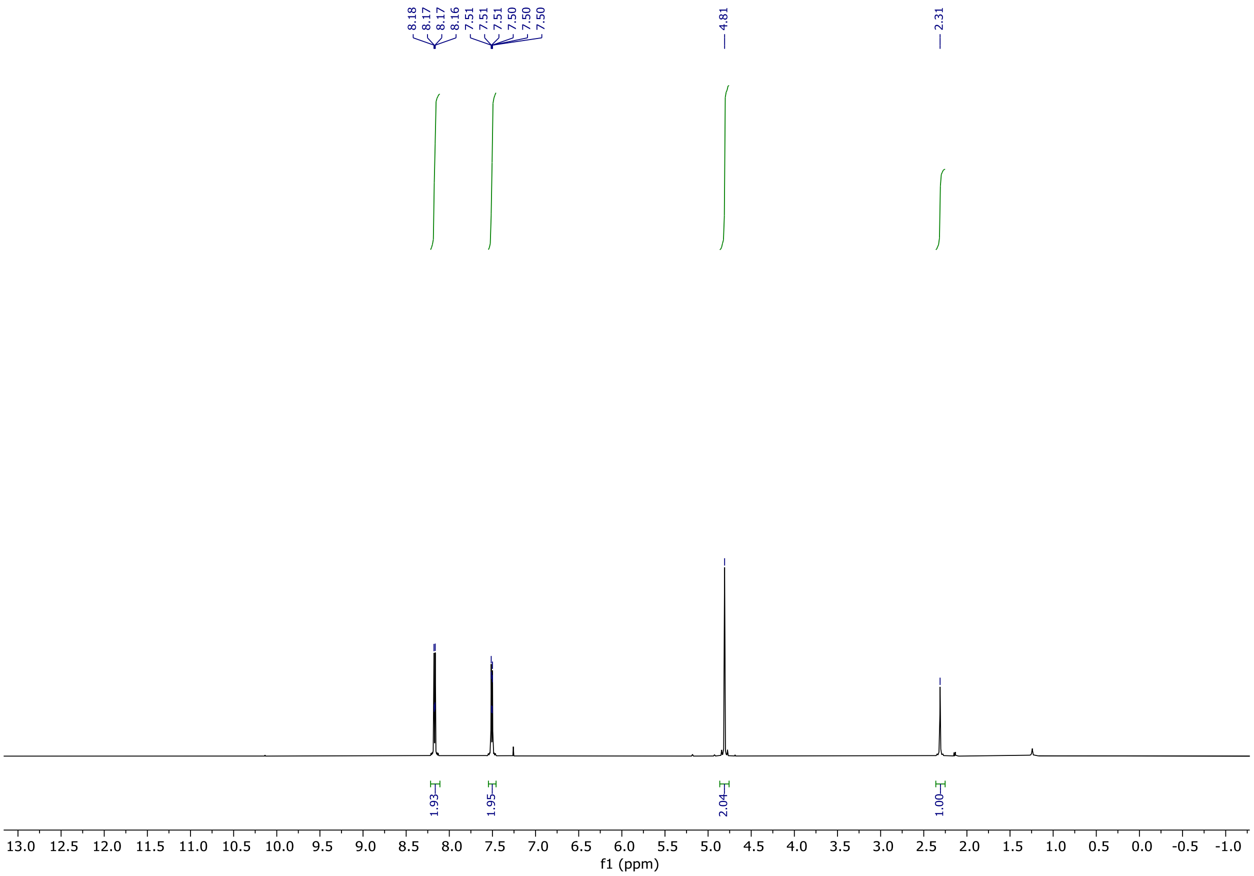

^{13}C NMR (151 MHz, $CDCl_3$):

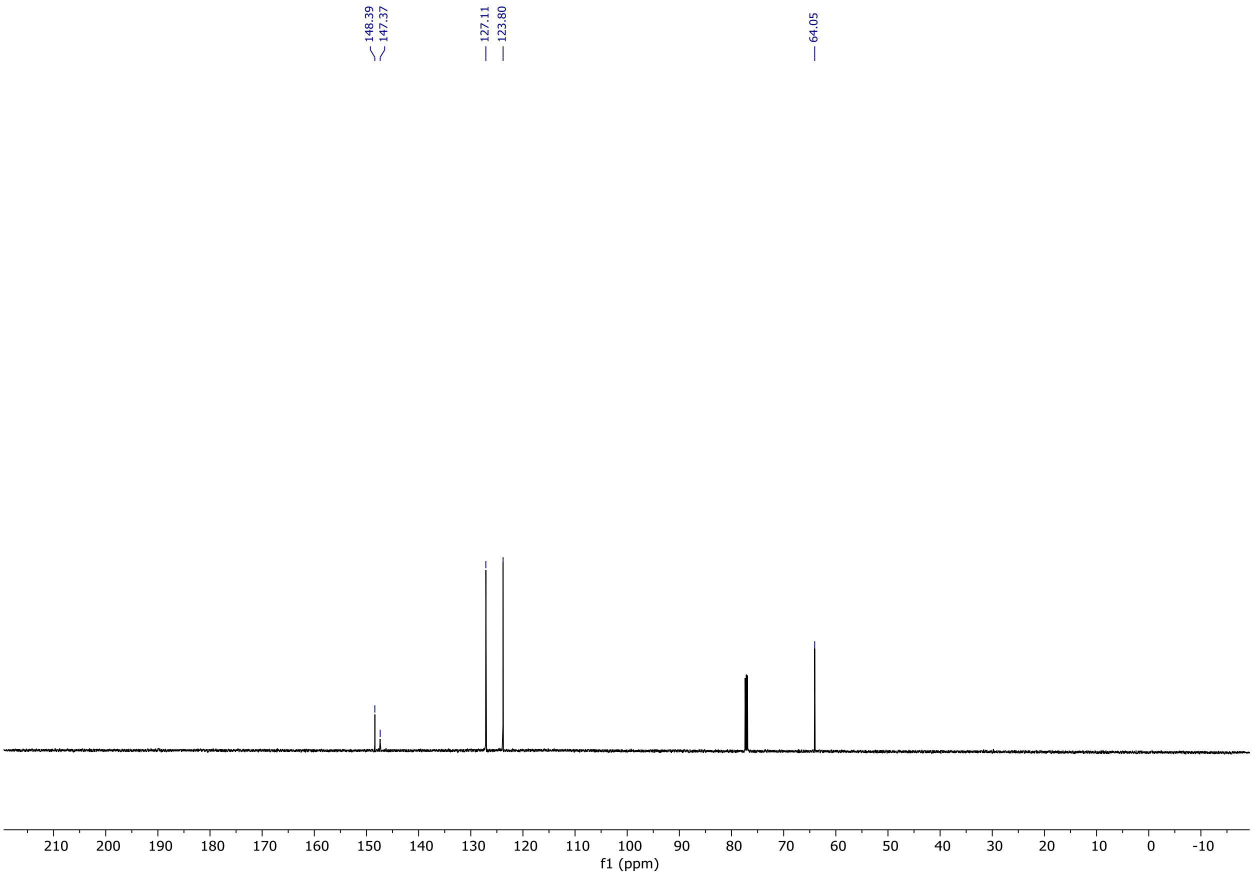

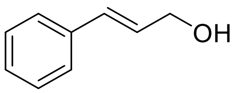

(**3h**)

^{1}H NMR (600 MHz, $CDCl_3$):

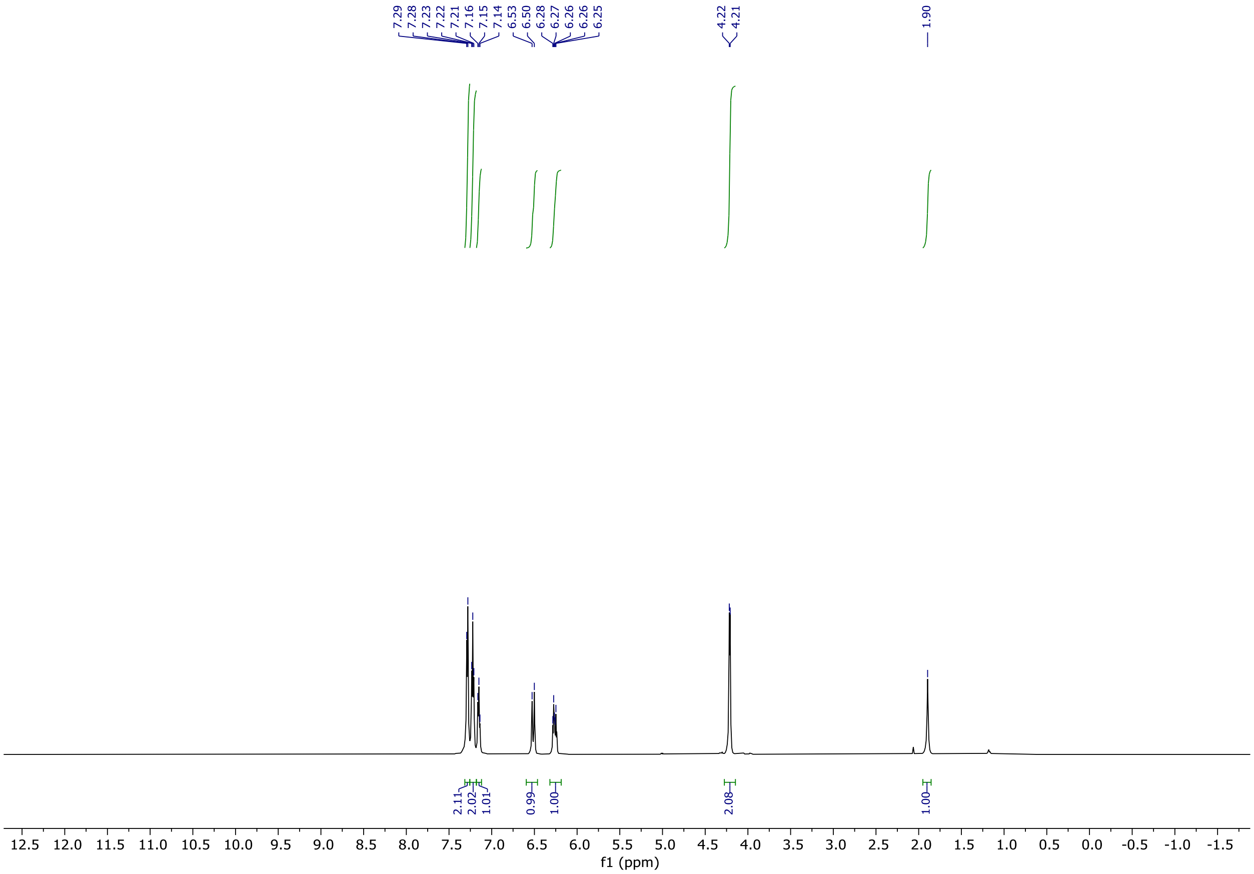

^{13}C NMR (151 MHz, $CDCl_3$):

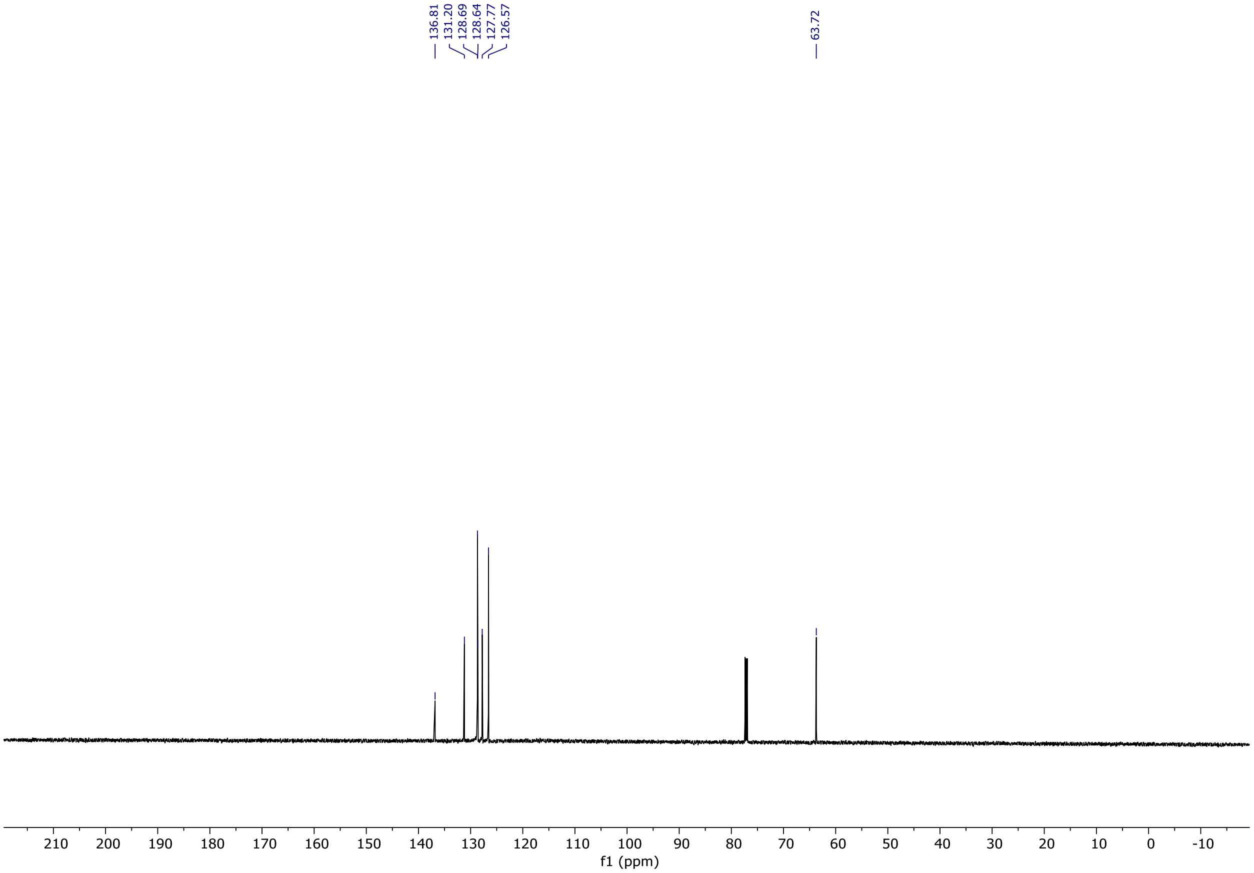

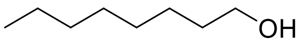

(**3i**)

^{1}H NMR (500 MHz, $CDCl_3$):

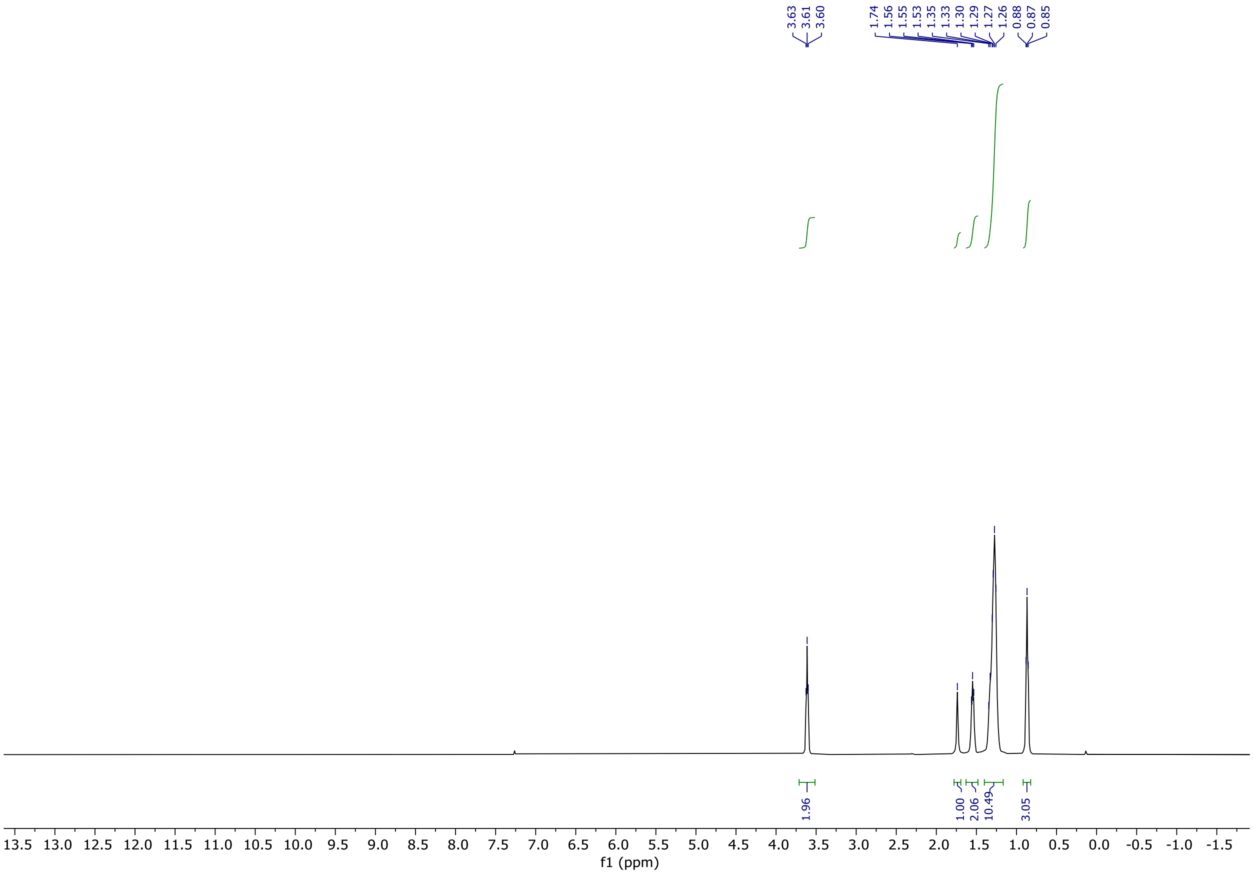

^{13}C NMR (126 MHz, $CDCl_3$):

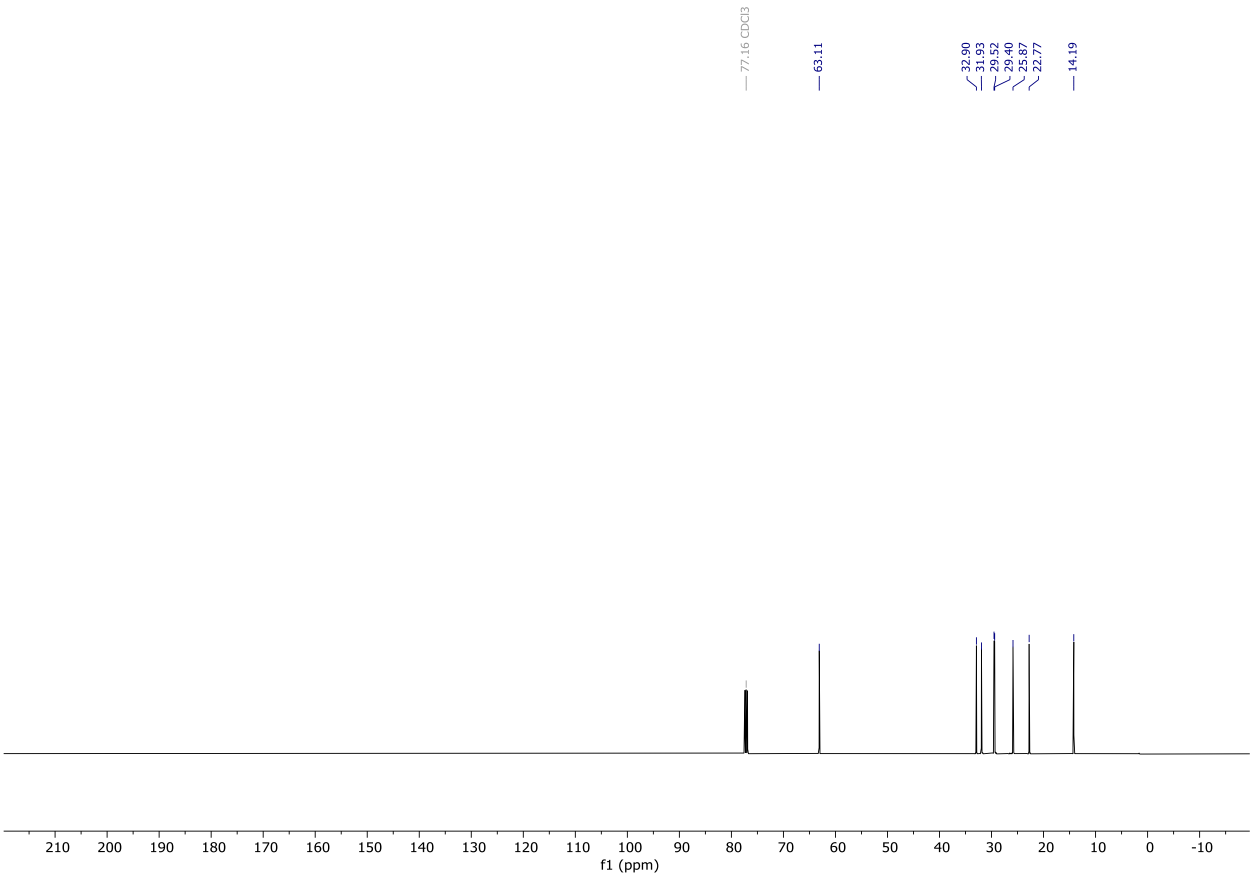

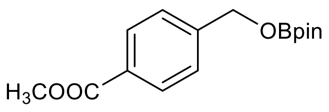

(**4**)

^{1}H NMR (500 MHz, $CDCl_3$):

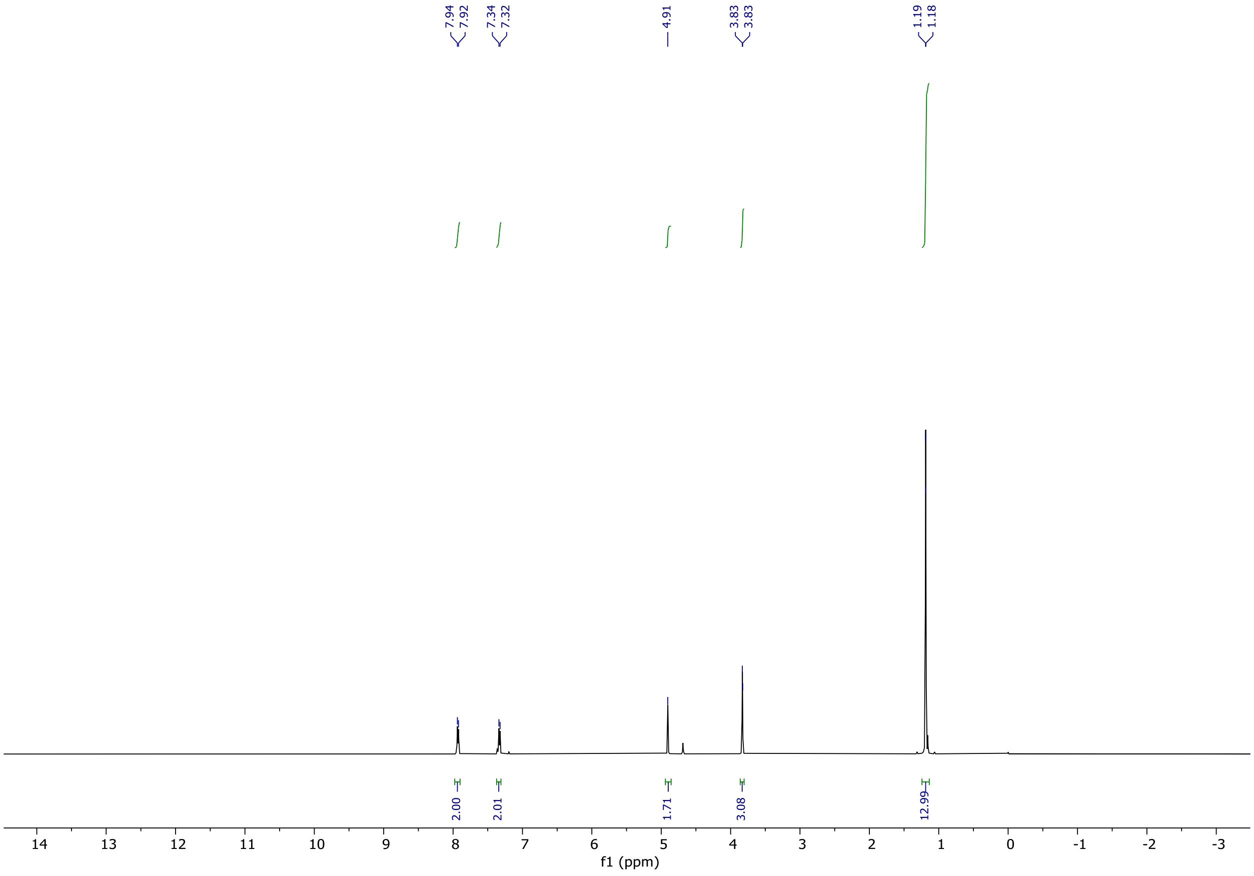

^{13}C NMR (126 MHz, $CDCl_3$):

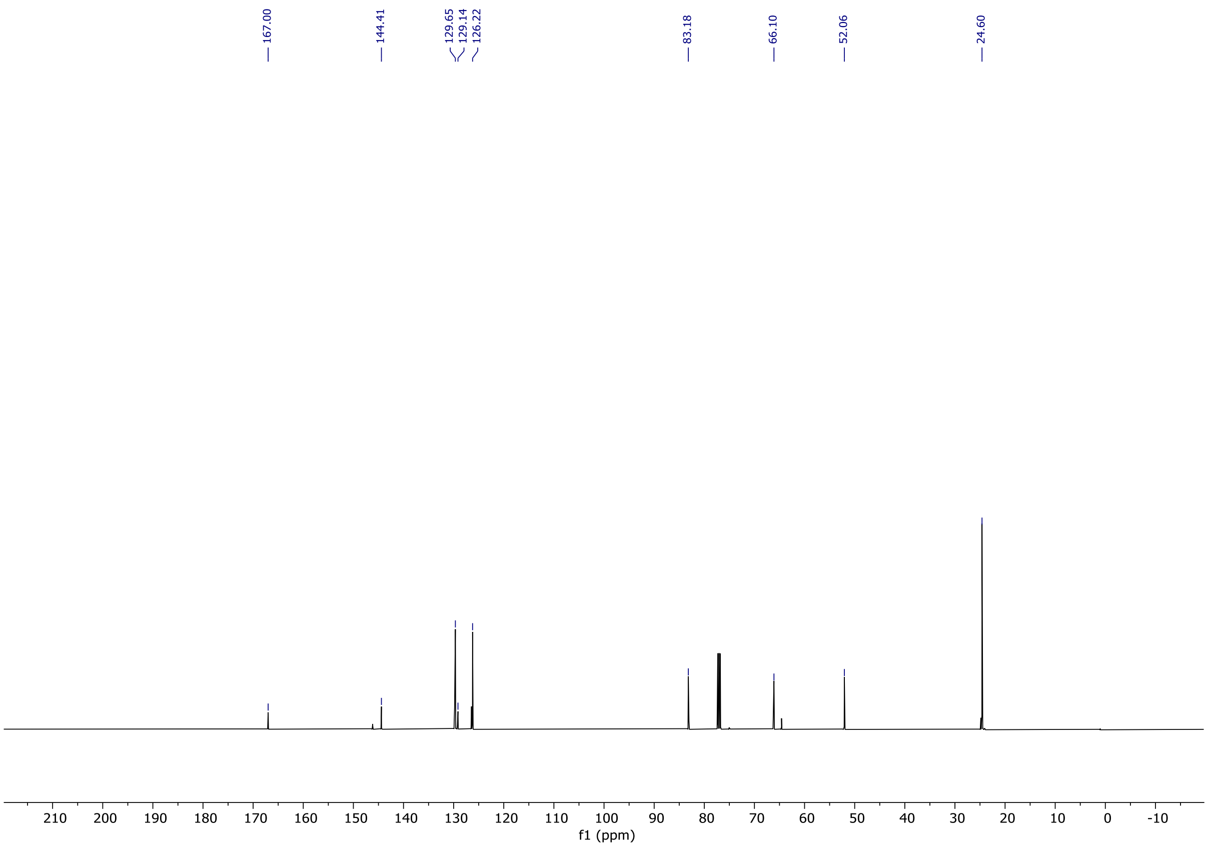

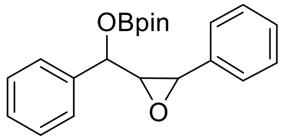

(**5**)

^{1}H NMR (500 MHz, $CDCl_3$):

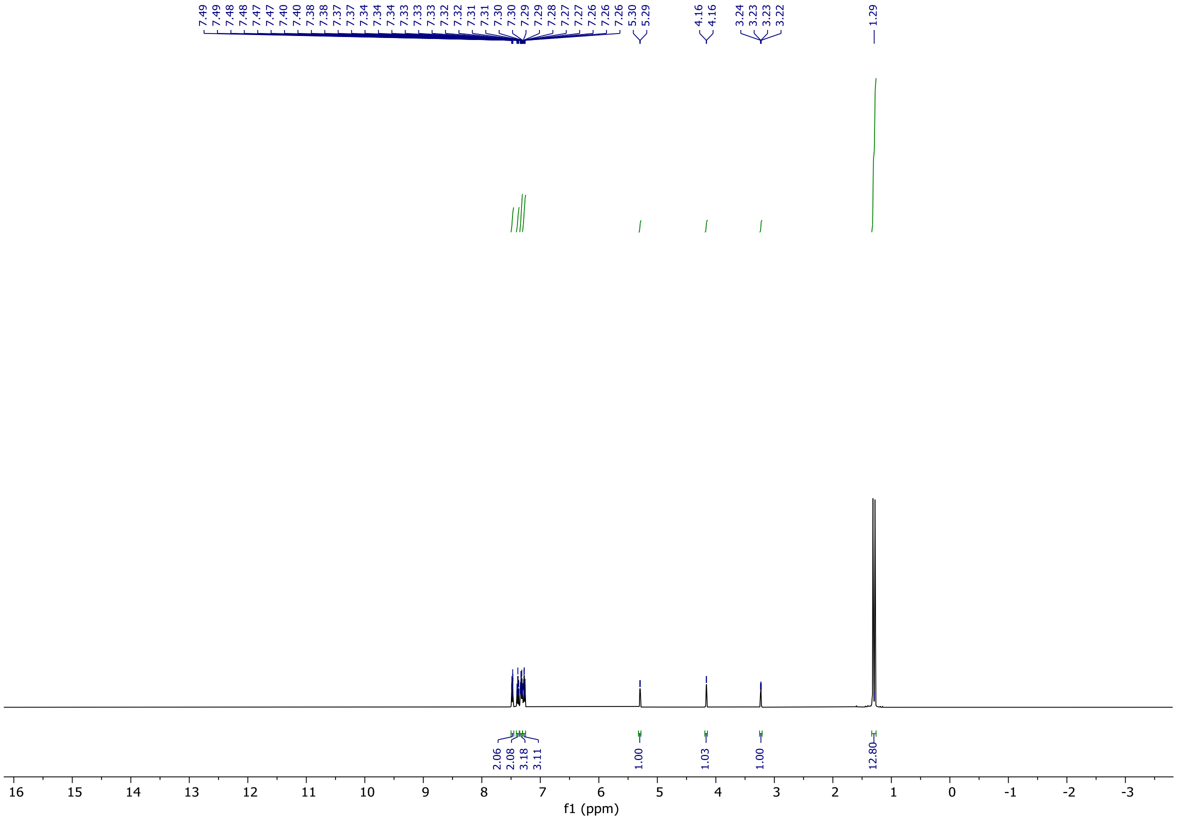

^{13}C NMR (126 MHz, $CDCl_3$):

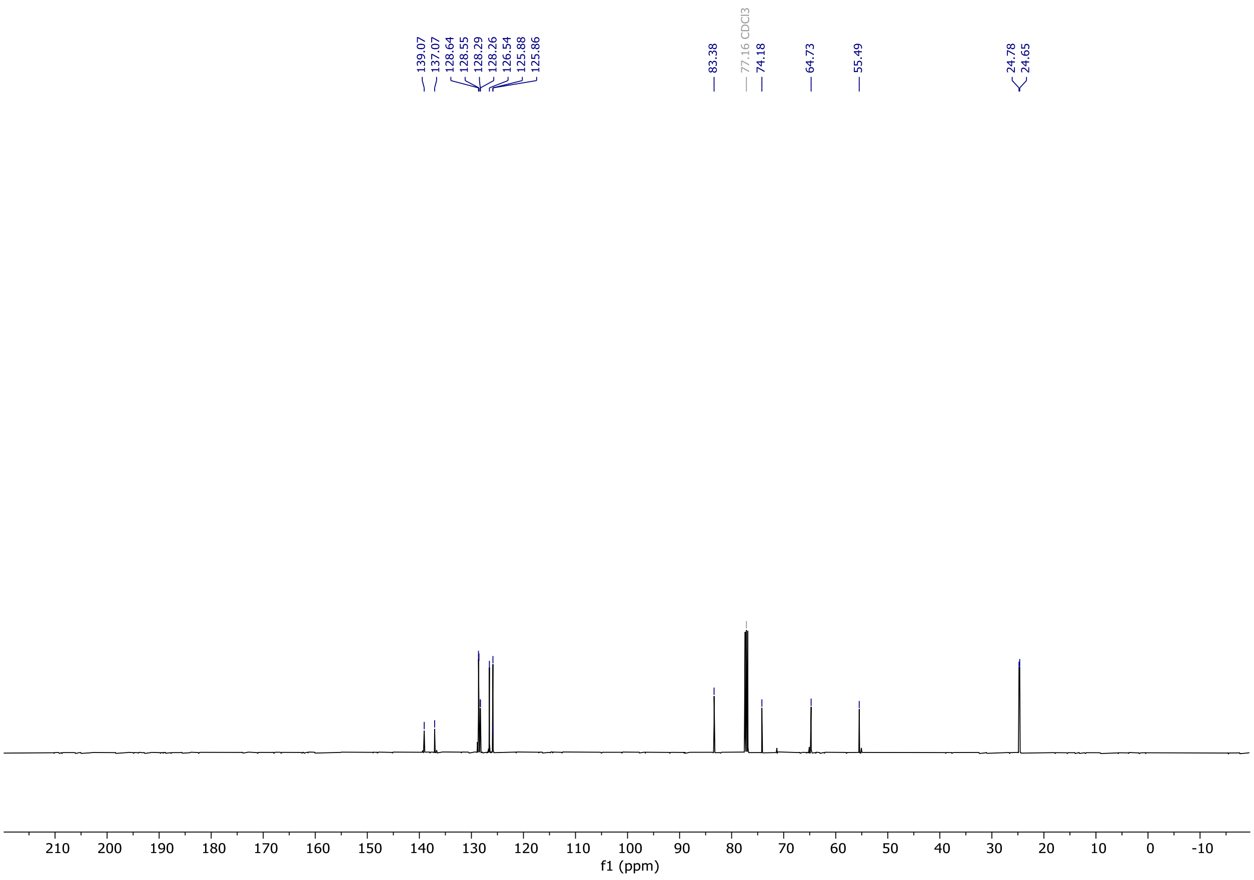

References:

1. (a) Sheldrick, G. M. SHELXTL, An Integrated System for Solving, Refining, and Displaying Crystal Structures from Diffraction Data; University of Göttingen, Göttingen, Federal Republic of Germany, 1981; (b) Sheldrick, G. M. *Acta Cryst.* 2015, *A71*, 3-8.
2. H. Zeng, J. Wu, S. Li, C. Hui, A. Ta, S.-Y. Cheng, S. Zheng, G. Zhang, *Org. Lett.* 2019, **21**, 401.
3. G. Zhang, J. Cheng, K. Davis, M. G. Bonifacio, C. Zajaczkowski, *Green Chem.,* 2019, **21**, 1114.
4. G. Zhang, J. Wu, S. Zheng, M. C. Neary, J. Mao, M. Flores, R. J. Trovitch, P. A. Dub. *J. Am. Chem. Soc.,* 2019, **141**, 15230.
